# Rehabilitation Interventions Delivered via Telehealth to Support Self‐Management of Rheumatic and Musculoskeletal Disease: A Scoping Review

**DOI:** 10.1002/art.43277

**Published:** 2025-08-19

**Authors:** Thomas A. Ingram, Rosemarie Barnett, Nuzhat Shakaib, Simon Jones, Raj Sengupta, Peter C. Rouse

**Affiliations:** ^1^ Department for Health University of Bath Bath United Kingdom; ^2^ Department for Health University of Bath and Royal National Hospital for Rheumatic Diseases, Royal United Hospitals Bath NHS Foundation Trust Bath United Kingdom; ^3^ Department of Computer Science University of Bath Bath United Kingdom; ^4^ Royal National Hospital for Rheumatic Diseases, Royal United Hospitals Bath NHS Foundation Trust Bath United Kingdom

## Abstract

**Objective:**

To identify and summarize existing telerehabilitation interventions for people living with rheumatic and musculoskeletal diseases (RMDs), including the rehabilitation components, the technology used, the type of health care professional (HCP) interaction, and how the effectiveness is evaluated.

**Methods:**

Scopus, Embase, and Web of Science were searched and screened for articles between 2011 and November 2021, and an updated search was completed up to March 2023. The search targeted peer‐reviewed scientific publications involving adults diagnosed with an RMD, which can be considered for self‐management (population), rehabilitation interventions including HCP interaction (concept), and interventions delivered via telehealth for home‐based or outpatient settings (context).

**Results:**

In total, 120 articles fulfilled the inclusion criteria with 84 unique telerehabilitation interventions identified. These interventions most commonly targeted people living with knee osteoarthritis (n = 41) and rheumatoid arthritis (n = 17). Study‐specific web platforms and websites were used in 32 interventions, whereas smartphone applications and social and instant messaging applications were used in 14 and 9 interventions, respectively. Videoconferencing software and services were used to communicate with HCPs in 20 interventions. Physiotherapists had a role in delivering 47 interventions, and audio communication was observed in 43 interventions. Most interventions (n = 44) lasted between 8 and 15 weeks.

**Conclusion:**

A diverse range of digital technologies are being used in the delivery of remote rehabilitation for people living with RMDs. Further studies are required to explore the longevity of telerehabilitation interventions, the optimal delivery methods, and level of HCP contact needed to support people living with RMDs in their self‐management.

## INTRODUCTION

Telehealth, the provision of health care at distance using information and technology,^1^ offers an opportunity to enhance the quality of care that health services provide to people living with rheumatic and musculoskeletal diseases (RMDs).^2^ Yet, the rapid escalation in use of telehealth, enforced by the COVID‐19 pandemic, may have meant a systematic and evidence‐based approach to development, delivery, and evaluation that was not always possible.^3^ Further, the expedited change in RMD health care provision over the past decade has meant the literature lacks a comprehensive synthesis of telehealth rehabilitation interventions. To support researchers, health professionals, and health organizations to improve the future development, delivery, and evaluation of telerehabilitation services, this broad systematic scoping review identifies and describes the existing rehabilitation interventions delivered via telehealth for RMDs.

Telerehabilitation, defined as the delivery of rehabilitation services through information and communication technologies,^4^, ^5^ is at the forefront of RMD care as a result of the COVID‐19 pandemic.^2^, ^6^ Remote, digitally delivered rehabilitation offers convenient health care direct to a patients’ home, reducing the impact of physical limitation, geographic isolation, and expenditure on receiving health care services.^7^, ^8^, ^9^ There is also evidence to suggest that telerehabilitation is cost‐effective^10^, ^11^ and satisfactory to both patients and rehabilitation professionals.^12^ Although telerehabilitation is not a new concept, the recent and rapid popularization has highlighted a range of barriers. For example, health care professional (HCP) training, lack of resources, digital and health literacy skills, lack of physical examination, technical issues, and data security are factors that need to be considered when delivering health care interventions.^7^, ^13^, ^14^, ^15^ Despite these limitations, systematic reviews indicate support for the safety and efficacy of telerehabilitation or remote care across various health conditions, including chronic respiratory disease,^16^ stroke,^17^ heart failure,^18^ musculoskeletal conditions (predominantly including conditions after surgery, such as knee, hip, or shoulder arthroplasty),^19^ and RMDs.^20^

Telehealth has the potential to address some of the challenges facing rheumatology health care. In recent years, a global crisis in the rheumatology workforce has emerged, with the disparity between rheumatology workforces and prevalence of RMDs expected to widen.^21^, ^22^, ^23^ The British Society for Rheumatology have reported rising caseloads, staff shortages, high vacancies, and delays in care, leading to worse health outcomes for patients.^24^ In parallel, the rheumatology community is suffering with burnout and work‐related stress.^25^ The COVID‐19 pandemic exacerbated the situation and led to widespread challenges and adaptations in the management of RMDs.^3^, ^26^ Evidence‐based and codeveloped telehealth interventions provide health services with the opportunity to reduce load on clinicians and health professionals by supporting patients to better self‐manage their RMDs and triage patients in most need of clinician support.

Rehabilitation interventions or remote care delivered via telehealth have shown promise in the care of RMDs^20^ and knee osteoarthritis (KOA) specifically^27^; however, there is a need to provide a detailed synthesis on core intervention elements, such as content, technology used, HCP support, and theories, models, and frameworks used to inform development. For example, it remains unclear which RMDs and behaviors are frequently targeted and which components and digital technologies are used in telerehabilitation interventions. Further, little is known about the type and level of HCP interaction across telerehabilitation interventions for RMDs despite patient interest in direct HCP contact,^28^ the importance of human involvement for intervention adherence,^29^ and the importance of HCP–patient alliance.^12^, ^30^ Telerehabilitation interventions are heterogeneous in nature, and thus it is important to identify how the effectiveness of such interventions are being evaluated. Although a previous systematic review has explored the effectiveness of remote care interventions for RMDs, identified studies predominantly focused on remote monitoring of patients via telephone or video calls (ie, telehealth‐based monitoring of disease activity or function) rather than rehabilitation, and a more detailed review on telerehabilitation interventions is needed.^20^ This scoping review aims to address these literary gaps by identifying and describing the existing telerehabilitation interventions for RMDs and identifying how the effectiveness of such interventions are evaluated. A scoping review was deemed the best approach due to the broad, exploratory nature of this review.^31^, ^32^ Specifically, we aimed to determine (1) the components of rehabilitation delivered via telehealth for RMDs, (2) the technology used to deliver telerehabilitation interventions for RMDs, (3) the type and level of HCP interaction occurring during the delivery of telerehabilitation interventions, and (4) how the effectiveness of telerehabilitation interventions for RMDs is evaluated.

## MATERIALS AND METHODS

### Study design

A scoping review of the literature was performed in accordance with the JBI *Manual for Evidence Synthesis* on Scoping Reviews.^31^, ^32^ This review was reported in accordance with the Preferred Reporting Items for Systematic Reviews and Meta‐Analyses Extension for Scoping Reviews (PRISMA‐ScR) statement (see Table S1, PRISMA‐ScR checklist).^33^, ^34^ Because of the nature of scoping reviews, it is atypical to conduct risk‐of‐bias assessments or critically appraise the methodologic quality of individual studies.^32^, ^35^ Accordingly, critical appraisal and risk‐of‐bias assessments were not performed in this scoping review. PROSPERO does not accommodate the registration of scoping review protocols; however, a protocol for this review has been registered on Figshare^36^ and published.^37^

### Search strategy

To identify suitable articles pertaining to telerehabilitation for RMDs, three electronic databases were searched between 2011 and November 9, 2021: Embase, Scopus, and Web of Science (Core Collection). The search was subsequently updated between 2021 (November 9, when possible) and March 2, 2023. In brief, the search terms were developed based on broad preliminary searches in PubMed and previous systematic reviews on mobile health apps in RMDs,^38^ categorized under population, concept, and context (PCC) headings (population: RMDs; concept: rehabilitation; context: telehealth), refined in collaboration with an experienced subject librarian, and clinically reviewed by a consultant rheumatologist. It should be noted that there are more than 200 different RMDs.^39^ For manageability, the review focused on the major RMDs, which is reflected within the search strategy. Database searching was restricted to English and conducted using truncation, wildcards, proximity searching and “other,” as appropriate (ie, Emtree terms and synonyms). A detailed description of the search strategy has been published.^37^ To identify additional relevant studies, the reference lists of eligible studies were hand searched.

### Eligibility criteria and information sources

For this review, articles were eligible if they involved adults (aged ≥18 years) with a long‐term RMD (diagnosed or referred by a physician or meeting a recognized clinical criteria) that can be considered for self‐management (population). The review focused on articles of rehabilitation interventions that included interaction with an HCP during and/or at the end of the intervention (concept). Interventions that included interaction with individuals in training (ie, student), “exercise professionals” (ie, fitness instructors), and trained “peers” were also included. Articles reporting on interventions that were delivered via telehealth for outpatient or home‐based settings were targeted (context).

Articles were excluded if they involved patients who were unable to make a decision (eg, mental incapacity), were immobile, or were diagnosed with a nonspecific pain disorder (eg, chronic lower back pain or myofascial pain syndrome; population). Interventions targeting a range of chronic conditions that were not of a musculoskeletal or rheumatic nature (ie, diabetes) were ineligible. Articles explicitly stating a self‐reported diagnosis were excluded, unless the participant was assessed by a clinician, met radiographic criteria when appropriate, or the medical records were checked for a clinical code (eg, *International Classification of Diseases*). Interventions developed for the management of injury (eg, fracture), before or after surgery (eg, arthroplasty), or osteoporosis only (a common hallmark of RMDs) were excluded. In addition, telehealth interventions used for disease prevention or diagnosis, or within inpatient settings were excluded (concept). Articles reporting on telehealth interventions without HCP interaction (eg, applications, wearables, sensors, or gamification only) were ineligible (context).

Evidence sources such as peer‐reviewed articles, clinical trials (randomized, controlled), letters, notes, and short surveys were included if they reported sufficient information for review and synthesis. Information sources such as conference proceedings, abstracts, dissertations, and gray literature were not used (see Barnett et al^37^).

### Study selection

Articles identified from each database were combined in Endnote, and duplicates were removed using the Endnote function. The remaining articles were exported into Microsoft Excel, and a pilot test of the eligibility criteria was conducted by the research team.^37^ All titles and abstracts were independently screened by two researchers (NS, TI, or RB), with a third reviewer (PR) discussing and resolving any disputes or discrepancies. The same process occurred for articles forwarded to full‐text review.

Telephone interventions were included, as they may be considered “low‐tech,” but particularly pertinent in settings or populations in which “high‐tech” interactive technologies (eg, videoconferencing) may be inaccessible, unavailable, or too expensive.^40^ Research has also indicated the applicability of employing health education via telephone in populations with low education or heavy economic burden in developing areas.^41^

### Data charting process and synthesis of results

A data extraction template was developed and piloted by two reviewers (NS, RB) to ensure all relevant information was captured.^37^ Data were extracted by three members of the research team (NS, RB, TI). Extracted data included detail on study design, intervention purpose, intervention characteristics (eg, duration and assessment time points), participant characteristics (eg, age, sex, diagnosis, and disease duration), HCP interaction, rehabilitation components, context, and outcome measures. The data were summarized in a narrative manner, with frequency counts of information pertaining to the PCC framework completed.

Articles were also grouped and reported as “primary studies” (ie, an article reporting the main results of an intervention) and “protocol only studies” (ie, an article reporting on a planned intervention with results yet to be published). Primary studies also include pilot or feasibility studies with individual recruitment and methods. “Secondary studies” (ie, protocols relating to published results, secondary analyses, qualitative explorations, or economic evaluations) relating to primary studies are detailed and described narratively (see Table S2) but not reported as an independent study (see Table S3).

## RESULTS

Results are summarized in two large supplementary tables. Table S2 presents the characteristics of selected studies, including author, date, and location; study aims and design; description of participants and of the intervention; type and level of HCP interaction; and the main outcomes assessed. Table S3 provides a high‐level summary of intervention content for each study: specifically, the aspects of rehabilitation targeted (eg, self‐management, physical activity [PA], sleep, occupational health), the type of digital technology used, and the context (eg, face‐to‐face involvement with an HCP, real‐time or delayed HCP interaction, group‐based or social element).

### Study selection

**Figure 1:**
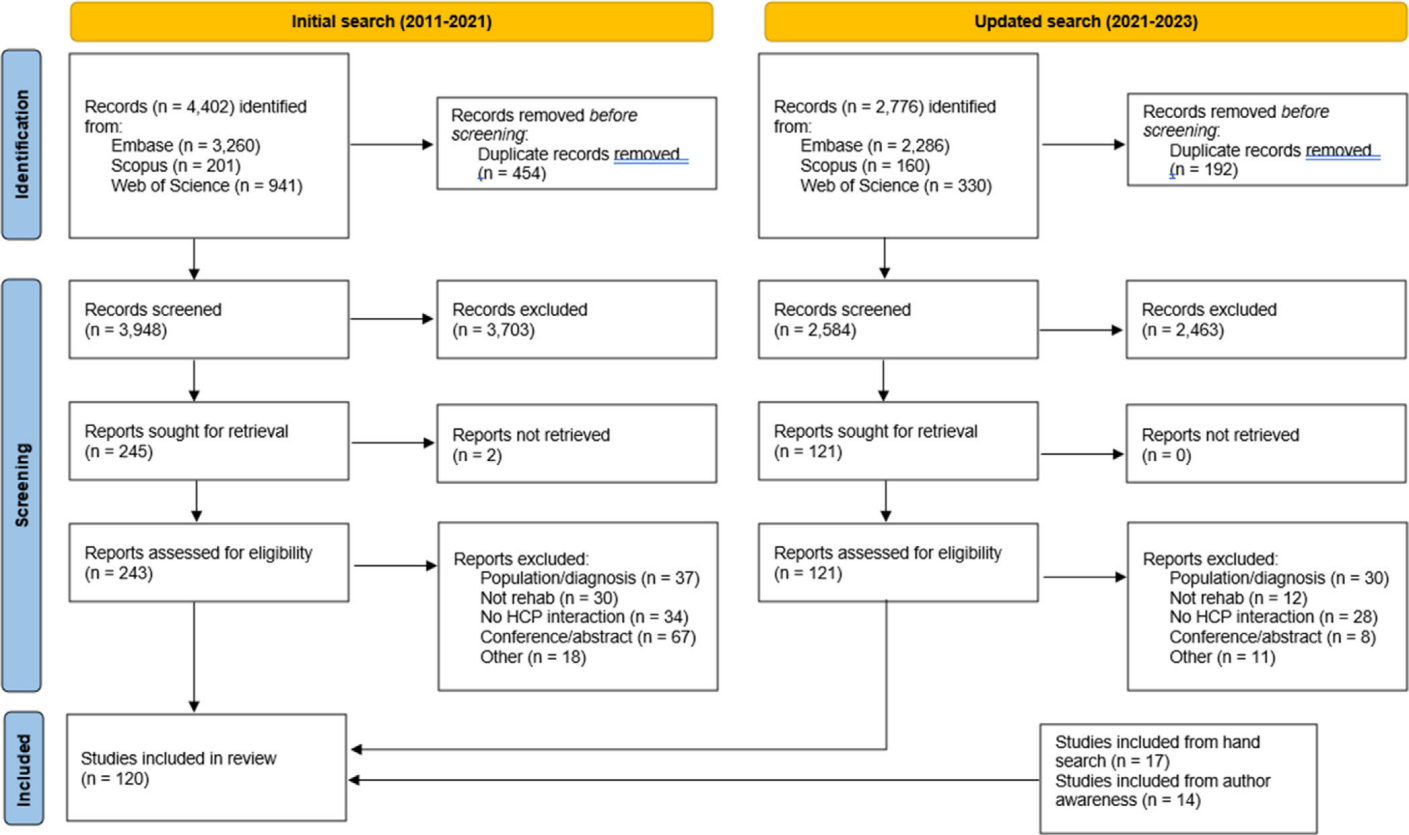
Preferred Reporting Items for Systematic Reviews and Meta‐Analyses flowchart of selection procedure. Records identified from initial search, n = 57. Records identified from updated search, n = 32. Other records identified, n = 31. HCP, health care professional; rehab, rehabilitation.

A PRISMA flowchart^42^ of the study selection process is displayed in Figure 1. The initial literature search (2011 to November 2021) yielded 4,402 articles, of which 57 ultimately met the inclusion criteria. The updated literature search (November 2021 to March 2023) identified 2,776 articles, of which 32 met the inclusion criteria. Across both searches, a further 31 articles were identified and included from hand searching reference lists and authors becoming aware of articles. In total, 120 articles were included.

### Study and participant characteristics

**Figure 2:**
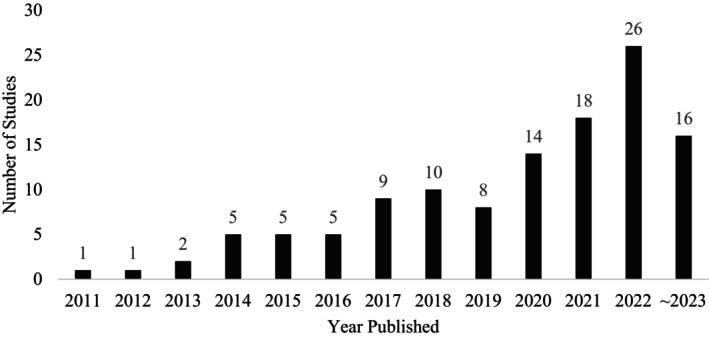
Number of studies published by year (n = 120). The “~” indicates not a full year (ie, the searches were completed to March 2023).

Of the 120 included articles, 62 primary studies and 22 protocol only studies were identified, pertaining to 84 unique telerehabilitation interventions. Thirty‐six articles were classified as secondary studies, in which 15 articles were protocols relating to published results, 4 articles were qualitative explorations, 8 articles were economic evaluations, and 9 articles involved other study designs such as quantitative, multiple‐methods, or secondary analyses. Figure 2 graphically displays the number of articles published by year; most articles were published between 2020 and 2023 (n = 74), whereas few articles were published between 2011 and 2013 (n = 4). A large proportion of articles derived from Europe (n = 45, 37.5%), with 12 of those articles originating from The Netherlands. Articles were also derived from North America (n = 23, 19.2%) and Oceania (n = 23, 19.2%) (see Table 1).

**Table 1:** All included articles by continent (n = 120)

Continent	Articles, n (%)
Africa	2 (2)
Asia	23 (19)
Europe	45 (38)
North America	23 (19)
South America	3 (2)
Oceania	23 (19)
Multiple	1 (1)

The identified telerehabilitation interventions (n = 84) targeted individuals diagnosed with KOA (n = 41), rheumatoid arthritis (RA) (n = 17), fibromyalgia (FM) (n = 7), axial spondyloarthritis (axSpA) (n = 5), systemic sclerosis (SSc) (n = 2), and systemic lupus erythematosus (SLE) (n = 3). The frequencies of targeted populations can be seen in Table 2.

**Table 2:** Population frequencies for primary and protocol only studies (n = 84)*

Diagnosis	Articles, n (%)
axSpA	5 (6)
FM	7 (8)
Generalized OA	1 (1)
Hand OA	4 (4)
Hip OA^a^	8 (9)
Knee OA^a^	41 (45)
OA	1 (1)
IA^b^	1 (1)
Hand RA	1 (1)
RA^a^	17 (19)
SLE	3 (3)
SSc^a^	2 (2)

* axSpA, axial spondyloarthritis; FM, fibromyalgia; IA, inflammatory arthritis; OA, osteoarthritis; RA, rheumatoid arthritis; SLE, systemic lupus erythematosus; SSc, systemic sclerosis.

^a^ Seven studies combined multiple patient populations, six of which included patients with hip and knee OA, and one study included patients with SSc and RA.

^b^ One study included patients with one of the following IA‐related disorders: RA, psoriatic arthritis (PsA), SLE, gout, inflammatory bowel disease (IBD)–related arthritis, or RA + PsA, SLE, or IBD.

The sample size of the primary studies ranged from 3 to 1,059. The mean age of participants ranged from 29.9 to 71.8 years, based on the primary studies reporting mean age (38 of 62 studies). Of the primary studies that presented data on sex (61 of 62 studies), 55 studies (90%) reported higher percentages of female participants (range 51.7%–100%). The results are described according to the 84 unique telerehabilitation interventions identified.

### Aspects and components of telerehabilitation

Fifteen interventions were classified as general education or self‐management interventions only. However, some education was provided to participants (eg, online modules, booklets, telephone, in person) in the majority of interventions (n = 79). A range of telehealth interventions targeted and used PA (eg, physiotherapy, yoga, etc) in the rehabilitation of individuals with RMDs (n = 58). Most studies focused on strengthening and/or neuromuscular exercises, particularly for the lower extremities, which largely reflects the abundance of telehealth interventions targeting knee or hip osteoarthritis (OA). However, strengthening and range of motion exercises for hand dysfunction were used in those with hand OA (n = 4), RA (n = 2), and SSc (n = 1). Examples of other PA interventions include teleyoga for axSpA (n = 2), stretching or mind–body or body awareness exercises for FM (n = 3), power cycling for KOA (n = 3), and walking for RA (n = 2) and KOA (n = 5). Eleven diet or nutrition interventions were identified. These interventions included a self‐education program focused on sodium intake with support from a pharmacist (n = 1), interventions that used a very low‐calorie diet (n = 4), an intervention that used a low‐energy diet (n = 1), interventions that provided general and targeted nutritional information (n = 4), and an intervention that implemented a mediterranean diet (n = 1). A registered dietitian supported participants in eight of these interventions.

Only one telehealth intervention targeted sleep, whereby telephone cognitive behavioral therapy (CBT) for insomnia was used in individuals living with OA. Similarly, only one intervention was identified that supported work functioning (occupation) and consisted of a three‐month eHealth program for individuals with RA. Three RA interventions targeted medication adherence using education delivered via telephone, internet, or mobile application, whereby two interventions were reinforced by nurses and one by a clinical pharmacist.

Four interventions specifically used CBT. Two interventions delivered CBT via telephone for individuals with axSpA and OA, whereas two interventions deployed CBT via the internet (including messaging with a therapist) to individuals living with RA and FM. Fourteen interventions explicitly stated using “counseling.” Of these, nutritional, medication, and psychological counseling were different types of counseling specifically mentioned. Pain coping was indicated in 35 interventions, whereas activity pacing (n = 13) and relaxation techniques (n = 16) were less frequently targeted.

A theory, model, or framework was reported to inform 19 of the identified interventions. Many articles referred to behavior change techniques, yet only seven interventions explicitly stated being informed by a behavior change taxonomy. Despite this, goal setting was used in 34 interventions. Motivational interviewing (including mention of staff training) was a technique employed in 15 interventions.

Despite the interventions having a remote component, participants were given physical resources (ie, information booklets, exercise equipment, device) in 46 interventions. In six interventions, medications or supplements were integrated into the intervention. Medications used in these studies were methotrexate (n = 2), celecoxib (n = 2), glucosamine (n = 2), and supplements (n = 1).

Many interventions were group‐based or involved a social element (ie, videoconferencing; group education or exercise; chat, forum, or discussion board with peers). Specifically, 21 interventions described a social element with peers. Alternatively, the involvement of important others such as family members (ie, for safety, supervision, and support) was compulsory in four interventions.

### Technology used to deliver telerehabilitation interventions

Study‐specific websites, web platforms, or web applications (not including Zoom or YouTube links, etc) were used in 32 interventions. Fourteen interventions employed smartphone applications (not including apps used for data collection only or social apps) to deliver the intervention. Social and instant messaging applications (most frequently WhatsApp and WeChat) were used in nine interventions. Videoconferencing software and services (eg, Zoom, Google Meet, Tencent Meetings, GoToMeeting) were used to communicate with participants in 20 interventions.

Video links (eg, from websites or external links) were commonly used in interventions to provide participants with information or exercise demonstrations (n = 45). Automatic messages, reminders, or notifications (including push content and education, but not messages to HCPs or peers) were also employed in many interventions (n = 32).

Several “other devices” were used in 14 interventions. The other devices incorporated into the interventions included laser acupuncture devices for older adult individuals living with RA (n = 2), Fitbits (n = 6), pedometers (n = 3), a MiBand 4 (n = 1), a multifunctional wearable bracelet (n = 1), a digital wearable medical device (n = 1), and an investigation specific device (n = 1). Further, one intervention employed a virtual reality environment, and one intervention included gamification.

### Type and level of HCP interaction

**Figure 3:**
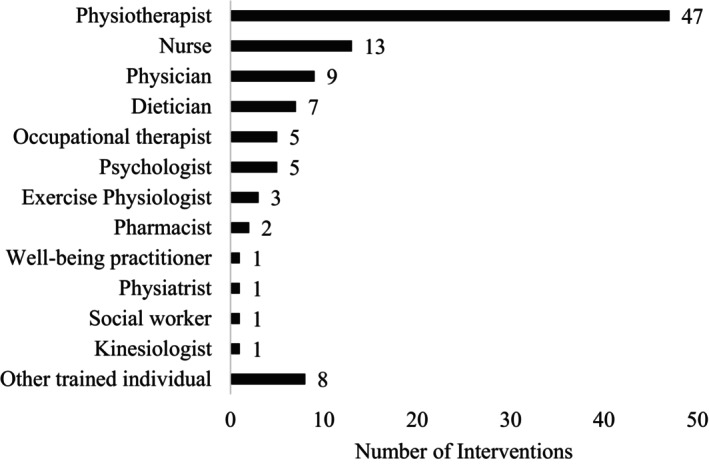
Number of interventions using each type of health care professional. Other trained individuals included master's degree–level psychologist, physical activity coach with a master's degree related to health education, health coach, fitness instructor, or peer with rheumatic and musculoskeletal disease.

Studies were only included in this review if the intervention included interaction with an HCP (Figure 3). Physiotherapists (or physical therapists) were relied on in 47 interventions. Nurses were used in 13 interventions, whereas physicians supported participants in 9 interventions. Other HCPs who were employed included dietitians (n = 7), occupational therapists (n = 5), psychologists (n = 5), exercise physiologists (n = 3), pharmacists (n = 2), psychological well‐being practitioners (n = 1), physiatrists (n = 1), social workers (n = 1), and kinesiologists (n = 1). Eight interventions were supported by trained individuals who may not be considered HCPs (ie, master's degree–level psychologist, PA coach with a master's degree related to health education, health coach, fitness instructor, or peer with RMD). The HCP was unspecified in nine interventions, and in one intervention it could not be determined if the intervention with an HCP was prerecorded.

Participants communicated with HCPs via audio communication (ie, real‐time telephone or smartphone call) in 43 interventions (cannot be determined, n = 4), video communication (ie, real‐time videoconference or video call) in 33 interventions (cannot be determined, n = 4), and text communication (ie, short message service, email, or chat function) in 26 interventions (cannot be determined, n = 5). Participants were able to communicate with HCPs via technology in real time (ie, video, audio, or instant messaging) in 71 interventions. Conversing with HCPs could also be delayed in nature (ie, email, messaging service), with 23 interventions employing this strategy.

Training on the use of technology (eg, HCP, research team, information booklet) was explicitly stated in 38 interventions. Many articles described a face‐to‐face component; however, often there was a lack of clarity about whether this component was part of the intervention. There was a face‐to‐face component (not including consent, assessment, technology training, or providing intervention materials) in 25 interventions, yet this could not be determined in 5 articles. Heterogeneity was observed in the frequency and length of HCP contact; see Table S2 for more details.

### Evaluation of effectiveness

Intervention length (time) varied as follows: less than 8 weeks (n = 13), 8 to 15 weeks (n = 44), 16 to 27 weeks (n = 15), 28 to 39 weeks (n = 2), 40 weeks or more (n = 4), and variable (n = 6). The assessment time points also differed as follows: before and after test only (n = 23), before and after test with midpoint follow‐ups (n = 21), before and after test with follow‐ups after intervention (n = 25), before and after test with midpoint and postintervention follow‐ups (n = 5), variable (n = 3), and other or postintervention only (n = 7).

Frequently used outcome measures are shown in Table 3. Notably, pain was regularly measured via a numeric rating scale (NRS) (n = 31) or a visual analog scale (VAS) (n = 10). Akin to the commonality of OA interventions, disease outcomes were often measured using the Western Ontario and McMaster Universities Osteoarthritis Index (n = 25) or the Knee Injury and Osteoarthritis Outcome Score (n = 12). Performance‐based physical function was measured using the 30‐second chair stand test (30CST) and the Timed Up and Go test in 11 interventions each. Because of the high number of different outcomes measures used, the remaining (nonaggregated) outcomes measures can be seen in Table S2.

**Table 3:** Counts of frequently used outcome measures*

Outcome	Count, n
Disease	
BASDAI	5
DAS28	5
FIQ (including all variations)	7
HOOS	3
KOOS	12
NRS (pain)	31
VAS (pain)	10
WOMAC	25
Performance‐based function	
30CST	11
6MWT^a^	6
TUG test	11
Psychological	
ASES	9
Brief Fear of Movement Scale	4
HADS	6
PCS	8
TSK (including all variations)	4
Physical activity	
Accelerometer/pedometer	13
IPAQ (including all variations)	5
PASE	6
SQUASH	2

* Outcomes grouped by unique intervention (ie, primary articles and protocol only articles), with secondary study outcome measures only being included if not previously mentioned (ie, to avoid duplication). 30CST, 30‐second chair stand test; 6MWT, 6‐minute walk test; ASES, Arthritis Self‐Efficacy Scale; BASDAI, Bath Ankylosing Spondylitis Disease Activity Index; DAS28, Disease Activity Score in 28 joints; FIQ, Fibromyalgia Impact Questionnaire; HADS, Hospital Anxiety and Depression Scale; HOOS, Hips Disability and Osteoarthritis Outcome Score; IPAQ, International Physical Activity Questionnaire; KOOS, Knee Injury and Osteoarthritis Outcome Score; NRS, numeric rating scale; PASE, Physical Activity Scale for the Elderly; PCS, Pain Catastrophizing Scale; SQUASH, Short Questionnaire to Assess Health‐enhancing physical activity; TSK, Tampa Scale of Kinesiophobia; TUG, Timed Up and Go; VAS, visual analog scale; WOMAC, Western Ontario and McMaster Universities Osteoarthritis Index.

^a^ The 40‐m fast‐paced walk test (n = 3), the 40‐m self‐paced walk test (n = 1), and the incremental shuttle walk test (n = 1) were additionally used.

## DISCUSSION

This large scoping review provides a timely and comprehensive synthesis of the literature on the use of telerehabilitation interventions for RMDs, uniquely identifying their content, technology used, level of HCP support, and theories employed to inform their development. This review identified 82 eligible publications over the past five years (2019–2023), compared to 38 in the eight years before (2011–2018); reflecting the recent rapid surge in use of telerehabilitation interventions for RMDs. Diverse approaches were employed within the identified interventions. PA (ie, exercise, yoga, stretching, etc), pain coping, and education were the most common features of telerehabilitation. Websites, platforms, and smartphones applications were the most often used digital technologies, with a rising use of social and instant messaging applications. Physiotherapists and nurses were the most widely used HCP delivering telerehabilitation services to people living with RMDs; however, the type, frequency, and length of HCP interaction was heterogeneous. Most interventions lasted between 8 and 15 weeks, with large heterogeneity in the outcome measures employed to evaluate effectiveness.

Most of the interventions identified provided participants with education (eg, hard copy, online, or verbal), which is in line with the current EULAR recommendations that education is a core element of nonpharmacological management.^7^, ^43^, ^44^ Movement and PA (including physiotherapy) were prominent aspects of remote rehabilitation for RMDs, which may reflect the value and accessibility of conducting PA independently and the focus on managing pain and physical difficulties.^45^ However, the lack of psychological interventions (eg, CBT) is surprising, given the range of psychological challenges experienced by people living with RMDs.^46^, ^47^ Aspects of psychological support were often provided within educational materials (eg, “pain coping” was mentioned in 35 interventions), and mindfulness or relaxation techniques^48^ were often incorporated into yoga interventions. Heterogeneity of behavior change techniques was observed; however, motivational interviewing and goal setting were frequently used to support patients shift to a healthier lifestyle. Despite the use of such techniques, few interventions were informed by or cited a behavior change taxonomy.^49^ Similarly, theoretical frameworks were cited to inform intervention design in less than a quarter of primary or protocol only studies. Using theory (and taxonomies) provides a valuable framework to test health behavior interventions and allows the intervention to be mapped to existing knowledge.^50^ Future studies should describe how they have used theoretical frameworks (and taxonomies) to provide a transparent rationale for the “active ingredients” of the telerehabilitation intervention and incorporate mechanisms of action that test theory and impact sustained behavior.^51^ For example, researchers aiming to test an app‐based intervention could apply and test a recently developed taxonomy of app features based on self‐determination theory.^52^, ^53^

The most common technologies used in telerehabilitation services for RMDs were study‐specific websites or platforms (not including video links to external websites or videoconferencing software). Videoconferencing software (eg, Zoom and Google Meet) was used in almost a quarter of interventions; however, in the past four years, researchers have also employed social and instant messaging applications (eg, WeChat and WhatsApp) to provide participants with intervention material and communicate with HCPs. Social and instant messaging applications are widely available and thus may be cost‐effective and provide efficient HCP–patient contact. Video links were used in more than half of the telerehabilitation interventions, largely for education or exercise demonstration purposes. Only 11 interventions provided participants with wearable devices, which is surprising given the potential benefits in clinical and nonclinical populations.^54^ Other technologies, such as laser acupuncture devices^55^, ^56^ or intervention specific devices,^57^ were rarely provided to participants, which may reflect the specificity of treatment, training needs, or cost to service. Future work is needed to determine the safety profile of using social and instant messaging applications for rehabilitation services and which types of technology lend themselves to greater long‐term self‐management.

Interaction between a patient and HCP is an important component in remote rehabilitation.^58^ This review demonstrated that physiotherapists (and physical therapists) are at the forefront of remote rehabilitation services for RMDs, with more than half of the interventions using their skills. Real‐time communication (eg, telephone, videoconference, instant messaging) with HCPs occurred in most interventions, with delayed contact (eg, messaging service or email) being less frequent. Audio communication (eg, telephone) was most frequently used which reflects both the accessibility of telephonic technology and the use of low‐tech telephonic interventions for individuals with RMDs. More than a quarter of interventions involved in‐person contact with an HCP (not including consent, assessment, technology training, or providing intervention materials), ranging from group education sessions to individual physiotherapy sessions. Incorporating in‐person components to telerehabilitation interventions may help facilitate the transition from in‐person care to home‐based self‐management. For some aspects of rehabilitation (eg, physiotherapy), a hybrid or blended approach may be favorable because patients prefer not to totally replace in‐person sessions.^59^ This review also observed heterogeneity in the type, frequency, and length of HCP contact. For some interventions, the HCP–patient interaction was unclear or poorly reported, limiting the ability to synthesize the data. The optimal level and type of HCP interaction for telerehabilitation interventions for RMDs is currently unknown and is an important future research question.

Remote rehabilitation services are also being delivered by trained individuals who may not be considered HCPs (eg, students, fitness instructors, and peers). In terms of education, self‐management, and social support, trained peers may be an asset to remote support programs,^60^, ^61^ especially in terms of personal experience, cost‐effectiveness, and reducing the burden on health care services. However, future work is needed to determine the scope and effectiveness of peer supported telerehabilitation interventions.

The evidence synthesized in this review demonstrates that rehabilitation interventions delivered via telehealth for RMDs vary greatly in design and length. For example, some research has focused on one day multipoint videoconferencing educational programs,^62^ whereas others have explored the effects of continuously engaging in a digital self‐management program for 12 months.^63^ Just more than one quarter of interventions included follow‐up assessments after the initial pre‐ and postintervention assessments. This lack of follow‐up data highlights a substantial evidence gap, leading to uncertainty regarding adherence, efficacy, and the long‐term utility of self‐management interventions. Future research should consider the inclusion of long‐term follow‐ups, especially for individuals living with RMDs that are often life‐long and progressive in nature.

Telerehabilitation assessments included patient‐reported outcome measures (PROMs) and performance‐based functional measures, that were conducted in‐person or remotely. There appears to be a heterogeneous array of PROMs available for use via telehealth, largely due to differences in the intervention target, disease, or construct measured. Despite the diversity of outcomes, simple and rapid NRS or VAS, were commonly used for the measurement of pain. Many PROMs or clinician completed questionnaires are suitable for remote assessment in telerehabilitation; however, the complexity of some clinical assessments may be too challenging for remote assessment.^64^ That said, interventions identified in this review demonstrate that even performance‐based functional assessments, such as the 30CST,^65^ can be conducted remotely without supervision via video instruction.^63^ Future work could consider the suitability and accuracy of remote telehealth assessment and creating core sets of outcome measures to standardize the evaluation of telerehabilitation interventions.

The identified studies shared a sex imbalance, suggesting the results are more generalizable to female participants than male participants. For example, 55 of 62 primary studies (90%) reported higher percentages of female participants than male participants. However, of the nine studies with 100% female participants, four studies examined FM, which has a high female predominance.^66^ The findings in this review also better reflect interventions targeting individuals with KOA (n = 41, 45%), than those with other RMDs such as axSpA (n = 5, 6%), SLE (n = 3, 3%), or SSc (n = 2, 2%). Generalizability of findings may also be limited because few articles were derived from Africa (n = 2, 2%) or South America (n = 3, 2%). Although critical appraisal or risk‐of‐bias assessment for each evidence source was not conducted for this scoping review,^32^, ^35^ lack of clarity and/or poor reporting hindered the authors ability to succinctly include studies and/or synthesize findings. For example, many studies provided limited information on patient diagnosis (ie, self‐report, confirmed by a clinician, or met classification criteria). Further, there was a lack of clarity in the reporting of the intervention, particularly in terms of behavior change techniques, theoretical frameworks, and HCP interaction (ie, real‐time vs delayed, in‐person component, etc).

This large scoping review uniquely adds to existing reviews by including all types of telerehabilitation interventions for RMDs and providing a detailed synthesis of their content, technology used, HCP support provided, and the theories, models, and frameworks used to inform their development. The protocol for this scoping review has been published, enhancing transparency and rigor.^37^ Although abstracts, conference proceedings, and dissertations were not included and gray literature not reviewed, the search strategy was robust^37^ and reference list scanning of full‐text articles improved coverage. The breadth of eligible articles was also strengthened by the inclusion of protocols and low‐tech telephonic interventions. This review was constrained to telerehabilitation services with HCP involvement; however, innovative information and communication technologies are being used in many aspects of health care. For example, future work could consider reviewing studies focused on telemonitoring,^67^ wearable devices for self‐monitoring,^68^ automated behavior change text messaging,^69^ chatbots,^70^ or machine learning.^71^

Due to the broad, exploratory nature and objectives of this scoping review, a detailed and transparent description of identified interventions in alignment with published guidelines and checklists is not provided, for example, using the consensus on exercise reporting template and template for intervention description and replication guidelines and checklists. This could be explored in future, more targeted literature reviews, for example, which aim to evaluate the effectiveness of reported interventions, or to enable clinician or research replication of effective interventions. It is also important to note that the population of interest for this scoping review was limited to long‐term RMDs that thus require long‐term self‐management. Nonspecific musculoskeletal disorders such as low back pain, acute musculoskeletal disorders as result of injury, or interventions designed to support patients before and after surgery were not captured, as these disorders require different approaches to management, intervention design, and delivery.

The authors note some minimal amendments to the published protocol.^37^ Firstly, a more stringent diagnostic criteria for article inclusion was implemented to improve population accuracy and make the number of identified articles more manageable (see the Methods section). Secondly, sections of the “concept” and “context” summary table (Table S3) have been renamed or added to better reflect the intervention components and improve the accuracy of synthesized results. For example, summary data on behavior change techniques was adapted because of inconsistent reporting and a lack of cited taxonomies.

A diverse range of telerehabilitation services and digital technologies are being used to support and enhance the care of people living with RMDs. Most of the identified interventions were for OA and RA, with few interventions targeting axSpA, SLE, and SSc. Physiotherapists are at the forefront of remote rehabilitation services for RMDs, with more than half of identified interventions using their skills. A lack of clarity in the reporting of methods hinders the synthesis and interpretation of evidence on telerehabilitation interventions for RMDs. Future research is needed to better understand the effectiveness, scalability and longevity of these interventions. An important line of inquiry is to explore the optimal delivery methods and level of HCP contact needed to support people living with RMDs in their self‐management.

## AUTHOR CONTRIBUTIONS

All authors contributed to at least one of the following manuscript preparation roles: conceptualization AND/OR methodology, software, investigation, formal analysis, data curation, visualization, and validation AND drafting or reviewing/editing the final draft. As corresponding author, Dr Rouse confirms that all authors have provided the final approval of the version to be published and takes responsibility for the affirmations regarding article submission (eg, not under consideration by another journal), the integrity of the data presented, and the statements regarding compliance with institutional review board/Declaration of Helsinki requirements.

## Supporting information


**Disclosure Form**:

**Table S2.** Characteristics of the selected studies

**Table S3.** A summary of intervention content.

**Table S1.** Preferred Reporting Items for Systematic reviews and Meta‐Analyses extension for Scoping Reviews (PRISMA‐ScR) Checklist
