# Supplementary material for: Rehabilitation Interventions Delivered via Telehealth to Support Self‐Management of Rheumatic and Musculoskeletal Disease: A Scoping Review
Source: Arthritis Rheumatol. 2025 Aug 19;78(1):26–36. doi: 10.1002/art.43277 (PMC12854011; doi:10.1002/art.43277)
Supplement: Supplementary file 3 — Table S3. A summary of intervention content. [file ART-78-26-s001.docx]

**Table S2**. Characteristics of the selected studies

| **Author / date / location** | **Study aim** | **Study design PLUS length and follow-up** | **Participants (n, age, gender, condition, duration of symptoms)** | **Intervention** | **Type of HCP involved and level of interaction** | **Main outcomes** |
| --- | --- | --- | --- | --- | --- | --- |
| Acar et al. [1]  2023  Turkey | To investigate the effects of tele-yoga on a range of physical and psychological outcomes in people living with AS | Parallel group RCT  8-week intervention  Assessments pre- and post-intervention (baseline and 8 weeks) | AS (n = 55), 24F  Tele-yoga (n = 28), mean age 44.14 (SD 8.03), 13F (46.4%), mean disease duration (years) 13.f93 (SD 7.80)  Control (n = 27), mean age 45.33 (SD 7.24), 11F (40.7%), mean disease duration (years) 13.63 (SD 7.40) | A tele-yoga programme delivered via Zoom videoconferencing.  3 x tele-yoga classes a week for 8 weeks; no self-practice  (Comparator = control / usual activities) | Yoga Alliance registered instructor / physiotherapist  3 x group tele-yoga sessions (60 mins) every week for 8 weeks | Primary: physical function, BASFI  Secondary: disease activity, BASDAI and BAS-G; overall function and health, ASAS HI; spinal mobility, BASMI; flexibility, sit-and-reach test; muscular endurance, sit-up and push-up test; exercise capacity, 1SWT; balance, mCTSIB, LOS, and Neurocom Balance Master test device; sleep quality, PSQI; anxiety and depression, HADS; stress, PSS; QoL, SF-36; mindfulness, MAAS; satisfaction, verbal feedback  Additional: disease activity, ASDAS-CRP; PA, IPAQ-SF |
| Achmad et al. [2]  2022  Indonesia | To investigate the effect of self-education programs for osteoarthritis (SEPO) on sodium intake and serum IL-17a level in people living with KOA | Prospective, multicentre, interventional study  30-day intervention  Assessments at pre- and post-intervention | KOA (n = 80), 32 aged 56-65 years (40%), 74F (92.5%)  SEPO (n = 50), mean age 59.3 (SD 8.7), 45F (90%)  Control (n = 30), mean age 59.67 (SD 7.6), 29F (97%) | A self-education programme  Programme involves an in-person interview, educational resources (i.e., leaflet, poster, handbook), and a WhatsApp chat for chats and videos  (Comparator = control) | Pharmacist:  1 x in-person interview  WhatsApp chat for education and videos sent twice a day apart from Sundays (unclear – probably no interaction) | Primary: sodium intake, SQ-FFQ; pain, stiffness, and function, WOMAC; serum IL-17A, ELISA |
| Adly et al. [3]  2022  Egypt | To explore the effectiveness, convenience, and safety of laser acupuncture, methotrexate, and exercise through teletherapy | Randomized, parallel-assignment, clinical trial  4-week intervention  Assessments pre- and post-intervention | Elderly RA (n = 60), mean age 69 (SD 2.8), 41F  Intervention (n = 30), mean age 68.87 (SD 2.69)  Control (n = 30), mean age 69.13 (SD 2.89) | A 4-week programme of laser acupuncture teletherapy, methotrexate, and aerobic exercise telerehabilitation  (comparator = methotrexate and aerobic exercise telerehabilitation) | Unclear “HCP”  Unclear – training on app  Query or consultation initiation  Videoconference functionality - (level unclear)  HCP can control laser acupuncture parameters | Primary: IL-6 and CRP, ELISA methods; MDA and ATP, spectrophotometric methods.  Secondary: RA QoL, RAQoL; ADL performance, HAQ.  Plus post-teletherapy score sheet for satisfaction, quality and convenience |
| Adly et al. [4]  2021  Egypt | To evaluate laser acupuncture teletherapy for the management of elderly people living with RA | RCT  4-week intervention  Assessments pre- and post-intervention | Elderly RA (n = 60), mean age 67.9 (SD 2.8), 31F (51.7%)  Intervention (n = 30)  Control (n = 30) | A 4-week programme of laser acupuncture teletherapy and telerehabilitation consisting of aerobic exercise and virtual reality  6 x teletherapy sessions per week for 4 weeks  (comparator = aerobic exercise and virtual reality telerehabilitation) | Unclear – nurses and HCP  Query of consultation initiation  Videoconference functionality – text, video or voice message (level unclear)  HCP can control laser acupuncture parameters | RA QoL, RAQoL; ADL performance, HAQ  CRP, IL-6, ATP, and MDA  Plus post-teletherapy score sheet for feedback, benefits and drawbacks |
| Aily et al. [5]  2020  Brazil | To assess if people with KOA adhere to an exercise programme delivered via telerehabilitation | Feasibility study  12-week intervention  Pre- and post-test design. Assessment 1-week post-intervention | KOA (n = 23), mean age 62.0 (SD 15.1), 12F (52.2%) | An exercise programme facilitated via multiple media (DVD, website link, and booklet)  Individual and in-person instruction, exercise sessions at least 3 times a week, physical resources, regular telephone calls  (no comparator) | Investigator/physical therapist:  1 x in-person instruction (unclear who delivered)  6 x telephone calls with investigator (probable PT) for a least 10 mins over 12 weeks | Primary: adherence, Brazilian-Portuguese EARS; adherence and satisfaction, additional questions  Secondary: pain, VAS; function, WOMAC  Experiences, semi-structured interviews |
| Aily et al. [6]  2021  Brazil | To compare the effects periodized circuit training programme delivered via telerehabilitation and in-person for people living with KOA | Protocol of a non-inferiority, parallel, single-blind randomized trial only  14-week intervention  Assessments at baseline, 14 weeks, and 26 weeks | KOA  Target (n = 100) | A periodized circuit training programme delivered by telerehabilitation (DVD, website, *YouTube*, and/or *WhatsApp* for videos  In-person visit, remote circuit training at least 3 times per week, physical resources, regular telephone calls  (Comparator – in-person protocol) | Trained researcher/physical therapist:  1 x pre-intervention, individual, in-person visit  7 x telephone calls over 14 weeks (20 mins) | Primary: pain, VAS; physical function, WOMAC  Secondary: physical function, 40m fast-paced walk test, 30CST, 12 step stair climb test; maximal isometric torque peak of knee extensors, isokinetic dynamometer; thigh composition (muscle mass, mean muscle attenuation, intramuscular fat, and subcutaneous fat), CT scan; body composition (lean body mass, adipose tissue, and bone mineral density, Dual Energy X-ray absorptiometry; muscle thickness, pennation angle, and fascicle length of vastus lateralis muscle, ultrasound device and linear transducer; pain catastrophizing, PCS; adherence, acceptability, and perspective of treatment, self-report and questions |
| Alasfour & Almarwani [7]  2022  Saudi Arabia | Examine the effect of an Arabic ‘My Dear Knee’ app on adherence to a home exercise programme and the effect of the programme pain, function and lower limb strength | 2-arm RCT, parallel study  6-week intervention  Assessments at baseline, week 3, and week 6 | Older women with Knee OA (n = 40), mean age 54.40 (SD 4.33), 40F (100%)  App group (n = 20), mean age 53.65 (SD 3.96), 20F (100%)  Paper group (n = 20), mean age 55.15 (SD 4.64), 20F (100%) | My Dear Knee app  A 6-week home exercise programme supported by ‘My Dear Knee’ app  (comparator = paper group instead of app) | Physical therapist  PT determines exercise schedule  Supervised while performing exercises initially – (unclear if F2F)  Unclear – PT responds to queries | Primary: adherence rate, self-reported (exercise log)  Secondary: pain, Arabic NRS; knee severity (pain and function), ArWOMAC; lower-limb muscle strength, FTSST |
| Allam et al. [8]  2015  Switzerland | To explore the effects of a web-based intervention (information, social support, gamification) on individuals with RA | 5-arm parallel RCT  16-week intervention  Assessments at baseline, 2 months and 4 months | RA (total n = 155), mean age 57.95 (SD 12.29), 71F (45.8%), mean time since first diagnosis 11.89 (SD 11.47)  Information website (n = 30), mean age 55.10 (SD 10.48), 13F (43%), mean time since first diagnosis 9.90 (SD 9.34)  Social support (n = 29), mean age 53.17 (SD 13.29), 3F (10%), mean time since first diagnosis 10.52 (SD 9.83)  Gamification (n = 28), mean age 54.50 (SD 12.01), 4F (14%), mean time since first diagnosis 8.89 (SD 8.22)  Social support plus gamification (n = 28), mean age 53.46 (SD 9.96), 13F (46%), mean time since first diagnosis 20.43 (SD 8.42)  Control (n = 40), mean age 69.33 (SD 6.35), 38F (95%), mean time since first diagnosis 18.11 (SD 15.93) | ONESELF  Group 1: access to informational sections  Group 2: access to informational sections and social support features  Group 3: access to information sections and gaming features  Group 4: access to information sections, social support, and gaming features  Group 5: No access to ONESELF website (control/comparator)  Website use - at least 1 hour per week | Doctor  Social function – 9 prescheduled sessions (over study period) offered in chat room to discuss questions and concerns with a doctor | Primary: PA, Exercise Behaviors Scale; Health care utilization, Health Care Utilization Scale; Prescription medication overuse, Prescription Opioid Misuse Index  Secondary: RA knowledge, Patient Knowledge Questionnaire in RA; Empowerment, 12 items adapted from Spreitzer empowerment scale; |
| Allen et al. [9]  2019  USA | To explore the effectiveness of the STEP-KOA programme on patient outcomes in veterans living with symptomatic knee OA | Protocol for an RCT  9-month intervention period  Assessments at baseline, 3, 6, 9, and 15 months | Knee OA | STEP-KOA  A stepped exercised intervention for people living with knee OA over a 9-month period (Step 1: internet-based exercise programme; Step 2: 3-months of bi-weekly PA coaching calls; Step 3: in-person PT visits)  (Comparator = Arthritis Education control) | Physical activity coaches (Masters Degrees in fields related to health education):  Step 2: bi-weekly telephone calls  Physiotherapist:  Step 3: Between 3 and 7 in-person visits (first 1 hour, remaining 30 mins) | Primary: lower extremity pain, WOMAC  Secondary: physical function, 30CST and 40m fast-paced walk and TUG and stair climbing and 6MWT  Exploratory: PA, PASE; self-efficacy,  Protocol only: Self-efficacy for Exercise scale; social support, Social Support for Exercise Scale |
| Allen et al. [10]  2021  USA |  | RCT  9-month intervention period  Assessments at baseline, 3, 6, and 9 months | Knee OA (total n = 345), mean age 60 (SD 10.3), 53F (15.4%), mean duration of arthritis symptoms (yr) 16.4 (SD 11.2)  STEP-KOA (n = 230), mean age 59.9 (SD 9.9), 36F (15.7%), mean duration of arthritis (yr) 16.3 (SD 11.6)  Control (n = 115, mean age 60.2 (SD 11.1), 17F (14.8%), mean duration of arthritis symptoms (yr) 16.6 (SD 12.4) |  |  |  |
| Kaufman et al. [11]  2022  USA | To evaluate the cost-effectiveness of the STEP-KOA programme at 9-months | Cost-effectiveness of an RCT  9-month intervention period  Assessments at baseline, 3, 6, and 9 months | Knee OA (n = 345), mean age 60 (SD 10.3), 15% F  Intervention (n = 230)  Control (n = 115) |  |  | Costing: ongoing intervention costs, equipment, travel reimbursement, website hosting.  Effectiveness: QoL, EuroQol EQ-5D-5L |
| Azma et al. [12]  2018  Iran | To evaluate the efficacy of a 6-week, home-based, telerehabilitation programme on pain and function in people living with KOA | RCT  6-week intervention  Assessments at baseline, post-intervention (6 weeks), 1 month and 6 month follow-up | KOA (n = 54), mean age 58.25 (SD 7.41), 60.2% F  Intervention (n = 27), mean age 55 (SD 5.2)  Office-based (n = 27), mean age 56 (SD 5.1 | A 6-week, home-exercise programme with weekly telephone control  3 x exercises sessions a week for 6 weeks (i.e., 18) plus hot packs before each session and weekly telephone calls  (comparator = office-based physical therapy) | Medical doctor  Weekly x telephone contact (i.e., x 6)  Plus possible initial in-person interaction at the start to learn exercises and obtain pamphlet and log book (interaction unclear) | Primary: Intensity of knee pain, VAS; disease, pain, ADL, symptom, sport and QoL, KOOS; function, WOMAC |
| Bennell et al. [13]  2021  Australia | Evaluate the additional of telephone coaching to a PT-delivered PA intervention for people with knee OA | Protocol for an RCT  6-month intervention plus assessments at 0, 6, 12, and 18 months | KOA | Addition of telephone coaching to a 6-month PT-prescribed and delivered, home-based, PA intervention for people with knee OA.  PT prescribed progressive home exercise programme. | Physiotherapists:  5 x F2F visits to a PT over 6 months (30-mins each at weeks 1, 3, 7, 12, and 20)  Coaches (nurses, occupational therapist, and health psychologist):  Plus, 6 x telephone-coaching sessions over 6 months (approx. weeks 2, 4, 8, 13, 21, and 25) | Primary: knee pain intensity, NRS; physical function, WOMAC  Secondary: pain on walking, NRS; pain, WOMAC; QoL, AQoL; PA, PASE; PA, Active Australia Survey; PA, activPAL Professional; change in pain and function, global rating of change 7-point scale |
| Bennell et al. [14]  2017  Australia | To explore if the addition of telephone coaching improves the clinical effectiveness of a PT-delivered PA intervention for people with knee OA | 2-arm, parallel design, pragmatic RCT  6-month intervention  Assessments at 0, 6, 12, and 18 months | KOA (total n = 168)  PT and coaching (n = 84), mean age 61.1 (SD 6.9), 57F (68%), symptom duration, years: <2 = 29 (35%), 2-10 = 39 (46%), >10 = 16 (19%)  PT only (n = 84), mean age 63.4 (SD 7.8), 49F (58%), symptom duration, years: <2 = 22 (26%), 2-10 = 44 (52%), >10 = 18 (21%) |  |  |  |
| Bennell et al. [15]  2022  Australia | To evaluate the effectiveness of a 6-month, telehealth, physiotherapist-delivered, dietary weight loss programme combined with exercise in people living with KOA and obesity (or overweight) | Protocol of an RCT only  6-month intervention  Assessments at baseline and 6 months | KOA (overweight/obese)  Target (n = 88) | POWER  A 6-month dietary weight loss and exercise programme, delivered remotely by physiotherapists via videoconferencing  Hard copy education, exercise equipment, home exercise, videoconferencing, very low-calorie diet, and meal replacements  (Comparator = exercise telehealth only) | Physiotherapists:  6 x videoconference (75-mins initially, then 50-mins) over 6 months | Primary: change in body weight, calibrated digital high-capacity scale (Seca 813)  Secondary: BMI, waist circumference, waist-to-hip-ratio; knee pain on walking, NRS; pain, iCOAP; physical function, WOMAC; global change in knee issues, 7-point Likert; HRQoL, AQoL-6D; PA, PASE; internalized weight stigma, WSSQ; physical performance, 30CST, 40m fast-paced walk, 6-step stair climb test; isometric strength of knee extensors, isokinetic dynamometer |
| Bennell et al. [16]  2020  Australia | Evaluate the clinical and cost-effectiveness of telehealth-delivered exercise and dietary weight loss programmes | Protocol of an RCT  6-month intervention with a 12 month-follow-up | KOA (overweight/obese) | Better Knee, Better Me  A 6-month, video-based, telehealth-delivered exercise, and weight loss intervention with online education | Physiotherapist or physiotherapist and dietitian  For exercise only group – 6 x individual video consultation with physiotherapist (initial 45 mins, follow-ups 20 mins)  For Diet and Exercise group – 6 x individual video consultation with dietitian and 6 x individual video consultation with physiotherapist (initial 45 mins, follow-ups 20 mins)  Email availability for queries or problems | Primary: Pain, NRS; physical function, WOMAC  Secondary: weight in kg, self-report; PA, IPEQ-W; HRQoL, AQoL-8D, global rating of change, 7-point Likert; satisfaction with care, 7-point Likert; appointments with orthopaedic surgeon, self-report; depression, anxiety and stress, DASS-21; total knee replacement or arthroscopy, self-report; willingness to undergo surgery, 5-point Likert |
| Bennell et al. [17]  2022  Australia | Evaluate the effectiveness of telehealth-delivered exercise programme, with and without dietary intervention | RCT (3-arm, superiority, pragmatic, parallel design)  6-month intervention  Assessments at baseline, 6 months, and 12-month follow-up (after randomization) | KOA (total n = 415)  Control (n = 67), mean age 65.3 (SD 8.7), 45F (67.2%), median symptom duration (IQR) 4.0 (2.0-10.0)  Exercise (n = 172), mean age 65.4 (SD 8.2), 93F (54.1%), median symptom duration (IQR) 5.0 (2.0-10.0)  Diet and exercise (n = 175), mean age 64.1 (SD 8.1), 89F (50.9%), median symptom duration (IQR) 5.0 (3.0-10.0) |  |  |  |
| Lawford et al. [18]  2021  Australia | Explore patient and Dietitian experiences of a dietary weight loss programme | Qualitative study of an RCT  6-month intervention | KOA (n = 24), mean age 63 (SD 9), 13F (54%) |  |  | Patient and dietitian experiences – semi-structured interview |
| Harris et al. [19]  2023  Australia | Evaluate the cost effectiveness of telehealth-delivered exercise and dietary weight loss programmes within 12-months | Cost-effectiveness analysis of an RCT  6-month intervention with a 12-month follow-up | KOA (overweight/obese) (n = 415) |  |  | Cost of programmes - number of sessions with physiotherapist and dietitian, equipment, meal replacements and portion plate.  Cost of health care-related resource use (hospital inpatient, medications, health services) – custom survey  Direct treatment costs – training clinicals and technology set-up |
| Bossen et al. [20]  2016  The Netherlands | To develop a human-centred eHealth PA intervention for people with knee and hip OA and investigate its feasibility | Development and pilot  Intervention over 12 weeks (development)  Post-intervention interviews | Knee or Hip OA (n = 8), mean age 61.88 (SD 14.53), 6F (75%), duration of symptoms (<1 yr = 2, 1-3 yrs = 1, 3-7 yrs = 2, ≥7 yrs = 3) | e-Exercise  A 12-week, blended exercise intervention with physical therapy | Physical therapist  4x F2F sessions | Programme use – number of modules completed  Satisfaction – SUS  Patient and physical therapists’ experiences - semi-structured interviews |
| Kloek et al. [21]  2014  The Netherlands | To determine the effectiveness and cost-effectiveness of a blended e-Exercise intervention compared to usual care | Protocol of an RCT  12-week intervention and 12-month follow-up | Knee or Hip OA | e-Exercise  A 12-week, blended exercise intervention with physical therapy | Physical therapist  4 x F2F sessions (PTs can plan optional 5^th^ session) over a 12-week intervention  Plus weekly automatic information and reminder emails (not PT) | Primary: physical function, HOOS or KOOS; physical functioning, TUG test; PA, SQUASH; PA, ActiGraph GTx accelerometer  Secondary: pain / symptom / sport / recreation function / QoL, HOOS or KOOS; self-perceived effect, single question; pain and tiredness, NRS; self-efficacy, ASES |
| Kloek et al. [22]  2018  The Netherlands | Investigate the short- and long-term effectiveness of a blended e-Exercise intervention compared to usual care | Cluster RCT  12-week intervention  Assessments at baseline, 3 months (post) and 12-months follow-up | Knee or Hip OA (n = 208), age range 40 – 80, 141F  e-Exercise (n = 109), mean age 63.8 (SD 8.5), 74F (67.9%)  Usual therapy (n = 99), mean age 62.3 (SD 8.9), 67F (67.7%) |  |  |  |
| Kloek et al. [23]  2020  The Netherlands | Explore physiotherapists’ experiences and determinants of usage of e-Exercise | Mixed methods study embedded within a RCT  12-week intervention | Physiotherapists (n = 123) |  |  | Determinants, Questionnaire based on MIDI  PT Experiences, semi-structured interviews |
| Kloek et al. [24]  2018  The Netherlands | Evaluate the cost-effectiveness of a blended e-Exercise intervention compared to usual care | Cost-effectiveness analysis of an RCT  12-week intervention (cost outcomes assessed at 0, 3, 6, and 12 months) | Knee or Hip OA |  |  | Clinical: HRQoL, EQ-5D-3 L; function, HOOS or KOOS; PA, ActiGraph GT3x  Cost: intervention costs, healthcare costs, sports costs, information care costs, absenteeism costs, presenteeism costs, unpaid productivity costs, self-report questionnaire |
| De Vries et al. [25]  2017  The Netherlands | To explore the determinants of adherence to the online component of the blended e-Exercise intervention | Convergent Mixed methods study embedded within a RCT  12-week intervention | Hip ad Knee OA (n = 90 from e-Exercise), mean age 63.6 (SD 8.3), 61F (67.8%), OA duration (< 1 yr = 16 [17.8%], 1-5 yrs = 37 [41.1%], > 5 yrs = 37 [41.1%])  n = 10 for interviews, median age 60 (51-79), 7F (70%) |  |  | Outcome: Adherence, number of weeks graded activity or exercise module was evaluated (1-12) (adherent = >8/12)  Independent: protocol measures assessed at baseline  Experiences, semi-structured interviews |
| Camerini et al. [26]  2013  Switzerland | To assess the effectiveness of an internet-based education programme in people living with FM | Cross-sectional study  Assessment completed after 15 months of website release | FM (n = 209), mean age 49 (SD 10.0), 95% F, mean years of FM 5.6 (SD 4.7) | ONESELF  An online self-management intervention targeting empowerment and knowledge  Website, virtual library for education, virtual gymnasium for tailored multimedia, and laypeople / health professional interaction  (no comparator) | Health professionals:  Interaction via an online forum and chat room (non-specific)  Virtual library – interaction unclear | Online questionnaire  Usage of website, frequency patient visits app, ordinal scale; knowledge of FM, questions adapted from Mayo Clinic; self-management, 5-point scale on PA and drug intake; health outcomes, FIQ |
| Clayton et al. [27]  2015  Canada | To pilot and collect feasibility data to assess the preliminary efficacy of a PA counselling intervention on MVPA in people living with KOA | Protocol for a 2-group pilot RCT (stepped-wedge)  4-week intervention  Assessment at baseline, 5 weeks, and 10 weeks | KOA | TRACK-OA  A community-based PA counselling intervention for individuals living with KOA  An Group based, in-person education and training session, Fitbit Flex monitoring, and telephone follow-up  (Comparator – delayed intervention) | Physiotherapist:  1 x group education and training (inc. individual work with physio on goals and plan) – 1.5 hour  4 x weekly telephone calls for first 4 weeks (20 mins each)  Email contact for questions | Feasibility: recruitment rate, consent rate, dropout rate, adherence to protocol, equipment loss, personnel, location and logistics,  Primary: MVPA, SenseWear mini  Secondary: sedentary time, SenseWear mini; Disease status, KOOS; disease self-management, Partners in Health Scale; treatment effect |
| Li et al. [28]  2017  Canada | To assess the feasibility and preliminary efficacy of a community-based PT counselling intervention for people living with KOA | 2-group, delayed-control, pilot RCT  4-week intervention  Assessments at baseline, 1 month, and 2 months after randomization | KOA (n = 34), mean age 55.5 (SD 8.6), 28F (82%)  Immediate (n = 17), mean age 52.3 (SD 9.7), 14F (82%)  Delayed (n = 17), mean age 58.7 (SD 6.0), 14F (82%) |  |  |  |
| Li et al. [29]  2018  Canada | To assess the efficacy of a technology-enabled PT counselling intervention for improving PA participation, disease activity and self-management capacity in people living with KOA | Proof-of-concept, delayed-control, RCT  8-week intervention  Assessments at baseline, 2 month, 4 months, and 6 months after randomization | KOA (n = 61), mean age 61.7 (SD 8.9), 50F (82%)  Immediate (n = 30), mean age 61.3 (SD 9,4), 22F (73%)  Delayed (n = 31), mean age 62.1 (SD 8.5), 28F (90%) | Monitor-OA  A technology-enabled, PT-led, counselling and PA programme  In-person group education and individual counselling, Fitbit Flex monitoring, and telephone follow-up  (Comparator = delayed control) | Physiotherapist:  1 x 1.5hr in-person group education and individual counselling  4 x biweekly phone calls (20 mins)  Email contact for questions | Primary: MVPA, SenseWear  Secondary: activity in ≥4 MET in bouts of ≥10 min, SenseWear; time spent sedentary, SenseWear; disease status, KOOS; self-management capacity, Partners in Health scale |
| Li et al. [30]  2020  Canada | To assess the efficacy of a 12-week , PT-led counselling and wearable-based intervention on PA participation in people living with KOA | Proof-of-concept, delayed-control, RCT  12-week intervention  Assessments at baseline, week 13, 26, and 39 after randomization | KOA (n = 51), mean age 64.9 (SD 8.5), 42F (82%)  Immediate (n = 26), mean age 65.0 (SD 8), 23F (89%)  Delayed (n = 25), mean age 64.8 (SD 9), 19F (76%) | SuPRA  A PT-led counselling and wearable-based PA intervention  In person group education and individual counselling, Fitbit Flex, and telephone follow-up for 8 weeks. Plus 4 weeks of Fitbit use without counselling calls  (Comparator = delayed control) | Physiotherapist:  1 x in-person group education and individual counselling  4 x biweekly phone calls  Email contact for questions | Primary: MVPA, SenseWear  Secondary: activity in ≥4 MET in bouts of ≥10 min, SenseWear; step count, SenseWear; sedentary time, SenseWear; disease status, KOOS; self-management capacity, Partners in Health Scale; motivation for PA, Theory of Planned Behaviour questionnaire; depression, PHQ-9; habitual behaviour, Self-Report Habit Index |
| Cuperus et al. [31]  2015  The Netherlands | To compare the effectiveness of a multidisciplinary in-person self-management intervention with a telephone-based programme on function in those with GOA | Pragmatic, single-blind randomized clinical superiority trial  6-week intervention  Assessments at baseline, 6, 26, and 52 weeks | GOA (n = 147)  F2F treatment (n = 75), mean age 61 (SD 8), 64F (85%)  Telephone-based treatment (n = 72), mean age 59 (SD 8), 61F (85%) | A multidisciplinary, self-management programme with in-person group sessions and telephone monitoring for people living with GOA  (Comparator = a multidisciplinary 6-week F2F programme) | Physical Therapist and specialized nurse:  2 x in-person group sessions (6-8 patients, 2 hr duration each)  Specialized Rheumatology Nurse  4 x individual telephone contacts (15 min, 30 min, 20 min, 20 min) | Primary: daily function, HAQ-DI  Secondary: HRQoL, SF-36; pain, SF-36 bodily pain subscale; fatigue, subjective fatigue subscale of the CIS; patient specific activity limitations, PSK; 3 most important limitations, 0-10 scale; self-efficacy, GSES; illness cognitions, subscales of the ICQ; fear of movement, TSK; PA, SQUASH; health status, EQ VAS; daily function, 7-point Likert |
| Cuperus et al. [32]  2016  The Netherlands | To evaluate and compare the cost-utility and cost-effectiveness of an in-person, self-management programme with a telephone-based programme for GOA patients | Cost-utility and cost-effectiveness analysis  6-week intervention  Assessments at baseline, 6, 26, and 52 weeks |  |  |  | Utility: general health status, EQ-5D-3L and EQ VAS; QoL, SF-36  Clinical: daily function, HAQ-DI  Costs: treatment programme, medical, non-medical |
| Da Costa et al. [33]  2023  Brazil | To evaluate the effects of a 12-week supervised telehealth stretching programme on pain, functionality, sleep, depression in women living with FM during COVID | Parallel, 2-arm, assessor-blinded, superiority RCT  12-week intervention  Assessments at baseline, 6, and 13 weeks | FM (total n = 28), 28F (100%)  Experimental (n = 13), mean age 49.9 (SD 8.0), 13F (100%), median (IQR) diagnosis time (yrs) 6 (3.5, 15)  Active control (n = 15), mean age 47.0 (SD 6.5), 15F (100%), median (IQR) diagnosis time (yrs) 7 (5, 9) | A 12-week, supervised telehealth stretching programme for women living with FM  (comparator = self-care programme) | Physiotherapist  Supervised stretching session with PT via WhatsApp (video call) (45-50 mins each) – twice a week for 12-weeks | Primary: amplitude of patients’ clinical symptoms, Widespread Pain Index; sleep quality, PSQI  Secondary: symptom severity, Symptom Severity Scale; impact on fibromyalgia on ADLs, FIQ; depression, BDI; pain intensity, VAS; flexibility, one item from Flexitest battery; perception of well-being, single 5-point question  Other: PA, IPAQ-C |
| Dahlberg et al. [34]  2016  Sweden | To explore the effect of a web-based OA self-management programme on joint pain and if patients would recommend it | Pilot  6-week intervention (up to 30 weeks)  Assessments at baseline and 6 weeks (some data every 2-6 weeks and some participants continued to 30 weeks) | Knee or Hip OA (n = 53), mean age 57 (SD 14), 39F (73.58%) | Joint Academy  A web-based OA self-management programme (education and exercise) | Physiotherapist  Asynchronous web-based chat function | Primary: Pain, NRS; recommendation of JA to a friend, custom question (0-10) |
| Nero et al. [35]  2017  Sweden | To evaluate clinical outcomes (e.g., joint pain, physical function, and HRQoL) in patients using Joint Academy | Observational quasi-experimental  6-week intervention  Assessments at baseline and 6 weeks (some data at 3 months) | Knee or Hip OA (n = 350), mean age = 62 (SD 10), 239F (68.3%) |  | Physiotherapist  Asynchronous (and possible synchronous – unclear) web-based chat function  2 x telephone consultations | Primary: Pain, NRS, HRQOL, EQ-5D-3L; physical function, 30CST; difficulty walking, dichotomous response (y/n); afraid of activity, dichotomous response (y/n) |
| Cronström et al. [36]  2019  Sweden | To explore patients’ experiences of a digital self-management programme | Qualitative  Collected after 6-weeks in the programme | Knee or Hip OA (n = 19) mean age 65 (min 45, max 80), 10F (52.6%) |  | Physiotherapist  Asynchronous web-based chat function  Telephone dialogue | Experiences, Semi-structured interviews after 6-weeks |
| Ekman et al. [37]  2020  Sweden | To assess and compare the cost of digital care with F2F care | Costing analysis  Care over a 12-week period | Knee of Hip OA |  |  | Costing domain: health care system (e.g., contacts, sessions, training, administration), patient (e.g., visits, transportation, direct costs), and other |
| Dahlberg et al. [38]  2020  Sweden | To explore the long-term outcomes of participating in a digital self-management programme and the treatment effect duration on pain and function | Observational longitudinal cohort  48-week intervention  Assessments at baseline, 12 weeks, 24 weeks, 36 weeks, and 48 weeks | Knee or Hip OA  0-24 weeks (total n = 499; knee n = 301; hip n = 198), knee mean age 64 (SD 9), knee 372F (75%); hip mean age 63 (SD 9), hip 152F (77%)  0-48 weeks (total n = 138; knee n = 78; hip n = 60), knee mean age 65 (SD 9), knee 54F (69%); hip mean age 64 (SD 8), hip 42F (70%) |  |  | Primary: pain, NRS; physical function, 30CST |
| Jönsson et al. [39]  2022  Sweden | To compare the treatment effects of a digital versus F2F treatment programme after a 3-month period | Retrospective comparative registry-based cohort  3-months on OA programme | Knee or Hip OA  Digital (n = 2709), mean age 63.7 (SD 9.1), 2097F (77%)  F2F (n = 4237), mean age 68.5 (SD 8.6), 2855F (67%) |  | Physiotherapists  Asynchronous web-based chat function  Telephone or video chat dialogue | Primary: change in pain, NRS (0-10)  Secondary: walking difficulties, dichotomous response (y/n); change in QoL, EQ-5D; willingness to undergo surgery, dichotomous response (y/n); fear-avoidance behaviour, dichotomous response (y/n) |
| Farley et al. [40]  2019  USA | To design and implement a pragmatic nurse-led, telephone education programme promoting a T2T approach and shared decision-making in recently diagnosed RA patients | Pragmatic pilot intervention study  Project timeframe – Nov 2015 – Dec 2016 | RA (n = 26), median age 54 (range 22-78), 17F (65%), median months since RA diagnosis 6 (range 0-26 months) | A pragmatic, nurse-led telephone educational programme for individuals with newly diagnosed RA (e.g., understanding diagnosis and nature of RA, functional improvement and monitoring symptoms, keeping appointments etc) | Nurses  1 x telephone call with nurse (20 mins or less) – patients also sent a shared decision-making toolkit | Disease activity, RAPID3  Median number of calls, call time, and qualitive free-text notes  Proportion of RA patients adhering to next rheumatology visit |
| Ferwerda et al. [41]  2017  The Netherlands | To investigate the effects of a tailored internet-based CBT intervention for people living with RA and experience elevated levels of distress | Parallel-group RCT  Intervention length not fixed (varied between 9 and 65 weeks)  Assessments at pre- and post-intervention, plus 3, 6, 9, and 12-month follow-up | RA with elevated distress (n = 133), mean age 56.35 (SD 10.00), 85F (64%)  Control (n = 71), mean age 57.14 (SD 9.36), 47F (66%)  Intervention (n = 62), mean age 55.45 (SD 10.69), 38F (61%) | iCBT  A tailored, therapist-guided, internet-based CBT intervention  At least 1 of 4 modules; goals; assignments; therapist-guided  (comparator = care as usual) | Therapists (master’s degree in clinical psychology):  1 or 2 x F2F intake session – formulation of goals and guided choice of modules / assignments  Weekly or Biweekly x contact via secure messaging service  (research assistant also explained intervention website via telephone – 30 mins) | Primary: psychological function, BDI and IRGL; physical functioning, pain scale of IRGL and fatigue scale of the CIS; impact of RA on daily life, self-care and mobility IRGL and physical and emotion problems of RAND-36 Health Status Inventory  Secondary: disease activity, RADAI; compliance to standard rheumatological care, custom scale; patient evaluation of the intervention, 10-point satisfaction scale, 4-point recommendation scale, and patient preference for mode of deliver. |
| Ferwerda et al. [42]  2018  The Netherlands | To explore the cost-effectiveness of a tailored internet-based CBT intervention for people living with RA and elevated levels of distress | Cost-effectiveness of an RCT  Intervention length not fixed (varied between 9 and 65 weeks)  Assessments at pre- and post-intervention, plus 3, 6, 9, and 12-month follow-up |  |  |  | HRQoL, EQ-5D-3L  Costs: health care use, TiC-P; all medication, self-report medication history; intervention costs (e.g., development, salary of therapists, training, website etc); patient travel costs; loss of productivity, PRODISQ |
| Frade et al. [43]  2023  Australia | To explore the feasibility and effectiveness of a individually supervised, telehealth exercise programme for people living with SLE | Pilot study  8-week intervention  Assessments at baseline and post-intervention | SLE (total n = 15)  Exercise intervention (n = 8), mean age 48 (SD 18), 8F (100%), mean disease duration 12 (SD 8)  Control (n = 7), mean age 41 (SD 11), 7F (100%), mean disease duration 7 (SD 6) | An 8-week (2 days / week, 45 min sessions), supervised, telehealth exercise programme for SLE patients  (Comparator = usual care) | Exercise Physiologist  2 x 45-minute individual and real-time zoom sessions with exercise physiologist per week for 8 weeks | Pain and fatigue, 11-point scale adapted from the 10-point Borg scale; fatigue, FACIT-F; QoL, RAND 36-Item Health Survey (SF-36); lower body endurance, 30sSTS; lower body strength, 5TSTS; upper body endurance, 30sAC; aerobic capacity, 2MST; participant feedback, questionnaire |
| García-Perea et al. [44]  2022  Spain | To assess the effectiveness of online nursing consultations in improving QoL for people living with FM | Parallel design, RCT  12-month study period  Assessments at baseline, 6-month, and 12-month follow-up | FM (n = 80)  Control (n = 40), mean age 55.5 (SD 4.06), 38F (95%)  Intervention (n = 40), mean age 53.3 (SD 7.49), 39F (97.5%) | A web-based platform that provides education and health care professional interaction  Education, clinical history, and messaging system  (Comparator = standard follow-up control) | Nurse or medical staff:  Online messaging system for any time use | Primary: effect of FM on perceived health, FIQ; QoL and emotional status (anxiety and depression), SF-36 |
| Godziuk et al. [45]  2022  Canada | To determine if a weight-neutral behavioural intervention is feasibility and acceptable to KOA patients, and improves body composition and physical function | Protocol of a parallel-arm, randomized pilot and feasibility trial only  12-week intervention (plus 6-months maintenance phase)  Assessments at baseline, 3 months and 9 months | KOA with BMI ≥35 kg/ m^2^ | POMELO  A personalised 12-week, nutrition, arthritis-self management (peer support), and exercise programme largely delivered via videoconference  Nutritional education, peer support and discussion sessions, and whole-body progression exercise programme  (comparator = usual care control) | Dietitian:  1 x Initial consultation to personalize intervention – (unclear – probable one-on-one video consultation)  Biweekly x nutrition education sessions over videoconference (i.e., 6 sessions over 12 weeks) – led by dietitian (unclear if group-based)  Occupational therapist:  Biweekly x peer group support over videoconference (i.e., 6 sessions over 12 weeks)  Exercise physiologist:  1 x one-to-one consultation (unclear if in-person on videoconference)  Optional supervised sessions up to 3 x per week at gym or videoconference  Biweekly x one-to-one sessions via videoconference or telephone | Primary: feasibility, completion rate ≥80%; acceptability, surveys and interviews  Secondary: change in muscle mass, appendicular lean soft tissue; physical function, 30CST and 6MWT  Other: Anthropometrics: height, 235 Heightronic or SECA 264 digital stadiometer; weight, Health-o-meter; BMI; body composition, DXA; resting energy expenditure, open circuit indirect calorimetry; metabolic rate, portable calorimetry device; blood biomarkers (e.g., insulin, CRP, lipids); muscle quality, B mode ultrasound; handgrip strength, hydraulic Jamar handgrip dynamometer; HRQoL, EQ-5D-5L; pain and function, WOMAC; self-efficacy, PROMIS-sf for Managing Daily Activities and Managing Symptoms, and ASES; food and beverage intake over 3 days and PA over 7 days. |
| Godziuk et al. [46]  2023  Canada | To evaluate the acceptability and preliminary effectiveness of a digital behavioural health intervention (nutrition, exercise, and mindfulness) in people living with knee OA | Pragmatic single-arm intervention acceptability study  12-week intervention  Assessments at baseline and 12 weeks (plus interviews) | KOA  Baseline (n = 102), mean age 64 (SD 7), 73.5% F  12-week completers (n = 53), mean age 65 (SD 6.78), 38F (71.7%), symptomatic duration >5 years (n = 35) 66.1% | A 12-week digital behavioural heath intervention comprised of education, mindfulness, nutrition, and exercise components  (no comparator) | Dietitian, psychologist and kinesiologist:  6 x Attendance at live ‘Ask the Expert’ webinar (participants could type in questions)  Dietitian:  1 x 30-min individual call or videoconference | Acceptability: qualitative interviews, participant retention, web-platform usage, number of expert sessions attended  Effectiveness: HRQoL, SF-36; well-being, WEMWBS, mindfulness, FFMQ; self-efficacy, PROMIS Self-Efficacy for Managing Chronic Conditions Short Forms for Managing Daily Activities, and Managing Symptoms; arthritis-specific self-efficacy, ASES; changing in self-report knee OA (0-3), symptom and treatment understanding (0-4), and interest in knee replacement (Y/N) |
| Gohir et al. [47]  2019  UK | To explore whether an internet-based exercise programme can reduce pain perception and sensitivity in people living with KOA | Protocol of an RCT  6-week intervention  Assessment at baseline and 6 weeks | KOA | iBEAT-OA (JA)  A 6-week internet-based physical therapy programme  Daily exercise, PT contact, education, email prompts, and behavioural changes  (Comparator = routine self-management care) | Physiotherapist:  Contact availability via online chat or telephone (as needed) | Primary: pain in last week, NRS  Secondary: pain, stiffness, function, WOMAC; functional performance, TUG and 30 sec sit-to-stand test (also known as 30CST); symptoms and QoL, MSK-HQ; force during an isokinetic maximum voluntary contraction, HUMAC / NORM testing and Rehabilitation System model 7709; tenderness around the knee, pressure pain threshold; central sensitization, temporal summation; function of endogenous pain inhibitory pathways, conditional pain modulation; sleep quality, PSQI; serum biomarkers of inflammation  Also mentioned in protocol: inflammatory markers on ultrasound and muscle thickness assessment of vastus lateralis oblique, MSK-USS |
| Gohir et al. [48]  2021  UK | To compare the effect of an internet-based exercise programme with routine self-management care for people living with KOA | Parallel-group RCT  6-week intervention  Assessment at baseline and 6 weeks | KOA (n = 105), mean age 66.7 (SD 9.2), 71F (67.1%)  Intervention (n = 48), mean age 65.2 (SD 9.7), 34F (70.8%)  Usual care (n = 57), mean age 68.0 (SD 8.6), 37F (64.9%) |  |  |  |
| Hall et al. [49]  2021  Australia | To explore the feasibility of a 6-month digital exercise and weight management programme for people living with hip OA and obesity (or overweight) | Pilot study (non-randomized)  6-month intervention  Assessments at baseline, 3 months, and 6 months | Hip OA (overweight or obese) (n = 18), mean age 64.8 (SD 6.5), 16F (89%), duration of symptoms <1 year (n = 2), 1-2 years (n = 9), 2-5 years (n = 7), >5 years (n = 0) | Modelled on ‘Better Knee, Better Me’  A digital 6-month exercise and diet intervention  Videoconferencing consultations, education, strengthening exercise programme, meal replacements, and exercise equipment  (No comparator) | Physiotherapist:  6 x individual videoconferencing consultations over 6 months (30-45 mins)  Dietitian:  6 x individual videoconferencing consultations over 6 months (30-45 mins) | Adherence, consultations attended and adherence to programme via NRS; acceptability of treatment, NRS; satisfaction, 7-point Likert; hip pain intensity, NRS; physical function, WOMAC; overall change in pain and function, 7-point Likert; weight, self-report on scales; total body mass, fat mas, lean mass and visceral adipose tissue, dual energy-x-ray absorptiometer |
| Hall et al. [50]  2022  Australia | To determine if a 6-month exercise ad weight management intervention improves hip pain for people living with hip OA compared to exercise alone | Protocol of a superiority, 2-group, parallel RCT  6-month intervention  Assessments at baseline, 6 months, and 12 months | Hip OA  Target (n = 100) | ECHO trial  An online 6-month exercise and diet programme  Videoconferencing consultations, hard-copy education, strengthening exercise, meal replacements and exercise equipment  (Comparator = exercise only) | Physiotherapist:  5 x individual videoconferencing consultations over 6 months (30-45 mins)  Dietitian:  6 x individual videoconferencing consultations over 6 months (30-45 mins) | Primary: hip pain severity, NRS  Secondary: body weight, self-report with scales; BMI; total body fat mass and visceral fat mass, dual energy x-ray absorptiometry; hip pain, NRS and exceeding minimal clinically important difference; pain, ADL, and QoL, HOOS; HRQoL, AQoL-8D; global change in PA and hip problems, 7-point Likert  Other: total body lean man, dual energy x-ray absorptiometry; depression, anxiety and stress, DASS; fear of movement, Brief Fear of Movement Scale; medication use and co-interventions, self-report; self-control for eating, Weight Efficacy Lifestyle Questionnaire |
| Hennig et al. [51]  2015  Norway | To evaluate the effect of a informational and home-based hand exercise intervention in women with HOA | RCT  12-week intervention  Assessments at baseline and 3 months | Hand OA (n = 80), mean age 60.8 (SD 7.0), 80F (100%), median (range) symptom duration, yrs 10.0 (0,40), median (range) disease duration, yrs 2.0 (0,21)  Exercise (n = 40), mean age 60.6 (SD 7.9), 40F (100%), median (range) symptom duration, yrs 10.0 (1,40), median (range) disease duration, yrs 2.0 (0,21)  Control (n = 40), mean age 60.9 (SD 6.2), 80F (100%), median (range) symptom duration, yrs 9.0 (0,37), median (range) disease duration, yrs 1.5 (0,16) | A 3-month informational and home-based hand exercise intervention with telephone follow-up  (comparator = information only) | Occupational therapist:  Weekly telephone calls for 1 month (x 4) and every other week for the remaining 2 months (x 4) – for adherence and programme adjustment  Plus telephone number of OT for questions | Primary: change in activity performance, PSFS  Secondary: Fatigue, pain, stiffness and patient global assessment, NRS Maximum grip strength, Grippit electronic instrument; joint mobility, flexion deficit in mm; thumb web space, grip size instrument; activity performance, FIHOA; changes after treatment, OARSI-OMERACT criteria; demographics, joint count, and graded radiography |
| Hernando-Garijo et al. [52]  2021  Spain | To explore the effects of a telerehabilitation programme focused on aerobic exercise on pain, pain sensitivity, FM impact, psychological distress, pain catastrophizing, and function in people living with FM | Single-blind RCT  15-week intervention  Baseline and after the intervention | FM (n = 34), 34F (100%)  Telerehabilitation (n = 17), mean age 51.81 (SD 9.05), 17F (100%)  Control (n = 17), mean age 55.06 (SD 8.51), 17F (100%) | A aerobic exercise telerehabilitation programme  30 video-guided sessions (50 mins) of aerobic exercise over 15 weeks plus weekly video calls  (Comparator = control group) | Physiotherapist:  (unclear supervision of first two sessions before telerehabilitation sessions)  1 x weekly video call contact for 15 weeks | Primary: pain intensity, VAS  Secondary: pain sensitivity, analogical pressure algometer; FM impact, FIQR; pain catastrophizing, PCS; psychological distress, HADS; upper and lower limb physical function, 6MWT and 30sAC |
| Hernando-Garijo et al. [53]  2022  Spain | To investigate the effects of a 15-week aerobic exercise telerehabilitation programme at 6-months follow-up in people living with FM | Secondary analysis of a single-blind RCT  15-week intervention  Baseline and 6-months post-intervention |  |  |  | Primary: pain intensity, VAS; FM impact, FIQR; physical function, 30CST and 6MWT and 30sAC and TUG; elbow and knee isometric strength, Lafayette Instrument model 01165 manual dynamometer; QoL, HAQ |
| Hinman et al. [54]  2020  Australia | To determine if physiotherapist video consultations are non-inferior to in-person consultations for improving knee pain and function in individuals living with KOA | Protocol for an RCT only  3-month intervention  Assessments at baseline, 3 months, and 9 months | KOA  Target (n = 394) | PEAK  A 3-month individualized, home-based, strengthening exercise and PA plan with video consultations  Hard copy educational resources, exercise equipment and video consultations | Physiotherapist:  5 x video consultations over 3 months (30-45 mins each) | Primary: knee pain on walking, NRS; physical function, WOMAC  Secondary: HRQoL, AQoL-6D; PA, PASE; self-efficacy, ASES; perceived global change in pain, function and PA, 7-point Likert; satisfaction with consultations, 7-point Likert; therapeutic alliance, WAI-SF; convenience, NRS; attendance at consultations, log book; adherence with exercise programme, NRS; adherence with PA plan, NRS; co-intervention, custom survey; adverse events, custom survey |
| Hinman et al. [55]  2017  Australia | To evaluate the effectiveness of adding physiotherapist-led telephone-delivered exercise advice and behaviour change support to an existing nurse-led telephone service for people living with KOA | Protocol of an RCT  6-month intervention  Assessments at baseline, 6, and 12 months | KOA | Adding a physiotherapist-led exercise advice and support telephone intervention to an existing nurse-led telephone service over a 6-month period  Education, devise goals and action plans, home-based strengthening exercise programme and PA plan  (Comparator = existing nurse-led telephone service) | Nurse:  1 or more x telephone call (existing)  Physiotherapist:  5-10 x telephone call (exercise advice and support) – initial calls were 40 minutes, and follow-up calls 20 minutes | Primary: average knee pain, NRS; physical functioning, WOMAC  Secondary: knee pain on ADLS, WOMAC; knee pain on walking, NRS; self-efficacy for pain and function, ASES; fear of movement, Brief Fear of Movement Scale; PA, PASE; behavioural determinants of exercise, Barriers to (and Benefits of) Physical Activity Scale; HRQoL, AQoL-8D; global changes (overall, pain, function and PA), 7-point scales; satisfaction, 7-point scale  Protocol only: work productivity, WHO HPQ Short form |
| Hinman et al. [56]  2020  Australia |  | Pragmatic superiority parallel-group RCT  6-month intervention  Assessments at baseline, 6, and 12 months | KOA (n = 175)  Existing (n = 88), mean age 62.5 (SD 8.1), 55F (63%), mean symptom duration, yrs 9 SD 8)  Exercise advice and support (n = 87), mean age 62. (SD 9.1), 55F (63%), mean symptom duration, yrs 10 (SD 9) |  |  |  |
| Lawford et al. [57]  2018  Australia | To explore KOA patients’ perceptions of telephone-delivered exercise therapy by a physiotherapist | Qualitative study nested within an RCT  6-month intervention  Post-intervention assessment | KOA (n = 20), mean age 59 (SD 9), 13F (65%) |  |  | Patient perception, post-intervention semi-structured interviews |
| Lawford et al. [58]  2019  Australia | To explore physiotherapists’ perceptions pre- and post- delivering telephone-based exercise therapy | Qualitative study nested within an RCT  6-month intervention  Pre- and post-intervention assessment | Physiotherapists (n = 8), 4F (50%), mean clinical experience, yrs 14 (SD 8) |  |  | PT perception, pre- and post-intervention semi-structured interviews |
| Lawford et al. [59]  2020  Australia | To explore therapeutic alliance between KOA patients and physical therapists during a telephone-delivered exercise advice and support | Secondary analysis from an RCT  6-month intervention  Assessment approximately at week 4 and 26 | KOA (n = 84), mean age 62.3 (SD 9.3), 54F (64%)  Physical therapist (n = 8), mean age 35.4 (SD 8.2), 4F (50%), mean clinical experience yrs 13.8 (SD 8.2) |  |  | Primary: Therapeutic alliance, WAI-SF |
| Lawford et al. [60]  2021  Australia | To investigate the associations between KOA patient and physical therapist therapeutic alliance with clinical outcomes (pain, function, kinesiophobia, QoL, PA, adherence and satisfaction) at 6 and 12-month following telephone-delivered exercise therapy | Secondary analysis from an RCT  6-month intervention  Assessments at baseline, 6, and 12 months (also week 4 and 26) | KOA (n = 87), mean age 62.4 (SD 9.1), 55F (63%)  Physical therapist (n = 8), mean age 35.4 (SD 8.2), 4F (50%), mean clinical experience, yrs 13.8 (SD 8.2) |  |  | Average knee pain, NRS; physical functioning, WOMAC; fear of movement; brief fear of movement scale; self-efficacy for pain and function, ASES; HRQoL, AQoL-8D; global changes (overall, pain, function and PA), 7-point scales; satisfaction with care, 7-point scale; physical therapist-rated patient adherence to home exercise, 11-point scale; self-rated adherence, 11-point scale; therapeutic alliance WAI-SF |
| Hinman et al. [61]  2022  Australia | To determine the benefits of adding ‘My Exercise Messages’ to two physiotherapist consultations delivered via videoconference for prescription of a home-based strengthening programme on physical function and exercise adherence | Protocol of a 3-arm pragmatic superiority RCT only  26-week intervention  Assessments at baseline, 14 weeks, and 26 weeks | KOA  Target (n = 182) | MappKO – “My Exercise Messages” app  A 26-week home-based strengthening exercise programme consisting of two video consultations (over two weeks) and use of the ‘My Exercise Messages’ app for 24 weeks  Videoconferencing consultations, hard copy educational resources, exercise equipment, and app  (Comparator = exercise only) | Physiotherapist  2 x videoconferencing consultations (30 mins each) | Primary: exercise adherence, number of days strengthening exercises performed over the past two weeks; physical function, WOMAC  Secondary: knee pain during walking, NRS; sport and recreation function, KOOS; QoL, KOOS; PA, PASE; self-efficacy for exercise, Self-Efficacy for Exercise Scale; perceived global changes in pain function and overall, 7-point Likert; satisfaction with programme and app, 7-point Likert; exercise adherence, EARS; willingness to undergo knee joint replacement, 5-point Likert |
| Hoving et al. [62]  2014  The Netherlands | To evaluate the feasibility of an e-health intervention for employees living with RA that experience work functioning challenges | Feasibility study  3-month e-health programme  Assessments at baseline and 3-months | RA (n = 23), mean age 48 SD 10), 18F (78%), mean time since diagnosis, yrs 5 (SD 5) | A 3-month self-management e-health intervention for employees with RA struggling with work function.  Psychoeducation problem solving: Step 1 – problem identification; Step 2 – solution identification; and Step 3 – planning and action | Nurse:  Contact at least every 2 weeks via email or telephone until the programme was complete (motivation, give feedback on assignments, and solve problems)  At least one F2F contact before step 3 (counselling) | Disease severity, DAS; importance of work and self-efficacy for dealing with problems at work, 0-10 scale  Primary feasibility outcome: satisfaction / acceptability / usefulness, follow-up questionnaire; use of website, Google Analytics; work ability, WAI; work function, WLQ; risk fo work disability, RA-WIS  Experiences, semi-structured interviews |
| Hsu et al. [63]  2021  Taiwan | To investigate the effects of a home and telemedicine-based nutrition and resistance exercise intervention on lower-limb function, body composition and blood chemistry obese patients with KOA | Prospective, single-centre, RCT  12-week intervention  Assessments at baseline and 12-weeks | KOA (n = 66)  Diet (n = 21), mean age 66 (SD 3.9), 12F (57.1%)  Exercise (n = 21), mean age 64.2 (SD 4.1), 13F (61.9%)  Diet and Exercise (n = 21), mean age 65.6 (SD 3.9), 15F (71.4%) | A 12-week, home-based individualized nutrition and resistance exercise programme for obese people with KOA, comprising F2F contact and telephone (or app) follow-up.  (Comparator = Diet control and resistance exercise only) | 1 x F2F visit to medical centre (Dietitian and “clinical staff”)  Dietitian: weekly telephone or communication app (e.g., LINE, FaceTime) contact for 12 weeks (i.e., x 12)  Clinical staff: weekly telephone or communication app contact for 12 weeks (i.e., x 12) – Supervision via telemedicine for first 2 weeks | Primary: body composition, BIA for body weight, BMI, body fat percentage and mass, soft lean mass, and lower-limb muscle mass; height, HW-3070; disability, WOMAC  Secondary: total cholesterol, LDL, triglyceride, blood chemistry analysis; lower-limb function, TUG |
| Huang et al. [64]  2018  China | To explore the feasibility and effectiveness of Guangdong Online Hospital for the management of KOA | Protocol of a prospective, parallel RCT only  6-month intervention  Assessments at baseline, 2 weeks, 3 months, and 6 months | KOA | GOHT  A 6-month, online hospital intervention to KOA self-management  Month sessions include encouragement, educational lectures, and medical issues  (Comparator = conventional therapy) | Clinician  Monthly web-based management (20 – 30 mins) – unclear interaction | Primary: feasibility, proportion of patients who meet eligibility but decline, intervention adherence and withdrawal rates; satisfaction, survey  Secondary: Pain, stiffness and function, WOMAC; fatigue, MFI anxiety and depression, HADS; sleep quality, PSQI |
| Jacobs et al. [65]  2021  USA | To adapt a Relaxation Response Resiliency Program (3RP) for live video delivery for people living with KOA, depression, and obesity and to maximize its feasibility, credibility and acceptability | Protocol for a mixed-methods, feasibility pilot  8-week intervention  Pre- and post-intervention | KOA, depression, and obesity | GetActive-OA  A digital mind-body activity programme  Group-based education, core skills (goals, quota-based pacing, mind-body, cognitive behavioural, and resiliency skills), education on website, daily walking or stationary pedalling, and smartphone log  (no comparator) | Clinical psychologist (and clinical psychology fellow):  1 x weekly group video sessions on Zoom for 8 weeks (90 mins) | Feasibility: credibility and expectancy, CEQ-6; programme satisfaction, CSQ-3; feasibility of recruitment, participation rate; program acceptability, attendance at sessions and home practice; missing data, study clinician adherence, adverse events  Pain, NRS; disease status, KOOS; PA, ActiGraph wGT3X-BTLink accelerometer; self-report of disability, PASIPD; anxiety and depression, PROMIS Anxiety and Depression (v1.08a) and b); pain catastrophizing, PCS; self-efficacy, ASES; coping skills, MOCS-A; perception of improvement, MPGIC; inflammation markers, ELISAs; cartilage breakdown and bony remodelling marker, Urinary CTXII  Experiences, exit interviews  Protocol also states: walk time, 40 m Self-Paced Walk Test |
| Mace et al. [66]  2022  USA | To adapt a mind-body activity programme for live video delivery for people living with KOA, depression, and obesity | Mixed-methods study - development and feasibility pilot  8-week intervention  Pre-and post-intervention | KOA, depression, and obesity  Focus group (n = 9)  Pilot (n = 5), mean age 53.2 (SD 6.64), range 49-65, 5F (100%) |  |  |  |
| Jakiela et al. [67]  2023  USA | To investigate whether a physical therapist intervention, delivered remotely, increases the PA of KOA patients over 12 weeks, compared with existing web-based resources (strengthening, PA, and pain management) only | Protocol of a single-centre, 2 parallel arm, RCT only  12-week intervention  Assessments at baseline, 12 weeks, and 24 weeks | KOA | Delaware PEAK  A 12-week remotely delivered physical therapist intervention for people with KOA  Video consultations including assessment, strengthening exercises, PA, education and homework = plus, home exercise  (Comparator = existing web-based resources only) | Physical therapist:  5 x one-to-one video consultations over 12 weeks (45-60 mins) via Zoom | Primary: change in MVPA, ActiGraph GT3x  Secondary: Light PA, steps per day, ActiGraph GT3x; sedentary time, activPAL4 monitor; self-efficacy for exercise, Self-Efficacy for Exercise questionnaire |
| Khan et al. [68]  2020  USA | To explore if a mobile app and telephone coaching, added to usual care, improves QoL in people living with SLE | Randomized controlled pilot study  16-week intervention  Assessments at baseline and 16 weeks, plus PROMs at week 4, 8, 12, and 16 | SLE (n = 50; 23 controls; 27 intervention), median age 43, 44/46F (96%) | A digital therapeutic programme with telephone coaching  Mobile app for tracking, education telehealth coaching sessions  (Comparator = usual control) | Health coach:  1 x introductory telephone session (unclear who delivered)  1 x weekly telehealth coaching sessions (i.e., 15 sessions for 20-30 mins each)  Study team – messaging on app for technology or support | Primary: fatigue, FACIT-F; pain, BPI-SF; QoL, LupusQoL  Secondary: adherence (number of days an observation is logged), session adherence (numbers of coaching calls), and prevalence of symptoms, triggers and interventions, tracking and dashboard information |
| Lee et al. [69]  2023  Republic of Korea | To explore the effects of a home-based lower extremity muscle strengthening exercise programme in older women living with knee osteoarthritis | A single-centre, parallel-group, open-label RCT  8-week intervention  Assessments at baseline and 8 weeks | Older women with KOA (total n = 31), 31F (100%)  Experimental (n = 15), mean age 65.63 (SD 3.70), 15F (100%)  Control (n = 16), mean age 68.27 (SD 4.78), 16F (100%) | A home-based lower extremity muscle strengthening programme using a remote rehabilitation medical device  Device and exercise training, digital wearable device and app, lower extremity exercises, exercise diary, and video call feedback  (Comparator = usual activities without intervention) | Physical therapist:  1 x explanation of device and exercise (unclear if in-person)  1 x video call every other week for 8 weeks (i.e., 4 calls) to provide feedback (unclear if physical therapist) | Primary: lower-extremity strength, FTSST  Secondary: physical function, TUG test and Fall Risk Assessment tool; muscle biomarkers, quadricep muscle activity and SMI; clinical outcomes, blood pressure, VAS, CRP and ESR |
| Luo et al. [70]  2022  China | To evaluate the feasibility and effectiveness of the “Rheumatism Center” app for improving the management of RA | Protocol of an RCT only  6-month intervention  Assessments at baseline, 4, 12, and 24 weeks | RA  Target (n = 60) | “Rheumatism Center” m-Health app  A m-Health intervention for patient self-management and doctor-patient interaction  Monthly disease management content includes education, management column (medication, test reports, adverse events), disease control status, and communication (expression of negative emotions)  (Comparator = standard care) | Doctor:  Patient to consult doctor when there are concerns – in-person or online (interaction unclear) | Qualitative evaluation: experience and acceptability of app and intervention, interview  Primary: feasibility, proportion of eligible patients who refuse to participate, adherence level, and withdrawal rate. Plus satisfaction with app, questions (Y/Maybe/Not)  Secondary: disease activity, SDAI and CDAI and DAS28; physical function, HAQ, self-efficacy with chronic diseases, SECD6  Economic evaluation: human costs such (e.g., health education data preparation) |
| McCurry et all. [71]  2019  USA | To evaluate the treatment efficacy and cost-effectiveness of a telephone-based CBT intervention for older adults with OA and insomnia | Trial design / protocol of an RCT  8-week intervention  Assessments at baseline, 2 months after treatment and 12-month follow-up | Older adults with OA and insomnia | CBT-I (OATS)  An 8-week, cognitive behavioural therapy intervention for insomnia consisting of reading materials, sleep diary, and telephone consultations  (Comparator = education-only control) | Coach (trained MS-level psychologist, PhD nurse, PhD social worker)  6 x telephone consultations (20-30 mins each) over 8 weeks | Primary: insomnia severity, ISI  Secondary: pain intensity, BPI-sf; depression, PHQ-8; fatigue, FFS  Protocol only (clinical improvement): sleep quality, PSQI; sleep hygiene, SHI; willingness to experience insomnia, SPAQ, willingness to experience pain, CPAQ-8; QoL, EQ-5D; disease, WOMAC |
| McCurry et al. [72]  2021  USA | To evaluate the effectiveness of telephone-based CBT for insomnia (CBT-I) in older adults living with OA | RCT  8-week intervention  Assessments at baseline, 2 months after treatment and 12-month follow-up | OA (n = 327), mean age 70.2 (SD 6.8), 244F (75.6%)  CBT-I (n = 163), mean age 70.1 (SD 7.1), 124F (76.1%)  Education-only (n = 164), mean age 70.4 (SD 6.5), 120F (73.2%) |  |  |  |
| Yeung et al. [73]  2022  USA | To investigate the incremental cost-effectiveness of telephone-based CBT for insomnia for insomnia, arthritis, and QoL outcomes | Cost-effectiveness analysis of an RCT  8-week intervention  Assessments at baseline, 2 months after treatment and 12-month follow-up | OA (n = 325)  CBT-I (n = 162)  Education-only (n = 163) |  |  | Health: HRQoL, EQ-5D; disease, WOMAC; insomnia severity, ISI  Cost: intervention costs, healthcare utilization costs, and total healthcare costs  Additional covariates: depression, PHQ-8; pain, BPI-sf; fatigue, FFS |
| Moutzouri et al. [74]  2022  Greece | To compare the efficacy of a 6-week web-based exercise and outdoor PA intervention with an outdoor PA programme only for people living with KOA | Protocol of a parallel group, 2-arm prospective RCT only  6-week web-based intervention (12 weeks overall)  Assessments at baseline, 6 weeks, and 12 weeks | KOA  Target (n = 56) | WB-OPA / ESCAPE Pain  A 6-week, blended web-based exercise and outdoor PA rehabilitation programme  In-person training session, 6-weeks web-based rehab of exercise and advice on outdoor structured PA  (Comparator = usual care control) | Physiotherapist:  3 x in-person sessions  6 x weekly telephone phone supervision for 6 weeks  12 x pre-recorded video exercise sessions over 6 weeks – unclear  6 x pre-recorded advisory video session – no interaction | Primary: physical function, KOOS pain, NRS  Secondary: physical function, 30CTS and TUG; PA, mBQ and UCLA scale and Yamax Sw200 digi-walker pedometer; HRQoL, SF-12; fear of movement, TSK |
| Müskens et al. [75]  2021  The Netherlands | To assess the real-world use of an eHealth platform for disease management and a self-management outpatient clinic in people living with RA | Observational study  July 2014 – October 2019  Assessments at various intervals (analysis = interrupted time series) | RA  Total population (n = 1059), mean age 64 (SD 13), 705F (67%)  Potential user (n = 761), mean age 61 (SD 13), 497F (65%)  Active user (n = 360), mean age 59 (SD 11), 235F (65%)  Self-management clinic (n = 110), mean age 57 (SD 11), 72F (66% | Reumanet Bernhoven eHealth platform  An eHealth platform to aid healthcare professionals and patients monitor and manage rheumatic disease  eHealth platform, education modules, medical data, PROMs, chat function  (self-management clinic [SMOC] an option) | Health professional:  Chat function for messages to a health professional (unclear)  Optional classes on the use of Reumanet Bernhoven  SMOC – trained nurse specialist:  In-person outpatient visit on the use of eHealth platform  1 x yearly consultation with rheumatologist (plus laboratory monitoring if necessary) | Demographics; health-care utilization, number of outpatient clinic visits; disease activity, DAS28; functional capacity, HAQ; HRQoL, SF-36; impact of RA, RAID  Utilization of eHealth platform, groups split by those with an email address (potential) and those who logged on (active). Plus those with regular PROMs |
| Nero et al. [76]  2018  Sweden | To evaluate the effectiveness of a digital exercise-based treatment programme on physical function in people living with KOA | Protocol of a superiority, 2-arm, RCT  6-week introduction, plus continuous programme for 12 months  Assessments at baseline, 3, 6, and 12 months | KOA  Target (n = 270) | Joint Academy (JA)  A digital exercise-based treatment intervention  Telephone meetings with PT, chat function, education, daily exercises  (Comparator = Better management of patients with OsteoArthritis programme [BOA]) | Physiotherapist:  2 x telephone meetings  Continuous chat function for coaching | Primary: change in number of 30CST repetitions  Secondary: knee pain, NRS; HRQoL, EQ-5D-5L; function, KOOS; PA / exercise, Swedish Board of Health and Welfare indicator questions; patient acceptable symptom state, two questions; estimated healthcare costs; productivity loss, presenteeism and absenteeism, WPAI |
| Nordlund et al. [77]  2021  Multiple countries | To evaluate the effect of a SPIN-SELF programme on disease management self-efficacy, patient activation, functional health and social appearance anxiety, compared to usual care, in people living with SSc | Protocol of a pragmatic, 2-arm, parallel partially-nested RCT  3-month intervention  Assessments at baseline, 3 months, and 6 months | SSc  Target (feasibility n = 40; full-scale trial, n = 524) | SPIN-SELF  An online self-management programme with peer facilitation  Online education programme, assignments, group videoconference sessions, sharing of experiences  (Comparator = usual care control) | Peer facilitator (individual with SSc):  8 x group videoconference sessions (60-75 mins) over 12 weeks (weekly for 4 weeks then biweekly) | Satisfaction, CSQ-8 and for feasibility stage, open-ended questions and PEMAT; usage, automated usage logs through platform  Primary: self-efficacy to manage condition at 3 months, SEMCD  Secondary: self-efficacy at 6 months, SEMCD; social appearance anxiety, SAAS; patient activation, PAM-13; health status, PROMIS-29v2 |
| O’Brien et al. [78]  2016  Australia | To explore the effectiveness of a telephone-based weight management and healthy lifestyle intervention on intensity of knee pain in people living with KOA and obesity (or overweight) | Protocol of a parallel group RCT  6-month intervention  Assessments at baseline, 2, 6, 10, 14, 18, 22, and 26 weeks | KOA (overweight or obese) | A 6-month telephone-based weight management support programme  Education and telephone-based health coaching (NSW Get Healthy Information and Coaching Service)  (Comparator = usual care) | 1 x telephone interview with trained interviewer  10 x tailored health coaching calls (10-15 mins) by qualified health coaches (e.g., dietitians, physiologists, and physiotherapist) | Primary: knee pain intensity over the past week, NRS  Secondary: physical function, WOMAC; weight, self-report and objective ISAK procedures; BMI; waist circumference, objective ISAK procedures; QoL, SF-12.v2; global change in symptoms, Global Perceived Effect Scale; emotional distress, DASS-21; sleep quality, PSQI; PA, Active Australia Survey; dietary intake, Short Food Frequency Questionnaire; alcohol consumption, AUDIT; smoking, self-report; pain attitudes, SOPA; fear avoidance, FABQ; healthcare usage |
| O’Brien et al. [79]  2018  Australia | To determine the effectiveness of a telephone-based weight management and healthy lifestyle programme in reducing knee pain intensity for people living with KOA and obesity (or overweight) | A 2-arm pragmatic parallel group RCT  6-month intervention  Assessments at baseline, 2, 6, 10, 14, 18, 22, and 26 weeks | KOA (overweight or obese) (n = 120), mean age 61.6 (SD 12.6), 74F (62%)  Intervention (n = 59 analysed), mean age 63.0 (SD 11.1), 39F (66%)  Control (n = 60), mean age 60.2 (SD 13.9), 35F (58%) |  |  |  |
| O’Brien et al. [80]  2018  Australia | To evaluate the cost-utility and cost-effectiveness of a telephone-based weight management support programme for people living with KOA and obesity (or overweight) | Economic evaluation of a pragmatic parallel group RCT  6-month intervention  Assessments at baseline, 6 weeks, and 26 weeks |  |  |  | Primary: knee pain intensity, NRS  Secondary: disability, WOMAC; weight and height, self-report; BMI  Cost: Intervention costs (e.g., coaching calls); healthcare utilisation costs (e.g., type of healthcare and medication); Absenteeism, self-report sick days |
| Østerås et al. [81]  2014  Norway | To investigate the clinical effectiveness of an exercise programme with telephone follow-up on hand activity performance in people living with hand OA | Protocol of an RCT  12-week intervention  Assessments at baseline, 3 months, and 6 months | Hand OA | A 12-week hand exercise intervention involving F2F group exercise sessions, home-exercise, and telephone follow-up calls  (comparator = usual care) | Occupational therapist:  4 x F2F group exercise session (weeks 1, 2, 3, 8)  8 x telephone call (weeks 4, 5, 6, 7, 9, 10, 11, 12) | Primary: hand activity performance, FIHOA; function, PSFS 11-point NRS  Secondary: hand pain, NRS; hand stiffness, NRS; patient global assessment of disease activity affecting ADLs, NRS; number of responders, OMERACT and OARSI criteria; maximal grip strength, Jamar Dynamometer; hand dexterity, Moberg Pick-up test; thumb web space, Grip size instrument |
| Østerås et al. [82]  2014  Norway |  | RCT  12-week intervention  Assessments at baseline, 3 months, and 6 months | Hand OA (total n = 130), mean age 66, 117F  Intervention (n = 65), mean age 67 (SD 8), 58F (89%), years of OA diagnosis 11 (SD 9)  Control (n = 65), mean age 65 (SD 9), 59F (91%), years of OA diagnosis 12 SD 70 |  |  |  |
| Paolucci et al. [83]  2022  Italy | To explore if mind-body technique telerehabilitation can affect physical and psychological outcomes of people living with FM | Intervention (not controlled)  8-week intervention  Assessments at baseline, 8 weeks (after treatment), and 1-month follow-up | FM (n = 28), mean age 56.61 (SD 8.56), 28F (100%) | An 8-week, group-based, mind-body technique telerehabilitation programme  8 treatment sessions (movement and stretching mind-body exercises, chat-telephone group, and paper support  (no comparator) | Physiotherapist:  Initial online meeting for set-up and overview  8 x 1 hour Zoom sessions over 8 weeks  Physiotherapist, physiatrist and psychologist also available during session for doubts or questions (chat-telephone group) | Pain, NRS; fatigue, FAS; fear avoidance belief, FABQ  Disability, FIQ; impact of health on life, SF-12  Resilience, RSA (perception of self, planned future, social competence, structured style, family cohesion, and social resources) |
| Park et al. [84]  2022  South Korea | To assess the effect of a telepharmacy service on humanistic and clinical outcomes of people living with RA, compared to a usual pharmacist service | Protocol of a prospective, open-label RCT only  6-month intervention  Assessments at baseline, 3 months, and 6 months | RA | PROUD study / MediRA app  A telepharmacy care service for people living with RA  Monthly medication counselling and personalized mobile app for compliance  (Comparator = usual care) | Clinical pharmacists:  Monthly medication counselling  Call or message contact in app for side effects or questions | Primary: HRQoL, Korean EQ-5D  Secondary: patient compliance with anti-rheumatic drug regimen, K-CQR; patient satisfaction with pharmaceutical care services, K-PSQ; medication knowledge, modified brief medication questionnaire; ESR, CRP, pain and joint involvement, chart review; utilisation data of MediRA, number and time of app use; satisfaction with app, 5-point Likert |
| Pasyar et al. [85]  2023  Iran | To evaluate the effectiveness of smartphone-based supportive counselling on acceptance of disability and health anxiety in people living with SLE | RCT (pre- and post-design)  8-week intervention  Assessments at baseline and 8 weeks | SLE (n = 124), 116F  Intervention (n = 62), mean age 44.19 (SD 9.69), 60F (96.8%)  Control (n = 62), mean age 48 (SD 8.92), 56F (90.3%) | A smartphone-based supportive counselling intervention  WhatsApp channel for daily supportive counselling (messages, photos, video and audio). Potential for patients to interact with each other on WhatsApp channel  (Comparator = routine care, no counselling) | Research team (rheumatologist, senior psychologist, and nurse):  Online consultation (unclear)  Unclear interaction – WhatsApp channel for daily supportive counselling. Participants could send comments for inclusion in the following sessions | Health anxiety, Persian version of the Salkowski & Warwick Health Anxiety Questionnaire; acceptance of disability, Persian version of the acceptance disability scale |
| Patel et al. [86]  2022  USA | To evaluate the feasibility and acceptability of tele-EnhanceFitness in rural older adults living with symptomatic KOA | Single-arm pilot  12-week intervention  Assessments at baseline and 12 weeks | Rural, older adults with KOA (n = 15), mean age 71.8 (SD 5.8), 14F (93.3%) | A 12-week remotely delivered (via videoconference) EnhanceFitness exercise programme  36 live sessions, adjustable weights, and social interaction online for 10-mins prior to each session  (no comparator) | EnhanceFitness-certified instructor (and assistant):  3 x groups videoconferencing sessions every week for 12 weeks (i.e., 36 1-hour sessions) – questions at the end of each session  Assistant for helping instructor, troubleshooting, and monitoring safety  (plus one 30-min orientation session on zoom prior to intervention) | Feasibility, proportion eligible, screened, and refused. Attendance, tech problems, and call length. Acceptability, end of trial questions  Experiences, semi-structured exit interviews  Secondary: pain and function, KOOS; HRQoL, PROMIS-29; change in health, single-item PGIC; physical capacity, TUG, SPPB, and single leg stand with open eyes test  Videoconference technology acceptance, TAM scale |
| Paul et al. [87]  2016  Scotland, UK | To explore adherence to, and the effect of, a 12-month web-based intervention for people living with axSpA | Protocol of a prospective, intervention, cohort study  12-month intervention  Assessments baseline, 6 months, and 12 months | axSpA | WEBPASS  A 12-month, individualized, online physiotherapy programme  Initial agreement of goals and exercise prescription, exercise dairy, online exercise platform, telephone calls  (no comparator) | Physiotherapist:  1 x initial F2F visit to agree exercise goals and prescribe individual programme  2 x telephone calls over the first two weeks to check progress and answer questions  Telephone contact to answer questions (not pre-defined) | Primary: adherence to the online programme, online exercise diary  Secondary: exercise capacity, 6MWT; function, BASFI; disease activity, BASDAI; spinal mobility, BASMI; QoL, ASQoL; health status, EQ-5D; work impairment, presenteeism and absenteeism, WPAI; how attitude affects compliance to exercise, EAQ; motivation to exercise, EMI-2; PA, activPAL accelerometer  Experiences, semi-structured interviews |
| Paul et al. [88]  2023  Scotland, UK | To investigate the adherence to, view of, and effect of, a 12-month, online physiotherapy programme for people living with axSpA | Prospective, intervention, cohort study  12-month intervention  Assessments at baseline, 6 months, and 12 months | axSpA (n = 50), mean age 50 (SD 11.7), 27F (54%), mean duration since diagnosis, yrs 16.2 (SD 11.9) |  |  |  |
| Pedley et al. [89]  2021  UK | To explore the feasibility, acceptability and change in health outcomes following a telephone-based CBT (tCBT) intervention for people living with axSpA, with or without co-morbid FM | Feasibility and acceptability study  6-week intervention  Assessments pre- and post-intervention | axSpA (n = 28) median (IQR) age 59 (49, 70), 11F (40.7%), median (IQR) years from symptom onset 36 (12, 48) | A 6-week telephone-based CBT (coaching) intervention with CBT manual  List of behaviour change techniques provided  (no comparator) | Initial assessment (45-60 minutes)  Psychological well-being practitioner:  6 x weekly one-to one sessions (30 mins each) | Patient: HRQoL, ASQoL; mental health, HADS; fatigue, Chalder Fatigue Scale; sleep disturbance, Jenkins Sleep Evaluation Questionnaire; spinal pain, VAS disease activity, BASDAI; function, BASFI; widespread pain and symptom severity, 2011 research criteria for FM; PA, walking / moderate / vigorous PA per week questions; change in health, single questions  Acceptability and experiences: Interviews |
| Piga et al. [90]  2014  Italy | To describe the feasibility of telemedicine applied to kinesiotherapy for hand function in people living with SSc and RA | Pilot RCT  12-week intervention  Assessments at baseline, week 6 and week 12 | SSc and RA (n = 40)  Intervention: SSc (n = 10), mean age 57.0 (SD 10.0), 10F (100%), mean disease duration 6.9 (SD 4.1); RA (n = 10), mean age 56.3 (SD 10.3), 9F (90%), mean disease duration 13.1 (SD 10.0)  Control: SSc (n = 10), mean age 57.4 (SD 11.7), 10F (100%), mean disease duration 6.7 (SD 4.2); RA (n = 10), mean age 56.8 (SD 12.3), 9F (90%), mean disease duration 12.8 (SD 10.5) | Re.Mo.Te  A telerehabilitation programme consisting of kinesiotherapy for hand dysfunction  Home rehabilitation exercises, store-and-forward, remote monitoring  (Comparator = home kinesiotherapy, aided by common daily-life objects) | Physician / investigator:  1 x in-person training session (1 hr) by investigator  Telephone contact based on loss of sessions or worsening exercise trends – feedback on modifying protocol  Remote monitoring by physician | Primary: hand function, HAQ, FIHOA, and HAMIS  Secondary: maximum hand abduction and joint ROM, goniometer; grip strength and pinch strength, sphygmomanometer; QoL, SF-36, VAS pain, and VAS-GH; compliance, number of participants completing 12 weeks  Satisfaction, QUEST 2.0 |
| Pani et al. [91]  2017  Italy | To explore the experiences and perspectives of people living with RA and SSc on the use of a telerehabilitation system for hand dysfunction | Pilot RCT  12-week intervention  Assessments after the trial and one-year post-trial interviews |  |  |  | Impact on QoL of using technology, PIADS; satisfaction, QUEST; effectiveness of technology in regard to patient relevant activities, IPPA  Experiences, semi-structured interview  Hand function, HAQ and FIHOA |
| Raad et al. [92]  2022  Ireland | To determine the effect of a Mediterranean diet compared to a standard healthy diet on QoL and physical function in people living with RA | Protocol of a parallel group RCT  12-week intervention  Assessments at baseline and weeks 3, 6, 9, and 12 | RA | MEDRA  A 12-week telehealth dietary intervention comparing a Mediterranean diet with a standard health diet  (plus, MedDiet guide and Libro mobile application)  (comparator = standard healthy diet defined by Healthy Eating Guidelines [HEG]) | Dietitian:  3 x video teleconsultations over 12 weeks (30-45 mins on weeks 0, 6, and 12) – dietary education and monitoring  2 x review telephone call reviews (10-20 mins on weeks 3 and 9) – monitoring and goal setting | Primary: QoL, RAQoL; change in physical function, HAQ-DI  Diet and PA: food and exercise diary, Libro app; change in PA, YPAS  Anthropometric: height, portable height board; weight, scales |
| Raad et al. [93]  2023  Ireland | To explore RA patients’ experience of a 12-week telehealth Mediterranean dietary intervention and the acceptability of the intervention | Qualitative analysis for an RCT  12-week intervention  Focus groups after intervention completion (12 weeks) | RA (n = 21), mean age 47.5 (SD 12.3), 20F (95.2%)  MedDiet (n = 11), mean age 47.5 (SD 14.5), 11F (100%)  HEG (n = 10), mean age 47.3 (SD 10.2), 9F (90%) |  |  | Experiences: semi-structured focus groups (also described in protocol) |
| Raunsbæk Knudsen et al. [94]  2021  Denmark | To evaluate the effectiveness of an e-learning education programme for improving self-management of RA | Protocol for a pragmatic multi-centre RCT only  Module 1 within 4 weeks, and module 2 and 3 within study period  Assessments at baseline and after 1, 3, 6, and 12 months of follow-up | RA  Target (n = 190) | WebRA study  An e-learning education programme targeting self-management  Web-based patient education and telephone consultation  (Comparator = in-person patient education) | Nurse:  1 x information on disease management as part of enrolment (usual care)  1 x telephone consultation about methotrexate treatment | Primary: self-efficacy, RASE  Secondary: knowledge of RA, PKQ; adherence to medication, CQR5; health literacy, HLQ; QoL, EQ-5D-5L  Clinical: disease activity, DAS28 and CDAI; functional status, MDHAQ  Plus healthcare utilization and utilisation of e-learning programme |
| Rodríguez Sánchez-Laulhé et al. [95]  2023  Spain | To investigate the effects of a mobile app-delivered intervention, compared to usual care, on hand function and pain in people living with hand OA | Pragmatic, multi-centre, 2-group parallel RCT  12-week intervention  Assessments at baseline and 1-, 3-, and 6-month post-intervention | Symptomatic hand OA (n = 74), 50F (67.5%)  CareHand (n = 34), mean age 62.2 (SD 8.8), 25F (73%)  Usual care (n = 40), mean age 64.3 (SD 7.7), 25F (62%) | CareHand app  A home-based, hand exercise programme, delivered via a mobile application  Introductory session, Education and self-management strategies, hand exercises 4x per week (15-20 mins) for 12 weeks, daily pain reporting for dosing algorithm and progress, monthly follow-up calls  (Comparator = usual care – paper sheets) | Physiotherapist researcher:  1 x in-person introductory session - code access, app explanation treatment explanation  Research team, physiotherapists, and primary care physicians:  Monthly telephone calls - monitoring compliance and progress, and to answering queries | Primary: hand function, subscale of the AUSCAN  Secondary: overall hand status, AUSCAN total and QuickDASH; pain intensity, AUSCAN subscale and NRS; morning stiffness, AUSCAN subscale and NRS; upper limb function, NHPT; grip and pinch strength, dynamometer and mechanical pinch gauge |
| Rodríguez Sánchez-Laulhé et al. [96]  2020  Spain | To explore the effects of a mobile app-delivered programme, compared to usual care, on hand function, pain, stiffness, and pinch/grip strength in people living with hand RA | Protocol of a single-blinded, superiority, 2-arm, parallel RCT  12-week intervention  Assessments at baseline, 1 month and 3 months from baseline | Hand RA  Target (n = 58) | CareHand app  A home-based, hand exercise programme delivered by a mobile application  Introductory explanatory session with motivational messages, self-management strategies for pain, fatigue and diet, 4 x home-based hand exercises per week (15-20 mins), monthly follow-up calls  (Comparator = usual care – paper sheets) |  | Primary: overall hand function, MHQ  Secondary: functional ability, QuickDASH; pain intensity, VAS; morning stiffness, VAS; hand grip and strength, dynamometer and Pinch Gauge |
| Rodríguez Sánchez-Laulhé et al. [97]  2022  Spain | To investigate the effects of a mobile app-delivered home therapeutic hand exercise programme, compared to usual care, in people living with hand RA | Single-blinded, superiority, 2-arm, parallel RCT  12-week intervention  Assessments at baseline, 1-, 3-, and 6-months follow-up | Hand RA (n = 36), age range 43 – 78; 22F (61%)  CareHand (n = 14), mean age 57.64 (SD 7.25), 9F (64%)  Usual care (n = 22), mean age 61.86 (SD 10.76), 13F (59%) |  |  |  |
| Smarr et al. [98]  2011  USA | To describe an online, cognitive behavioural, self-management programme for people living with RA | Randomized prospective trial  10-week intervention on average at time of analysis | RA (n = 114; utilisation and management data analysed for 33 participants) | RAHelp  A web-based, cognitive behavioural, self-management programme with telephone support  Education, psychoeducational resources, telephone support and peer support  (comparator = no information) | Trained clinician (leaders)  1 x weekly telephone contact (for an average of 10 weeks)  Messaging system for between member contact and to ask leaders questions | Evaluation, usage and interaction in 33 members and one leader surveyed  Online activity, number of clicks, messages, and discussion boards etc  Administrative burden: monitoring of site, time for phone contact, and email and qualitative review  Member evaluation, reported elsewhere |
| Shigaki et al. [99]  2013  USA | To evaluate the effect of an online, cognitive behavioural, self-management programme for improving self-management in people living with RA | A 2-group RCT  10-week intervention  Assessments at baseline, post-intervention and 9-month follow-up | RA (n = 106)  Intervention (n = 54), mean age 50.3 (SD 11.6), 50F (93%), mean duration of RA 7.4 (SD 8.6)  Control (n = 52), mean age 49.3 (SD 12.3), 48F (92%), mean duration of RA 8.5 (SD 10.3) | RAHelp  A web-based, cognitive behavioural, self-management programme with telephone support  Education, psychoeducational resources, telephone support and peer support via messaging system  (Comparator = waiting list control) | Clinician leader (masters-prepared counsellor):  1 x weekly one-to-one telephone calls for 10 weeks (15-30 mins) | Health status and well-being, AIMS2; self-efficacy for arthritis, ASES; depression, CES-D; QoL, QLS; disease activity, RADAR; perceived social functions, SPS; loneliness, LS-3 |
| Singh et al. [100]  2022  India | To explore the feasibility and effectiveness of an e-health yoga intervention for people living with AS during COVID-19 | Protocol for a single-centre, parallel-group prospective, open-blinded end-point trial only  3-month intervention  Assessments at baseline and 3 months | AS  Target (n = 135) | A 3-month tele yoga intervention amidst the COVID-19 pandemic  Online yoga sessions and home yoga encouraged.  (Comparator = standard care control) | Yoga therapist:  4 x a week online yoga sessions for 3 months (60 mins each)  Potential bi-weekly calls over non-contact period (unclear) | Primary: disease activity, BASDAI; function, BASFI; QoL, ASQoL; inflammation, CRP and ESR  Secondary: depression and anxiety, PHQ-4 |
| Singh et al. [101]  2023  India | To investigate the effectiveness of tele yoga on disease activity, quality of life, and inflammatory markers in people living with AS | A single-centre, parallel-group, prospective RCT  3-month intervention  Assessments at baseline and 3 months | AS (total n = 109) mean age 34.74 (SD 9.57), 18F (16.5%), disease duration N (%) 3 years 13 (12.3%), 3-5 years 14 (13.2%), 5-10 years 42 (39.6%), >10 years 37 (34.9%)  Yoga (total n = 57) mean age 34.42 (SD 9.39), 9F (15.8%), disease duration N (%) 3 years 7 (12.7%), 3-5 years 5 (9.1%), 5-10 years 22 (40%), >10 years 21 (38.2%)  Control (total n = 52) mean age 35.09 (SD 9.86), 9F (17.3%), disease duration N (%) 3 years 6 (11.9%), 3-5 years 9 (17.6%), 5-10 years 20 (39.2%), >10 years 16 (31.3%) |  | Yoga therapist:  2 x yoga sessions per week for 3 months (60 mins each)  Unclear if telephone calls were performed during non-contact as mentioned in protocol |  |
| Song et al. [102]  2020  China | To investigate the effect of a tailor telehealth education intervention on disease activity and medication adherence in people living with RA | RCT  12-week intervention  Assessments at baseline, 12 weeks, and 24 weeks | RA (total n = 77) mean age = 55.26 (SD 10.84), 55F (71.4%), median (IQR) disease duration 4.50 (8.75)  Intervention (n = 41) mean age 57.05 (SD 11.31), 30F (73.2%), median (IQR) disease duration 4.00 (9.00)  Control (n = 36), mean age 53.22 (10.0), 25F 69.4%, median (IQR) disease duration 4.50 (8.40) | A 12-week intervention comprised of four tailored, one-to-one, telephone-based, educational sessions after hospital discharge  (Comparator = usual care) | Research Nurse  4 x telephone educational sessions from research nurse (20 – 40 minutes each) | Medication adherence, Chinese version of CQR; disease activity, ESR, CRP and DAS28 |
| Song et al. [103]  2022  China | To investigate the effect of a WeChat-based educational programme on self-efficacy, disease knowledge, exercise adherence and health outcomes in people living with AS | Single-blind, parallel-group RCT  12-week intervention  Assessments at baseline and 12 weeks | AS (n = 118), mean age 29.93 (SD 8.23), 25F (21.2%), median (IQR) symptom duration, yrs 5.00 (6.00), median (IQR) duration since diagnosis, yrs 3.00 (6.00)  Intervention (n = 59) mean age 30.80 (SD 8.82), 14F (23.7%), median (IQR) symptom duration, yrs 6.00 (7.00), median (IQR) duration since diagnosis, yrs 3.00 (6.00)  Control (n = 59) mean age 29.07 (SD 7.58), 11F (18.6%), median (IQR) symptom duration, yrs 5.00 (7.00), median (IQR) duration since diagnosis, yrs 3.00 (6.00) | WeChat based  An educational programme delivered via WeChat  Education, live education sessions, and encouragement to share experiences  (Comparator = standard care control) | Nurses  4 x individual educational sessions (20-30 mins) through voice or video over 12 weeks  “Chat” with nurses at any time | Primary: disease knowledge, Assessment of Knowledge in Ankylosing Spondylitis Patients; self-efficacy, ASES-8; exercise adherence, self-reported statement  Secondary: disease activity, BASDAI; physical function, BASFI |
| Song et al. [104]  2021  China | To investigate the effect of a WeChat-based educational programme on HRQoL, depression, and other outcomes in people living with AS |  |  |  |  | HRQoL, SF-36; depression, BDI-II; effect of AS on well-being, BAS-G; physical function, BASFI; overall morning stiffness and pain, VAS |
| Song et al. [105]  2022  China | To investigate the effect of a physiotherapist-delivered eHealth education tool on perception of KOA and non-pharmacological treatment options, satisfaction, and compliance | Protocol of a prospective RCT only  6-month intervention period (consultation within first 7 days)  Assessments at baseline, post-consultation, and 1-, 3-, and 6-month follow-up | KOA  Target (n = 216) | WeChat based  A physiotherapist-delivered eHealth education tool  Video consultation, education centre and automatic push notifications within WeChat Messenger  (Comparator = usual care) | Physiotherapist:  1 x videoconferencing consultation (90 mins) | Primary: patient knowledge of OA, PKQ  Secondary: satisfaction with consultation, education tool, and PT trust, 6 closed and 2 open questions  Additional: usability, 13 closed and 3 open questions and web-based analytics; perspectives, semi-structured interviews |
| Stanton et al. [106]  2020  Australia | To evaluate the feasibility of adding pain science education, to a physiotherapist-led education and walking programme for people living with KOA | Randomized, parallel-group, feasibility trial  8-week intervention  Assessments at baseline, 4, 8, and 26 weeks | KOA (n = 20), mean age 67.0 (SD 7.4), 14F (70%)  PSE (n = 10), mean age 69.2 (SD 6.5), 6F (60%)  Control (n = 10), mean age 64.8 (SD 7.9), 8F (80%) | A physiotherapist-delivered pain education and walking programme  One-on-one in-person sessions with a PT, phone calls with PT, pain science education, and walking programme  (Comparator standard education plus sham ultrasound) | Physiotherapist:  4 x weekly in-person treatments  Followed by 4 x weekly telephone calls | Primary: feasibility, recruitment and eligibility, intervention adherence, compliance with PA assessment, and retention at long-term follow-up assessment; intervention acceptability, participants’ and clinicians’ perspectives, PEQ, clinician questions  Secondary: barriers to participation and change in clinical and PA outcomes  Outcomes: PA, IPAQ-SF and Actigraph GT9X; pain, NRS and VAS, pain beliefs, PBQ, pain self-efficacy, PSEQ; fear of movement, Brief fear of movement scale for OA; pain catastrophizing, PCS; function, PSFS, pain knowledge, rNPQ, pain, function, total, WOMAC |
| Stanton et al. [107]  2021  Australia | To investigate the clinical- and cost-effectiveness of physiotherapist-delivered pain science education and an individualised walking, strengthening and education programme for people living with KOA | Protocol of a 2-arm, multicentre, superiority RCT only  9-month intervention  Assessments at baseline, 12 weeks, 6 months, and 12 months | KOA not meeting PA guidelines  Target (n = 198) | EPIPHA-KNEE trail  A physiotherapist-delivered pain science education and walking / strengthening programme  11 consultations, pains science education, graded walking programme, and strengthening exercise programme  (Comparator = best practice education / active control) | Physiotherapist:  4 x in-person sessions for 4 weeks (60-90 mins)  Followed by 4 x telephone or videoconference sessions for 4 weeks (20 mins)  1 x follow-up telehealth session at 3 months (20 mins)  2 x in-person follow-up at 5 and 9 months (45-60 mins) | Primary: average step count over 7 days, Actigraph GT9X Link; overall knee symptoms, WOMAC  Secondary: average knee pain, NRS; average knee pain when walking, NRS; knee pain and function over 48 hours, WOMAC; depression, PROMIS subscale, anxiety, PROMIS subscale; stress, Perceived Stress Scale; fear of movement, Brief Fear of Movement for OA scale; pain catastrophizing, PCS; HRQoL, EQ-5D-5L; knee perception, FREKAQ; change in knee, pain and function, 7-point Likert; sedentary, light, moderate and vigorous PA, Actigraph GT9X Link  Plus: conceptualization of OA and PA, OACS; pain self-efficacy, PSEQ and cost and adherence measures |
| Tahran et al. [108]  2023  Turkey | To investigate the feasibility and preliminary effect of internet-based basic body awareness therapy (BBAT) on clinicals outcomes in people living with FM | Case Reports  8-week intervention  Assessment pre- and post-intervention | FM (n = 3)  1 x 40-year-old female  1 x 45-year-old female  1 x 43-year-old male | Individual, online videoconference based BBAT over 8 weeks  60-minute sessions comprising exercises (balance, posture, breathing and coordination) and experience sharing  (no comparator) | Physiotherapist:  2 x weekly online videoconference sessions (60 min) for 8 weeks | Functional disability, FIQR; body awareness, ABC; quality and intensity of pain, MPQ-SF; level of dysautonomia, plasma fibrinogen level |
| Tam et al. [109]  2019  Canada | To determine whether an e-health, disease monitoring and PA counselling intervention improves self-management ability in people living with RA | Protocol of a delayed control RCT only  6-month intervention  Assessments at baseline, week 27, and week 53 | RA | OPERAS app  A 6-month, e-health intervention comprised of education, monitoring and counselling  Group education session, Fitbit Inspire, individual counselling, disease monitoring, telephone coaching  (Comparator = delayed control) | Physiotherapist:  1 x in-person session (inc. group education and individual counselling) – 2 hour  6 x telephone calls (weeks 2, 4, 5, 8, 13, and 26) | Primary: self-management ability, PAM  Secondary: disease status, RADAI; pain, MPQ-SF; fatigue, FSS; mood, PHQ-9; habitual behaviour, SRHI; PA, MVPA and sedentary activity, SenseWear Mini |
| Tore et al. [110]  2023  Turkey | To explore the effects of telerehabilitation with a physiotherapist, compared to a home-based exercise programme, in people living with knee osteoarthritis | RCT  8-week intervention  Assessments at baseline and 8 weeks | KOA (total n = 48), mean age 55.83 (SD 6.93), 43F (89.6%)  Telerehabilitation (n = 24), mean age 55.87 (SD 7.24), 21F (87.5%), mean disease duration, years 5.72 (SD 3.49)  Control (n = 24), mean age 55.79 (SD 6.76), 22F (91.7%), mean disease duration, years 7.87 (SD 4.16) | A telerehabilitation exercise programme, delivered via videoconference, with a physiotherapist.  Education and then telerehabilitation 3 x a week for 8 weeks  (Comparator = brochure explaining how to do exercises only) | Physiotherapist:  1 x education session  3 x telerehabilitation per week for 8 weeks (i.e., 24 sessions at 45-60 mins each) | Lower extremity muscle strength and balance, 30CST; PA readiness, Physical Activity Readiness Questionnaire for Everyone; physical function, KOOS; pain severity, NRS; PA, IPAQ-SF; depression and anxiety, HADS; quality of treatment, QUIPA; fear of movement, TSK; adherence to recommended exercises, EARS; fatigue, Fatigue Severity Scale; satisfaction with treatment, 5-point Likert |
| Vallejo et al. [111]  2015  Spain | To compare the efficacy of an internet-delivered CBT programme with a group-based in-person CBT programme for people living with FM | 3-arm, parallel RCT  10-week intervention  Assessments at baseline, post-intervention, 3, 6, and 12-month follow-up | FM (n = 60), mean age 51.55 (SD 9.87), 60F (100%)  iCBT (n = 20), mean age 49.82 (SD 11.01), 20F (100%)  CBT (n = 20), mean age 53.50 (SD 8.56), 20F (100%)  Waiting list (n = 20), mean age 51.33 (SD 10.03), 20F (100%) | An individual internet-delivered, 10-week, CBT programme  Access to weekly materials, mp3 file, activities and therapist questions  (comparator = waiting list control and group-based in-person CBT) | Junior therapist  Messages to therapist – questions at the end of each session, tracking, feedback | Primary: global impact (pain, function, sleep, fatigue, and mental health), FIQ  Secondary: psychological distress, HADS; depression, BDI; catastrophizing, PCS; self-efficacy, CPSS; coping, CPCI |
| Walter et al. [112]  2023  Sweden | To investigate the effects of a 3-month digital first-line treatment on pain and perceived hand function in people living with hand OA | Observational registry-based study  3-month intervention  Assessments at baseline and 3 months | Hand OA (n = 379), mean age 64.6 (SD 9.3), 285F (75.2%) | Joint Academy  A digital exercise-based intervention for hand OA  Daily video exercises, text education, optional social chat, and teleconsultations with PT  (no comparator) | Physiotherapist:  3 x teleconsultations (telephone or video)  Plus, continuous asynchronous chat | Primary: pain, NRS  Secondary: hand stiffness, NRS; activity performance, FIHOA; medication use, kinesiophobia, willingness to undergo surgery, injection in the index joint, dichotomous questions |
| Wang et al. [113]  2021  China | To explore the effects of a 12-week education and physiotherapist-supervised progressive exercise programme on clinical outcomes in older people living with KOA | Protocol of a 2-arm, randomized, positive-controlled, prospective and longitudinal study (RCT)  12-week intervention  Assessments at baseline, 4, 8, 12, 16, 20, and 24 weeks | Older KOA | Mobile online platform – Joint Consultation  A 12-week intervention comprised of progressive exercise supervised by a PT and needs-based education via video link  Education, PT supervision, home-exercise with family members accompanying  (Comparator – neuromuscular vs quadriceps exercise) | Physiotherapist:  1 x weekly exercise session via video link for 12 weeks (i.e., 12 sessions)  1 x monthly needs-based education sessions via video link (4 sessions in total, lasting 15-20 mins each) | Primary: pain during walking, NRS; function, WOMAC  Secondary: pain over 48 hours, WOMAC subscale; irratibility with pain pressure threshold, pressure algometer (Wagner Force Ten, FDX 25); exercise sensitisation, NRS; function, TUG and 6MWT; pain catastrophizing, PCS; fear of movement, TSK; self-efficacy for exercise, Self-efficacy for Exercise Scale; pain self-efficacy, PSEQ; resilience, CD-RISC-10; acquisition of knowledge of KOA, ENAT; social support, SRSS; social participation, WHYMPI; QoL, SF-36 |
| Wang et al. [114]  2022  China | To compare the effects of IoT-based power cycling and quadriceps with a control group on pain, function, QoL and adherence in people living with KOA | Protocol of a 3-arm, single-blinded parallel RCT only  12-week intervention  Assessments at baseline, 4, 8, and 12 weeks | KOA  Target (n = 72) | Internet-of-Things (IoT) – WeChat applet “Huaxi Cloud Rehabilitation” (HXCR)  An online-based education and power cycling or quadricep exercise intervention  Education and questionnaires pushed on HXCR by doctors and PTs. Prescribed exercise and video link with PT.  (Comparator = IoT power cycling vs internet-based quadriceps vs internet-based control) | Physiotherapist:  Pushed education and exercise prescription (PT and doctor – no interaction)  1 x weekly video link for 12 weeks (i.e., video 12 calls)  Telephone or video contact participant if not completing exercises  (Power cycling at a community activity centre power cycling park and family member available during exercise) | Primary: pain, WOMAC  Secondary: knee pain in previous month, NRS; HRQoL, SF-36; physical function, WOMAC physical performance, TUG; adherence, ratio of completed HXCR sessions to total sessions |
| Warmington et al. [115]  2017  Canada | To explore the feasibility and IA patient satisfaction with a telemedicine delivered “Prescription for Education” (RxEd) programme | Feasibility (mixed methods approach)  1-day intervention  Post-course programme evaluation and follow-up interview | IA (n = 123), 98F  Remote (n = 87), mean age 58.58 (SD 13.36), 76F (87.4%), mean disease duration, yrs 7.92 (SD 11.15)  In-person (n = 36), mean age 56.80 (SD 13.09), 22F (91.7% - unclear), mean disease duration, yrs 11.57 (SD 13.59) | RxEd  A 1-day, multi-point videoconferencing, IA education programme  Remote education delivered via videoconference to 6 rural locations with a local Advanced Clinician Practitioner in Arthritis Care programme-trained practitioners | Interprofessional arthritis care team (educators):  Delivered 1-day RxEd  Physiotherapist or occupational therapist:  Led in-person facilitation and site activities, answered questions and managed videoconference tech | Perception and evaluation, participant evaluation forms; disease activity, RADAI  Experience, semi-structured follow-up interviews  (Plus educators post course feedback, feedback form) |
| Kennedy et al. [116]  2017  Canada | To compare the in-person and telemedicine RxEd programmes in terms of improving IA patient arthrits self-efficacy and other outcomes | Non-randomized, pre-post design  1-day intervention  Assessments at baseline, immediate post-treatment, and 6-month follow-up |  |  |  | Primary: self-efficacy, 8-item scale (unclear)  Secondary: knowledge about arthritis, ACREU Rheumatoid Arthritis Questionnaire; coping efficacy, 4-item scale (unclear); intrusiveness of illness, IIRS; effective consumer, Effective Musculoskeletal Consumer Scale  Disease activity, RADAI; disability, HAQ |
| Weber et al. [117]  2023  Germany | To evaluate the effectiveness of a blended care intervention on physical function and pain, in people living with knee and/or hip OA, compared to usual care | Protocol of a three-centre, pragmatic, parallel group, RCT  12-month intervention  Assessments at baseline, 3- and 12-months | Hip and Knee OA  Target (n = 330) | SmArt-E intervention  A blended intervention comprising a neuromuscular exercise programme,  disease-specific education, and promotion of graded or non-graded daily PA  In-person sessions with a physiotherapist (individual or group-based) and app-supported training and education  (Comparator = usual care) | Physiotherapist:  2 x group sessions every week for 6 weeks (i.e., 12 sessions at 80 mins each) OR 4-8 individual sessions over 12 weeks (40 mins each)  1 – 3 individual refresher sessions (40 mins each) at the 6-month period  Chat and videoconference feature within the app for PT interaction | Primary: physical function, HOOS or KOOS; pain and tiredness, NRS  Secondary: physical capacity, Modified Y-Balance-Test and 30CST; PA, ActiGraph GT3X and activity questionnaire; action planning and coping planning, questionnaire; HRQoL and health status, EQ-5D-5L, PGA, and subjective change in health status; pain catastrophizing, PCS; psychological status, SRBAI, PASS and ASES; satisfaction, ZUF-8 plus 1 item, health literacy, Movement-related Health Literacy  Other: usability, SUS and MAUQ; health literacy, HLS-EU-Q16 and eHEALS; technology readiness, technology commitment; health economic evaluation, expenses and resource use; health insurance routine data and user-intervention interaction |
| Xie et al. [118]  2020  China | To explore the feasibility and effectiveness of internet-based rehabilitation programme with community exercise and wearable technology for people living with KOA | Protocol of a 12-week RCT only  12 -week intervention  Assessments at baseline, 6 weeks, and 12 weeks | KOA | ‘FOR KOA’ software  An internet-based education, self-management, and land-based exercise programme  Online education, self-management (emotional, weight and medication), plus exercise and exercise prescription (plus bracelet)  (Comparator = control) | Physiotherapists:  Communication through application (leave messages)  First assessment (physician and physiotherapist) – remote guidance  Psychotherapist – voice or video via application (unclear interaction)  FOR KOA support – voice, video, or in-person with research staff | Primary: feasibility, data completeness, willingness to participation, satisfaction with programme, and compliance; satisfaction, survey  Secondary: knee pain, NRS; pain, stiffness, and function, WOMAC; QoL, SF-36; adverse events; demographics including PA, IPAQ-SF |
| Zhang et al. [119]  2022  China | To compare the effects of IoT-based power cycling and neuromuscular exercise on pain and walking in elderly people living with KOA | Protocol of a 12-week single-blind RCT only  12-week intervention  Assessments at baseline, 4, 8, and 12 weeks | Elderly KOA  Target (n = 42) | Internet-of-Things (IoT) – WeChat applet “Huaxi Cloud Rehabilitation” (HXCR)  An online-based education and power cycling or neuromuscular exercise intervention  Education, self-management (weight and emotional), prescribed exercise and video link with PT.  (Comparator = power cycling vs neuromuscular vs control) | Physiotherapist:  1 x weekly video call for 12 weeks  Doctors and physiotherapists perform telerehabilitation assessments  Doctors and physiotherapists push information 3 x per week | Primary: pain, stiffness, function, WOMAC; pain in last 48hr, NRS  Secondary: lower limb strength and balance, TUG; 6MWT; QoL, SF-36 |
| Zhao & Chen [120]  2019  China | To investigate the effectiveness of a health education programme delivered by telephone follow-up on the self-efficacy of discharged RA patients | RCT  12-week intervention  Assessments at baseline (day before discharge), 12, and 24 weeks after discharge | RA (n = 92), mean age 55.5 (SD 10.6), 66F (71.7%), disease duration, yr 4 (0.73, 10.00)  Control (n = 46), mean age 54.15 (SD 10.06), 32F (69.6%), disease duration, yr 4.25 (0.27, 9.62)  Intervention (n = 46), mean age 56.93 (SD 11.14), 34F (73.9%), disease duration, yrs 4 (1.32, 10.50) | A health education programme by telephone follow-up after discharge  Education regarding medication, diet, and exercise skill via telephone  (Comparator = control group) | Specialist rheumatology nurse:  4 x health education by telephone follow-up over 12 weeks (i.e., 4 total calls) | Primary: disease activity, DAS28 (inc. ESR and CRP) and HAQ; Self-efficacy, RASE |

*Note*. The ‘main outcomes’ column does not include the demographic information collected by each study. ASAS HI = Assessment of SpondyloArthritis International Society Health Index, HCP = health care professional, KOA = Knee Osteoarthritis, F = female, IQR = interquartile range, NRS = numeric rating scale, F2F = face to face, WOMAC = Western Ontario and McMaster Universities Osteoarthritis Index, kg = kilograms, PA = physical activity, IPEQ-W = Incidental and Planned Exercise Questionnaire, HRQoL = health-related quality of life, AQoL = Assessment of Quality of Life Instrument, DASS = Depression, Anxiety, and Stress Scale, SUS = System Usability Scale, HOOS = Hip OA Outcome Score, KOOS = Knee Injury and OA Outcome Score, TUG = Timed “Up & Go” test, SQUASH = Short Questionnaire to Assess Health-Enhancing Physical Activity, ASES = Arthritis Self-efficacy Scale, MIDI = Measurement Instrument for Determinants of Innovations, PT = physiotherapist, JA = Joint Academy, 30CST = 30-second chair stand test, RA = rheumatoid arthritis, IL-6 = interleukin-6, CRP = C-reactive protein, MDA = malondialdehyde, ATP = adenosine tri-phosphate, RAQoL = rheumatoid arthritis quality of life index, ADL = activities of daily living, HAQ = health assessment questionnaire, ArWOMAC = Arabic version of the reduced Western Ontario and McMaster universities Index, FTSST = Five-Times Sit-to-Stand Test, PASE = Physical Activity Scale for the Elderly, CQR = Compliance Questionnaire Rheumatology, ESR = erythrocyte sedimentation rate, DAS28 = Disease Activity Score 28, FIHOA = Functional Index for Hand Osteoarthritis, PSFS = Patient-Specific Function Scale, OMERACT = Outcome Measures in Rheumatological Clinical Trials, OARSI = Osteoarthritis Research Society International, STEP-KOA = Stepped Exercise Program for patients with Knee OsteoArthritis, GOA = generalized osteoarthritis, HAQ-DI = Health Assessment Questionnaire Disability index, SF-36 = Short Form 36 Health Survey, CIS = Checklist Individual Strength; PSK = patient specific complaints questionnaire, GSES = General Self-Efficacy Scale, ICQ = Illness Cognitions Questionnaire, TSK = Tampa scale for kinesiophobia, VAS = visual analogue scale, PSQI = Pittsburgh Sleep Quality Index Self-Report Questionnaire, FIQ = Fibromyalgia Impact Questionnaire, BDI = Beck Depression Inventory, IPAQ = International Physical Activity Questionnaire, T2T = treat-to-target, RAPID3 = Routine Assessment of Patient Index Data 3, SLE = systematic lupus erythematosus, FACIT-F = Functional Assessment of Chronic Illness Therapy Fatigue Scale, 30sSTS = 30-second sit-to-stand test, 5TSTS = five-time Sit-to-Stand test, 30sAC = 30-second arm curl test, 2MST = 2 minute step test, FM = fibromyalgia, FIQR = Revised Fibromyalgia Impact Questionnaire, BPI = Brief Pain Inventory, GAD-7 = Generalized Anxiety Disorder 7-Item, PHQ = Patient Health Questionnaire, HADS = Hospital Anxiety and Depression Scale, PSEQ = Pain Self-Efficacy Questionnaire, SF-12 = Medical Outcomes Study Short Form, WEMWBS = Warwick-Edinburgh Mental Wellbeing Scale, FFMQ = Five Facet Mindfulness Questionnaire, WHO HPQ = World Health Organization Health and Work Performance Questionnaire, WAI-SF = Working Alliance Inventory-Short Form, WAI = Work Ability Index, WLQ = Work Limitations Questionnaire, RA-WIS = Rheumatoid Arthritis Work Instability Scale, BIA = bioelectrical impedance analysis, BMI = body mass index, LDL = low-density lipoprotein cholesterol, TIC-P = The Trimbos and Institute of Medical Technology Assessment Cost Questionnaire for Psychiatry, ISI = Insomnia Severity Index, FFS = Flinders Fatigue Scale, SHI = Sleep Hygiene Index, SPAQ = Sleep Problem Acceptance Questionnaire, CPAQ = Chronic Pain Acceptance Questionnaire, WHOQoL-Bref = World Health Organisation Quality of Life-Bref, IKHOAM = Ibadan Knee/Hip Osteoarthritis Outcome Measure, FAS = Fatigue Assessment Scale, FABQ = Fear Avoidance Belief Questionnaire, RSA = Resilience Scale for Adults, PROMIS-29 = Patient-Reported Outcomes Measurement Information System 29-Item profile measure, PGIC = Patient Global Impression of Change, SPPS = Short Physical Performance Battery, TAM = Technology acceptance model, axSpA = axial spondyloarthritis, ASQoL = AS Quality of Life Index, BASDAI = Bath Ankylosing Spondylitis Disease Activity Index, BASFI = Bath Ankylosing Spondylitis Functional Index, YPAS -= Yale Physical Activity Survey, IRGL = Impact of Rheumatic Diseases on General Health and Lifestyle, RADAI = Rheumatoid Arthritis Disease Activity Index, PRODISQ = PROductivity and Disease Questionnaire, PSS = Perceived Stress Scale, ULS-6 = UCLA Loneliness Scale, MSBS-8 = Multidimensional State Boredom Scale, IPAQ-E = International Physical activity Questionnaire – modified for the elderly, CSQ-8 = Client Satisfaction Questionnaire, PCS = Pain Catastrophizing Scale, CPSS = Chronic Pain Self-Efficacy Scale, CPCI = Chronic Pain Coping Inventory, IA = inflammatory arthritis, ACREU = Arthritis Community Research and Evaluation Unit, IIRS = Illness Intrusiveness Ratings Scale, iCOAP = Intermittent and constant osteoarthritis pain measure, WSSQ = Weight Self-Stigma Questionnaire, 6MWT = 6-min walk test, EARS = Exercise Adherence Rating Scale, GOHT = Guangdong Online Hospital telemedicine, MFI = Multidimensional Fatigue Inventory, MVPA = moderate-to-vigorous PA, MPQ-SF = McGill Pain Questionnaire Short Form, mBQ = Modified Baecke Physical Activity Questionnaire, K-CQR = Korean version of the Compliance Questionnaire-Rheumatology, K-PSQ = Korean version of the Pharmacy Service Questionnaire, BASMI = Bath Ankylosing Spondylitis Metrology Index, WPAI = Work, Productivity and Activity Impairment in AS, EAQ = Exercise Attitude Questionnaire, EMI-2 = Exercise Motivations Inventory-2, RASE = Rheumatoid Arthritis Self-Efficacy questionnaire, PKQ = Patient Knowledge Questionnaire, HLQ = Health Literacy Questionnaire, CDAI = Clinical Disease Activity Index, MDHAQ = Multidimensional Health Assessment Questionnaire, FREKAQ = Fremantle Knee Awareness Questionnaire, OACS = Knee OA and Activity Conceptualisation Scale, PEQ = Participant Experience Questionnaire, PBQ = Pain Beliefs Questionnaire, rNPQ = revised Neurophysiology of Pain Questionnaire, PAM = Patient Activation Measure, ENAT = Educational Needs Assessment Tool, CD-RISC-10 = 10-item Connor-Davidson resilience scale, SRSS = Satisfaction with received social support, WHYMPI = West Haven Yale multidimensional pain inventory, MSK-HQ = Arthritis Research UK Musculoskeletal Health Questionnaire, MSK-USS = musculoskeletal ultrasound scan, SDAI = simplified disease activity index, SECD6 = 6-item self-efficacy scale for chronic diseases, ABC = Awareness-Body-Chart, LupusQoL = Lupus Quality of Life, PEMAT = Patient Education Assessment Tool, SEMCD = , Self-Efficacy for Managing Chronis Disease, SAAS = Social Appearance Anxiety Scale, AUDIT = Alcohol Use Disorders Identification Test, SOPA = Survey of Pain Attitudes, ISAK = International Society for the Advancement of Kinanthropometry, BAS-G = Bath Ankylosing Spondylitis Patient Global Score, AIMS2 = Arthritis Impact Measurement Scales 2, CES-D = Center for Epidemiologic Studies Depression Scale, QLS = Quality of Life Scale, RADAR = Rapid Assessment of Disease Activity in Rheumatology, SPS = Social Provisions Scale, LS-3 = Los Angeles Loneliness Scale version 3, PASIPD = Physical Activity Scale for Individuals with Physical Disabilities, MOCS-A = Measures of Current Status – Part A, CEQ = Credibility and Expectancy Questionnaire, MPGIC = Modified Patient Global Impression of Change, heiQ = Health Education Impact Questionnaire, MI = Motivational Interviewing, MobMPATI = Mobile Motivational Activity Targeted Intervention, VAS-GH = visual analogue scale Global Health, HAMIS = Hand Mobility in Scleroderma test, ROM = range of movement, QUEST 2.0 = Quebec User Evaluation of Satisfaction with Assistive Technology, PIADS = Psychological Impact of Assistive Devices Scale, IPPA = Individually Prioritised Problem Assessment, RAID = rheumatoid arthritis impact of disease, AUSCAN = Australian/Canadian Hand Osteoarthritis Index, NHPT = Nine Hole Peg Test, MHQ = Michigan Hand Outcome Questionnaire, 1SWT = incremental shuttle walk test, mCTSIB = modified Clinical Test of Sensory Interaction on Balance, LOS = limits of stability, ASDAS = Ankylosing Spondylitis Disease Activity Score, MAAS = Mindfulness Attention Awareness Scale, PGA = Patient Global Assessment, SRBAI = Self-Report Behavioural Automaticity Index, PASS = Patient Acceptability Symptom State, HLS-EU-Q16 European Health Literacy Survey, eHEALS = eHealth Literacy Scale, SMI = skeletal muscle index, QUIPA = Quality Indicators Questionnaire for Physiotherapy Management of Hip and Knee Osteoarthritis, FSS = Fatigue Severity Scale

**References**

[1] Acar Y, Ilçin N, Sarı İ. The effects of tele-yoga in ankylosing spondylitis patients: a randomized controlled trial. J Integr Complement Med 2023;29:727-37.

[2] Achmad A, Suharjono S, Soeroso J, Suprapti B, Siswandono S, Pristianty L, et al. Self-education program for osteoarthritis reduces sodium intake, knee joint pain, and serum interleukin-17A level in osteoarthritis patients. Open Access Maced J Med Sci 2022;10:2633–8.

[3] Adly AS, Adly AS, Adly MS. Effects of laser acupuncture tele-therapy for rheumatoid arthritis elderly patients. Lasers Med Sci 2022;37:499-504.

[4] Adly AS, Adly AS, Adly MS, Ali MF. A novel approach utilizing laser acupuncture teletherapy for management of elderly-onset rheumatoid arthritis: A randomized clinical trial. J Telemed Telecare 2021;27:298-306.

[5] Aily JB, Barton CJ, Mattiello SM, De Oliveira Silva D, De Noronha M. Telerehabilitation for knee osteoarthritis in Brazil: a feasibility study. Int J Telerehabil 2020;12:137-48.

[6] Aily JB, de Almeida AC, de Noronha M, Mattiello SM. Effects of a periodized circuit training protocol delivered by telerehabilitation compared to face-to-face method for knee osteoarthritis: a protocol for a non-inferiority randomized controlled trial. Trials 2021;22:887.

[7] Alasfour M, Almarwani M. The effect of innovative smartphone application on adherence to a home-based exercise programs for female older adults with knee osteoarthritis in Saudi Arabia: a randomized controlled trial. Disabil Rehabil 2022;44:2420-7.

[8] Allam A, Kostova Z, Nakamoto K, Schulz PJ. The effect of social support features and gamification on a Web-based intervention for rheumatoid arthritis patients: randomized controlled trial. J Med Internet Res 2015;17:e14.

[9] Allen KD, Bongiorni D, Caves K, Coffman CJ, Floegel TA, Greysen HM, et al. STepped exercise program for patients with knee OsteoArthritis (STEP-KOA): protocol for a randomized controlled trial. BMC Musculoskelet Disord 2019;20:254.

[10] Allen KD, Woolson S, Hoenig HM, Bongiorni D, Byrd J, Caves K, et al. Stepped exercise program for patients with knee osteoarthritis : a randomized controlled trial. Ann Intern Med 2021;174:298-307.

[11] Kaufman BG, Allen KD, Coffman CJ, Woolson S, Caves K, Hall K, et al. Cost and quality of life outcomes of the stepped exercise program for patients with knee osteoArthritis trial. Value Health 2022;25:614-21.

[12] Azma K, RezaSoltani Z, Rezaeimoghaddam F, Dadarkhah A, Mohsenolhosseini S. Efficacy of tele-rehabilitation compared with office-based physical therapy in patients with knee osteoarthritis: A randomized clinical trial. J Telemed Telecare 2018;24:560-5.

[13] Bennell KL, Egerton T, Bills C, Gale J, Kolt GS, Bunker SJ, et al. Addition of telephone coaching to a physiotherapist-delivered physical activity program in people with knee osteoarthritis: a randomised controlled trial protocol. BMC Musculoskelet Disord 2012;13:246.

[14] Bennell KL, Campbell PK, Egerton T, Metcalf B, Kasza J, Forbes A, et al. Telephone coaching to enhance a home-based physical activity program for knee osteoarthritis: a randomized clinical trial. Arthritis Care Res (Hoboken) 2017;69:84-94.

[15] Bennell KL, Jones SE, Hinman RS, McManus F, Lamb KE, Quicke JG, et al. Effectiveness of a telehealth physiotherapist-delivered intensive dietary weight loss program combined with exercise in people with knee osteoarthritis and overweight or obesity: study protocol for the POWER randomized controlled trial. BMC Musculoskelet Disord 2022;23:733.

[16] Bennell KL, Keating C, Lawford BJ, Kimp AJ, Egerton T, Brown C, et al. Better Knee, Better Me™: effectiveness of two scalable health care interventions supporting self-management for knee osteoarthritis - protocol for a randomized controlled trial. BMC Musculoskelet Disord 2020;21:160.

[17] Bennell KL, Lawford BJ, Keating C, Brown C, Kasza J, Mackenzie D, et al. Comparing video-based, telehealth-delivered exercise and weight loss programs with online education on outcomes of knee osteoarthritis : a randomized trial. Ann Intern Med 2022;175:198-209.

[18] Lawford BJ, Bennell KL, Jones SE, Keating C, Brown C, Hinman RS. "It's the single best thing I've done in the last 10 years": a qualitative study exploring patient and dietitian experiences with, and perceptions of, a multi-component dietary weight loss program for knee osteoarthritis. Osteoarthritis Cartilage 2021;29:507-17.

[19] Harris A, Hinman RS, Lawford BJ, Egerton T, Keating C, Brown C, et al. Cost-effectiveness of telehealth-delivered exercise and dietary weight loss programs for knee osteoarthritis within a twelve-month randomized trial. Arthritis Care Res (Hoboken) 2023;75:1311-9.

[20] Bossen D, Kloek C, Snippe HW, Dekker J, de Bakker D, Veenhof C. A blended intervention for patients with knee and hip osteoarthritis in the physical therapy practice: development and a pilot study. JMIR Res Protoc 2016;5:e32.

[21] Kloek CJ, Bossen D, Veenhof C, van Dongen JM, Dekker J, de Bakker DH. Effectiveness and cost-effectiveness of a blended exercise intervention for patients with hip and/or knee osteoarthritis: study protocol of a randomized controlled trial. BMC Musculoskelet Disord 2014;15:269.

[22] Kloek CJJ, Bossen D, Spreeuwenberg PM, Dekker J, de Bakker DH, Veenhof C. Effectiveness of a blended physical therapist intervention in people with hip osteoarthritis, knee osteoarthritis, or both: a cluster-randomized controlled trial. Phys Ther 2018;98:560-70.

[23] Kloek CJJ, PT, Bossen D, PT, de Vries HJ, de Bakker DH, Veenhof C, Dekker J. Physiotherapists' experiences with a blended osteoarthritis intervention: a mixed methods study. Physiother Theory Pract 2020;36:572-9.

[24] Kloek CJJ, van Dongen JM, de Bakker DH, Bossen D, Dekker J, Veenhof C. Cost-effectiveness of a blended physiotherapy intervention compared to usual physiotherapy in patients with hip and/or knee osteoarthritis: a cluster randomized controlled trial. BMC Public Health 2018;18:1082.

[25] de Vries HJ, Kloek CJJ, de Bakker DH, Dekker J, Bossen D, Veenhof C. Determinants of adherence to the online component of a blended intervention for patients with hip and/or knee osteoarthritis: a mixed methods study embedded in the e-Exercise trial. Telemed J E Health 2017;23:1002-10.

[26] Camerini L, Camerini AL, Schulz PJ. Do participation and personalization matter? A model-driven evaluation of an Internet-based patient education intervention for fibromyalgia patients. Patient Educ Couns 2013;92:229-34.

[27] Clayton C, Feehan L, Goldsmith CH, Miller WC, Grewal N, Ye J, et al. Feasibility and preliminary efficacy of a physical activity counseling intervention using Fitbit in people with knee osteoarthritis: the TRACK-OA study protocol. Pilot Feasibility Stud 2015;1:30.

[28] Li LC, Sayre EC, Xie H, Clayton C, Feehan LM. A community-based physical activity counselling program for people with knee osteoarthritis: feasibility and preliminary efficacy of the track-OA study. JMIR Mhealth Uhealth 2017;5:e86.

[29] Li LC, Sayre EC, Xie H, Falck RS, Best JR, Liu-Ambrose T, et al. Efficacy of a community-based technology-enabled physical activity counseling program for people with knee osteoarthritis: proof-of-concept study. J Med Internet Res 2018;20:e159.

[30] Li LC, Feehan LM, Xie H, Lu N, Shaw CD, Gromala D, et al. Effects of a 12-week multifaceted wearable-based program for people with knee osteoarthritis: randomized controlled trial. JMIR Mhealth Uhealth 2020;8:e19116.

[31] Cuperus N, Hoogeboom TJ, Kersten CC, den Broeder AA, Vlieland TP, van den Ende CH. Randomized trial of the effectiveness of a non-pharmacological multidisciplinary face-to-face treatment program on daily function compared to a telephone-based treatment program in patients with generalized osteoarthritis. Osteoarthritis Cartilage 2015;23:1267-75.

[32] Cuperus N, van den Hout WB, Hoogeboom TJ, van den Hoogen FH, Vliet Vlieland TP, van den Ende CH. Cost-utility and cost-effectiveness analyses of face-to-face versus telephone-based nonpharmacologic multidisciplinary treatments for patients with generalized osteoarthritis. Arthritis Care Res (Hoboken) 2016;68:502-10.

[33] da Costa BO, Andrade LS, Botton CE, Alberton CL. Effects of a telehealth stretching exercise program on pain, sleep, depression, and functionality of women with fibromyalgia during the COVID-19 pandemic: a randomized clinical trial. Sustainability 2023;15:2604.

[34] Dahlberg LE, Grahn D, Dahlberg JE, Thorstensson CA. A web-based platform for patients with osteoarthritis of the hip and knee: a pilot study. JMIR Res Protoc 2016;5:e115.

[35] Nero H, Dahlberg J, Dahlberg LE. A 6-week web-based osteoarthritis treatment program: observational quasi-experimental study. J Med Internet Res 2017;19:e422.

[36] Cronström A, Dahlberg LE, Nero H, Ericson J, Hammarlund CS. 'I would never have done it if it hadn't been digital': a qualitative study on patients' experiences of a digital management programme for hip and knee osteoarthritis in Sweden. BMJ Open 2019;9:e028388.

[37] Ekman B, Nero H, Lohmander LS, Dahlberg LE. Costing analysis of a digital first-line treatment platform for patients with knee and hip osteoarthritis in Sweden. PLoS One 2020;15:e0236342.

[38] Dahlberg LE, Dell'Isola A, Lohmander LS, Nero H. Improving osteoarthritis care by digital means - Effects of a digital self-management program after 24- or 48-weeks of treatment. PLoS One 2020;15:e0229783.

[39] Jönsson T, Dell'Isola A, Lohmander LS, Wagner P, Cronström A. Comparison of face-to-face vs digital delivery of an osteoarthritis treatment program for hip or knee osteoarthritis. JAMA Netw Open 2022;5:e2240126.

[40] Farley S, Libman B, Edwards M, Possidente CJ, Kennedy AG. Nurse telephone education for promoting a treat-to-target approach in recently diagnosed rheumatoid arthritis patients: a pilot project. Musculoskeletal Care 2019;17:156-60.

[41] Ferwerda M, van Beugen S, van Middendorp H, Spillekom-van Koulil S, Donders ART, Visser H, et al. A tailored-guided internet-based cognitive-behavioral intervention for patients with rheumatoid arthritis as an adjunct to standard rheumatological care: results of a randomized controlled trial. Pain 2017;158:868-78.

[42] Ferwerda M, van Beugen S, van Middendorp H, Visser H, Vonkeman H, Creemers M, et al. Tailored, therapist-guided internet-based cognitive behavioral therapy compared to care as usual for patients with rheumatoid arthritis: economic evaluation of a randomized controlled trial. J Med Internet Res 2018;20:e260.

[43] Frade S, O'Neill S, Walsh S, Campbell C, Greene D, Bird SP, et al. Telehealth-supervised exercise in systemic lupus erythematosus: a pilot study. Lupus 2023;32:508-20.

[44] García-Perea E, Pedraz-Marcos A, Martínez-Rodríguez SH, Otones-Reyes P, Palmar-Santos AM. Effectiveness of a fibromyalgia online nursing consultation in the quality of life: a randomized controlled trial. Pain Manag Nurs 2022;23:478-85.

[45] Godziuk K, Prado CM, Forhan M. Protocol for the POMELO (Prevention Of MusclE Loss in Osteoarthritis) randomized pilot feasibility trial. Osteoarthr Cartil Open 2022;4:100312.

[46] Godziuk K, Prado CM, Quintanilha M, Forhan M. Acceptability and preliminary effectiveness of a single-arm 12-week digital behavioral health intervention in patients with knee osteoarthritis. BMC Musculoskelet Disord 2023;24:129.

[47] Gohir SA, Greenhaff P, Abhishek A, Valdes AM. Evaluating the efficacy of Internet-Based Exercise programme Aimed at Treating knee Osteoarthritis (iBEAT-OA) in the community: a study protocol for a randomised controlled trial. BMJ Open 2019;9:e030564.

[48] Gohir SA, Eek F, Kelly A, Abhishek A, Valdes AM. Effectiveness of Internet-Based Exercises Aimed at Treating Knee Osteoarthritis: The iBEAT-OA Randomized Clinical Trial. JAMA Netw Open 2021;4:e210012.

[49] Hall M, Spiers L, Knox G, Hinman RS, Sumithran P, Bennell KL. Feasibility of exercise and weight management for people with hip osteoarthritis and overweight or obesity: A pilot study. Osteoarthr Cartil Open 2021;3:100174.

[50] Hall M, Hinman RS, Knox G, Spiers L, Sumithran P, Murphy NJ, et al. Effects of adding a diet intervention to exercise on hip osteoarthritis pain: protocol for the ECHO randomized controlled trial. BMC Musculoskelet Disord 2022;23:215.

[51] Hennig T, Hæhre L, Hornburg VT, Mowinckel P, Norli ES, Kjeken I. Effect of home-based hand exercises in women with hand osteoarthritis: a randomised controlled trial. Ann Rheum Dis 2015;74:1501-8.

[52] Hernando-Garijo I, Ceballos-Laita L, Mingo-Gómez MT, Medrano-de-la-Fuente R, Estébanez-de-Miguel E, Martínez-Pérez MN, et al. Immediate effects of a telerehabilitation program based on aerobic exercise in women with fibromyalgia. Int J Environ Res Public Health 2021;18:2075.

[53] Hernando-Garijo I, Medrano-de-la-Fuente R, Jiménez-Del-Barrio S, Mingo-Gómez MT, Hernández-Lázaro H, Lahuerta-Martin S, et al. Effects of a telerehabilitation program in women with fibromyalgia at 6-month follow-up: secondary analysis of a randomized clinical trial. Biomedicines 2022;10:3024.

[54] Hinman RS, Kimp AJ, Campbell PK, Russell T, Foster NE, Kasza J, et al. Technology versus tradition: a non-inferiority trial comparing video to face-to-face consultations with a physiotherapist for people with knee osteoarthritis. Protocol for the PEAK randomised controlled trial. BMC Musculoskelet Disord 2020;21:522.

[55] Hinman RS, Lawford BJ, Campbell PK, Briggs AM, Gale J, Bills C, et al. Telephone-delivered exercise advice and behavior change support by physical therapists for people with knee osteoarthritis: protocol for the telecare randomized controlled trial. Phys Ther 2017;97:524-36.

[56] Hinman RS, Campbell PK, Lawford BJ, Briggs AM, Gale J, Bills C, et al. Does telephone-delivered exercise advice and support by physiotherapists improve pain and/or function in people with knee osteoarthritis? Telecare randomised controlled trial. Br J Sports Med 2020;54:790-7.

[57] Lawford BJ, Delany C, Bennell KL, Hinman RS. "I was really sceptical...But it worked really well": a qualitative study of patient perceptions of telephone-delivered exercise therapy by physiotherapists for people with knee osteoarthritis. Osteoarthritis Cartilage 2018;26:741-50.

[58] Lawford BJ, Delany C, Bennell KL, Hinman RS. "I was really pleasantly surprised": firsthand experience and shifts in physical therapist perceptions of telephone-delivered exercise therapy for knee osteoarthritis-a qualitative study. Arthritis Care Res (Hoboken) 2019;71:545-57.

[59] Lawford BJ, Bennell KL, Campbell PK, Kasza J, Hinman RS. Therapeutic alliance between physical therapists and patients with knee osteoarthritis consulting via telephone: a longitudinal study. Arthritis Care Res (Hoboken) 2020;72:652-60.

[60] Lawford BJ, Bennell KL, Campbell PK, Kasza J, Hinman RS. Association between therapeutic alliance and outcomes following telephone-delivered exercise by a physical therapist for people with knee osteoarthritis: secondary analyses from a randomized controlled trial. JMIR Rehabil Assist Technol 2021;8:e23386.

[61] Hinman RS, Nelligan RK, Campbell PK, Kimp AJ, Graham B, Merolli M, et al. Exercise adherence Mobile app for Knee Osteoarthritis: protocol for the MappKO randomised controlled trial. BMC Musculoskelet Disord 2022;23:874.

[62] Hoving JL, Zoer I, van der Meer M, van der Straaten Y, Logtenberg-Rutten C, Kraak-Put S, et al. E-health to improve work functioning in employees with rheumatoid arthritis in rheumatology practice: a feasibility study. Scand J Rheumatol 2014;43:481-7.

[63] Hsu YI, Chen YC, Lee CL, Chang NJ. Effects of diet control and telemedicine-based resistance exercise intervention on patients with obesity and knee osteoarthritis: a randomized control trial. Int J Environ Res Public Health 2021;18:7744.

[64] Huang Z, Pan X, Deng W, Huang Z, Huang Y, Huang X, et al. Implementation of telemedicine for knee osteoarthritis: study protocol for a randomized controlled trial. Trials 2018;19:232.

[65] Jacobs CA, Mace RA, Greenberg J, Popok PJ, Reichman M, Lattermann C, et al. Development of a mind body program for obese knee osteoarthritis patients with comorbid depression. Contemp Clin Trials Commun 2021;21:100720.

[66] Mace RA, Greenberg J, Lemaster N, Duarte B, Penn T, Kanaya M, et al. Live video mind-body program for patients with knee osteoarthritis, comorbid depression, and obesity: development and feasibility pilot study. JMIR Form Res 2022;6:e34654.

[67] Jakiela JT, Voinier D, Hinman RS, Copson J, Schmitt LA, Leonard TR, et al. Comparing an expanded versus brief telehealth physical therapist intervention for knee osteoarthritis: study protocol for the delaware PEAK randomized controlled trial. Phys Ther 2023;103:pzac139.

[68] Khan F, Granville N, Malkani R, Chathampally Y. Health-related quality of life improvements in systemic lupus erythematosus derived from a digital therapeutic plus tele-health coaching intervention: randomized controlled pilot trial. J Med Internet Res 2020;22:e23868.

[69] Lee EL, Jang MH, Lee BJ, Han SH, Lee HM, Choi SU, et al. Home-based remote rehabilitation leads to superior outcomes for older women with knee osteoarthritis: a randomized controlled trial. J Am Med Dir Assoc 2023;24:1555-61.

[70] Luo H, Ma Q, Song J, Peng J, Song Y, Chen G, et al. An m-health intervention for rheumatoid arthritis in China ("Rheumatism Center" app): study protocol for a prospective randomized controlled yrial. Nurs Open 2022;9:2915-24.

[71] McCurry SM, Von Korff M, Morin CM, Cunningham A, Pike KC, Thakral M, et al. Telephone interventions for co-morbid insomnia and osteoarthritis pain: the osteoarthritis and therapy for sleep (OATS) randomized trial design. Contemp Clin Trials 2019;87:105851.

[72] McCurry SM, Zhu W, Von Korff M, Wellman R, Morin CM, Thakral M, et al. Effect of telephone cognitive behavioral therapy for insomnia in older adults with osteoarthritis pain: a randomized clinical trial. JAMA Intern Med 2021;181:530-8.

[73] Yeung K, Zhu W, McCurry SM, Von Korff M, Wellman R, Morin CM, et al. Cost-effectiveness of telephone cognitive behavioral therapy for osteoarthritis-related insomnia. J Am Geriatr Soc 2022;70:188-99.

[74] Moutzouri M, Gioftsos G. How effective is a blended web-based rehabilitation for improving pain, physical activity, and knee function of patients with knee osteoarthritis? Study protocol for a randomized control trial. PLoS One 2022;17:e0268652.

[75] Müskens WD, Rongen-van Dartel SAA, Vogel C, Huis A, Adang EMM, van Riel PLCM. Telemedicine in the management of rheumatoid arthritis: maintaining disease control with less health-care utilization. Rheumatol Adv Pract 2021;5:rkaa079.

[76] Nero H, Ranstam J, Kiadaliri AA, Dahlberg LE. Evaluation of a digital platform for osteoarthritis treatment: study protocol for a randomised clinical study. BMJ Open 2018;8:e022925.

[77] Nordlund J, Henry RS, Kwakkenbos L, Carrier M, Levis B, Nielson WR, et al. The scleroderma patient-centered intervention network self-management (SPIN-SELF) program: protocol for a two-arm parallel partially nested randomized controlled feasibility trial with progression to full-scale trial. Trials 2021;22:856.

[78] O'Brien KM, Wiggers J, Williams A, Campbell E, Wolfenden L, Yoong S, et al. Randomised controlled trial of referral to a telephone-based weight management and healthy lifestyle programme for patients with knee osteoarthritis who are overweight or obese: a study protocol. BMJ Open 2016;6:e010203.

[79] O'Brien KM, Wiggers J, Williams A, Campbell E, Hodder RK, Wolenden L, et al. Telephone-based weight loss support for patients with knee osteoarthritis: a pragmatic randomised controlled trial. Osteoarthritis Cartilage 2018;26:485-94.

[80] O'Brien KM, van Dongen JM, Williams A, Kamper SJ, Wiggers J, Hodder RK, et al. Economic evaluation of telephone-based weight loss support for patients with knee osteoarthritis: a randomised controlled trial. BMC Public Health 2018;18:1408.

[81] Østerås N, Hagen KB, Grotle M, Sand-Svartrud A, Mowinckel P, Aas E, et al. Exercise programme with telephone follow-up for people with hand osteoarthritis - protocol for a randomised controlled trial. BMC Musculoskelet Disord 2014;15:82.

[82] Østerås N, Hagen KB, Grotle M, Sand-Svartrud AL, Mowinckel P, Kjeken I. Limited effects of exercises in people with hand osteoarthritis: results from a randomized controlled trial. Osteoarthritis Cartilage 2014;22:1224-33.

[83] Paolucci T, de Sire A, Ferrillo M, di Fabio D, Molluso A, Patruno A, et al. Telerehabilitation proposal of mind-body technique for physical and psychological outcomes in patients with fibromyalgia. Front Physiol 2022;13:917956.

[84] Park JE, Lee JE, Moon BK, Lee H, Park S, Kim S, et al. Impact of a pharmaceutical care service for patients with rheumatoid arthritis using a customised mobile device (the PROUD trial): study protocol for a randomised controlled trial. BMJ Open.2022;12:e061917.

[85] Pasyar N, Sam A, Rivaz M, Nazarinia M. A smartphone-based supportive counseling on health anxiety and acceptance of disability in Systemic Lupus Erythematosus patients: a randomized clinical trial. Patient Educ Couns 2023;110:107676.

[86] Patel KV, Hoffman EV, Phelan EA, Gell NM. Remotely Delivered Exercise to Rural Older Adults With Knee Osteoarthritis: A Pilot Study. ACR Open Rheumatol 2022;4:735-44.

[87] Paul L, Coulter EH, Cameron S, McDonald MT, Brandon M, Cook D, et al. Web-based physiotherapy for people with axial spondyloarthritis (WEBPASS) - a study protocol. BMC Musculoskelet Disord. 2016;17:360.

[88] Paul L, McDonald MT, McConnachie A, Siebert S, Coulter EH. Online physiotherapy for people with axial spondyloarthritis: quantitative and qualitative data from a cohort study. Rheumatol Int 2024;44:145-56.

[89] Pedley R, Dean LE, Choy E, Gaffney K, Ijaz T, Kay L, et al. Feasibility, acceptability and change in health following a telephone-based cognitive behaviour therapy intervention for patients with axial spondyloarthritis. Rheumatol Adv Pract. 2021;5:rkaa063.

[90] Piga M, Tradori I, Pani D, Barabino G, Dessì A, Raffo L, et al. Telemedicine applied to kinesiotherapy for hand dysfunction in patients with systemic sclerosis and rheumatoid arthritis: recovery of movement and telemonitoring technology. J Rheumatol 2014;41:1324-33.

[91] Pani D, Piga M, Barabino G, Crabolu M, Uras S, Mathieu A, et al. Home tele-rehabilitation for rheumatic patients: impact and satisfaction of care analysis. J Telemed Telecare 2017;23:292-300.

[92] Raad T, George E, Griffin A, Larkin L, Fraser A, Kennedy N, et al. A randomised controlled trial of a mediterranean dietary intervention for adults with rheumatoid arthritis (MEDRA): study protocol. Contemp Clin Trials Commun 2022;28:100919.

[93] Raad T, Griffin A, George ES, Larkin L, Fraser A, Kennedy N, et al. Experience and perceptions among rheumatoid arthritis patients following a telehealth-delivered dietary intervention: a qualitative study. Rheumatol Int 2023;43:1913-24.

[94] Raunsbæk Knudsen L, Lomborg K, Ndosi M, Hauge EM, de Thurah A. The effectiveness of e-learning in patient education delivered to patients with rheumatoid arthritis: The WebRA study-protocol for a pragmatic randomised controlled trial. BMC Rheumatol 2021;5:57.

[95] Rodríguez Sánchez-Laulhé P, Biscarri-Carbonero Á, Suero-Pineda A, Luque-Romero LG, Barrero García FJ, Blanquero J, et al. The effects of a mobile app-delivered intervention in people with symptomatic hand osteoarthritis: a pragmatic randomized controlled trial. Eur J Phys Rehabil Med 2023;59:54–64.

[96] Rodríguez-Sánchez-Laulhé P, Luque-Romero LG, Blanquero J, Suero-Pineda A, Biscarri-Carbonero Á, Barrero-García FJ, et al. A mobile app using therapeutic exercise and education for self-management in patients with hand rheumatoid arthritis: a randomized controlled trial protocol. Trials 2020;21(1):777.

[97] Rodríguez Sánchez-Laulhé P, Luque-Romero LG, Barrero-García FJ, Biscarri-Carbonero Á, Blanquero J, Suero-Pineda A, et al. An exercise and educational and self-management program delivered with a smartphone app (CareHand) in adults with rheumatoid arthritis of the hands: randomized controlled trial. JMIR Mhealth Uhealth 2022;10:e35462.

[98] Smarr KL, Musser DR, Shigaki CL, Johnson R, Hanson KD, Siva C. Online self-management in rheumatoid arthritis: a patient-centered model application. Telemed J E Health 2011;17:104-10.

[99] Shigaki CL, Smarr KL, Siva C, Ge B, Musser D, Johnson R. RAHelp: an online intervention for individuals with rheumatoid arthritis. Arthritis Care Res (Hoboken) 2013;65:1573-81.

[100] Singh J, Jha M, Metri K, Mohanty S, Singh A, Tekur P. A study protocol for a randomised controlled trial on the efficacy of yoga as an adjuvant therapy for patients with Ankylosing spondylitis amidst COVID-19 pandemic. Adv Integr Med 2022;9:75-9.

[101] Singh J, Metri K, Tekur P, Mohanty S, Singh A, Raghuram N. Tele-yoga in the management of ankylosing spondylitis amidst COVID pandemic: A prospective randomized controlled trial. Complement Ther Clin Pract 2023;50:101672.

[102] Song Y, Reifsnider E, Zhao S, Xie X, Chen H. A randomized controlled trial of the effects of a telehealth educational intervention on medication adherence and disease activity in rheumatoid arthritis patients. J Adv Nurs 2020;76:1172-81.

[103] Song Y, Reifsnider E, Chen Y, Wang Y, Chen H. The impact of a theory-based mhealth intervention on disease knowledge, self-efficacy, and exercise adherence among ankylosing spondylitis patients: randomized controlled trial. J Med Internet Res 2022;24:e38501.

[104] Song Y, Xie X, Chen Y, Wang Y, Yang H, Nie A, et al. The effects of WeChat-based educational intervention in patients with ankylosing spondylitis: a randomized controlled trail. Arthritis Res Ther 2021;23:72.

[105] Song K, Zhu S, Xiang X, Wang L, Xie S, Liu H, et al. An evidence-based tailored eHealth patient education tool for patients with knee osteoarthritis: protocol for a randomized controlled trial. BMC Musculoskelet Disord 2022;23:274.

[106] Stanton TR, Karran EL, Butler DS, Hull MJ, Schwetlik SN, Braithwaite FA, et al. A pain science education and walking program to increase physical activity in people with symptomatic knee osteoarthritis: a feasibility study. Pain Rep 2020;5:e830.

[107] Stanton TR, Braithwaite FA, Butler D, Moseley GL, Hill C, Milte R, et al. The EPIPHA-KNEE trial: explaining pain to target unhelpful pain beliefs to increase physical activity in knee osteoarthritis - a protocol for a multicentre, randomised controlled trial with clinical- and cost-effectiveness analysis. BMC Musculoskelet Disord 2021;22:738.

[108] Tahran Ö, Ersöz Hüseyinsinoğlu B, Yolcu G, Karadağ Saygı E. Internet-based basic body awareness therapy in fibromyalgia syndrome: A report of three cases. Mod Rheumatol Case Rep 2023;7:464-9.

[109] Tam J, Lacaille D, Liu-Ambrose T, Shaw C, Xie H, Backman CL, et al. Effectiveness of an online self-management tool, OPERAS (an On-demand Program to EmpoweR Active Self-management), for people with rheumatoid arthritis: a research protocol. Trials 2019;20:712.

[110] Tore NG, Oskay D, Haznedaroglu S. The quality of physiotherapy and rehabilitation program and the effect of telerehabilitation on patients with knee osteoarthritis. Clin Rheumatol 2023;42:903-15.

[111] Vallejo MA, Ortega J, Rivera J, Comeche MI, Vallejo-Slocker L. Internet versus face-to-face group cognitive-behavioral therapy for fibromyalgia: A randomized control trial. J Psychiatr Res 2015;68:106-13.

[112] Walter MM, Sirard P, Nero H, Hörder H, Dahlberg LE, Tveter AT, et al. Digitally delivered education and exercises for patients with hand osteoarthritis-An observational study. Musculoskeletal Care 2023;21:1154-60.

[113] Wang L, Xie S, Bao T, Zhu S, Liang Q, Wang X, et al. Exercise and education for community-dwelling older participants with knee osteoarthritis: a video-linked programme protocol based on a randomised controlled trial. BMC Musculoskelet Disord 2021;22:470.

[114] Wang XY, Xie SH, Zhang YJ, Zhu S, Zhang R, Wagg L, et al. Effect of IoT-based power cycling and quadriceps training on pain and function in patients with knee osteoarthritis: a randomized controlled trial protocol. Medicine (Baltimore) 2022;101:e31841.

[115] Warmington K, Flewelling C, Kennedy CA, Shupak R, Papachristos A, Jones C, et al. Telemedicine delivery of patient education in remote Ontario communities: feasibility of an advanced clinician practitioner in arthritis care (ACPAC)-led inflammatory arthritis education program. Open Access Rheumatol 2017;9:11-9.

[116] Kennedy CA, Warmington K, Flewelling C, Shupak R, Papachristos A, Jones C, et al. A prospective comparison of telemedicine versus in-person delivery of an interprofessional education program for adults with inflammatory arthritis. J Telemed Telecare 2017;23:197-206.

[117] Weber F, Müller C, Bahns C, Kopkow C, Färber F, Gellert P, et al. Smartphone-assisted training with education for patients with hip and/or knee osteoarthritis (SmArt-E): study protocol for a multicentre pragmatic randomized controlled trial. BMC Musculoskelet Disord 2023;24:221.

[118] Xie SH, Wang Q, Wang LQ, Zhu SY, Li Y, He CQ. The feasibility and effectiveness of internet-based rehabilitation for patients with knee osteoarthritis: a study protocol of randomized controlled trial in the community setting. Medicine (Baltimore) 2020;99:e22961.

[119] Zhang Y, Xie S, Wang X, Song K, Wang L, Zhang R, et al. Effects of internet of things-based power cycling and neuromuscular training on pain and walking ability in elderly patients with KOA: protocol for a randomized controlled trial. Trials 2022;23:1009.

[120] Zhao S, Chen H. Effectiveness of health education by telephone follow-up on self-efficacy among discharged patients with rheumatoid arthritis: a randomised control trial. J Clin Nurs 2019;28:3840-7.
