# Supplementary material for: Rehabilitation Interventions Delivered via Telehealth to Support Self‐Management of Rheumatic and Musculoskeletal Disease: A Scoping Review
Source: Arthritis Rheumatol. 2025 Aug 19;78(1):26–36. doi: 10.1002/art.43277 (PMC12854011; doi:10.1002/art.43277)
Supplement: Supplementary file 4 — Table S1. Preferred Reporting Items for Systematic reviews and Meta‐Analyses extension for Scoping Reviews (PRISMA‐ScR) Checklist [file ART-78-26-s003.docx]

**Table S3**. A summary of intervention content.

|  | **Aspect of rehabilitation** | | | | | | | | | | | | | | | | | | | | **Type of digital technology** | | | | | | | | | |  | **Context** | | | | | |  |
| --- | --- | --- | --- | --- | --- | --- | --- | --- | --- | --- | --- | --- | --- | --- | --- | --- | --- | --- | --- | --- | --- | --- | --- | --- | --- | --- | --- | --- | --- | --- | --- | --- | --- | --- | --- | --- | --- | --- |
|  | General education / self-management intervention | Education | Physical activity intervention | Physiotherapist supported | Diet and nutrition intervention | Sleep intervention | Occupational health intervention (work-related guidance) | Medication adherence intervention | Laser acupuncture intervention | Cognitive behavioural therapy | Pain coping (any mention of) | "Activity pacing" (any mention of) | Relaxation, meditation, mindfulness and breathing | Counselling (any mention of) | Motivational Interviewing | Goal Setting (any mention of) | Citation of a BCT (informed by) | Informed by theory (explicitly states which and how) | Medication / supplement a part of the intervention | Physical resource | Website / web platform | Smartphone application | Social / instant messaging application | Videoconferencing software / service | Video communication with a HCP | Audio communication with a HCP | Text communication with a HCP | Use of video links and other websites | Use of automatic messages, reminders or notifications | Other Device | Miscellaneous | Tech training/education (any mention of) | Face-to-face involvement with HCP | Real-time HCP interaction | Delayed HCP interaction | Group-based / social element | Compulsory important other involvement | Interaction unclear |
|  |  |  |  |  |  |  |  |  |  |  |  |  |  |  |  |  |  |  |  |  |  |  |  |  |  |  |  |  |  |  |  |  |  |  |  |  |  |  |
|  |  |  |  |  |  |  |  |  |  |  |  |  |  |  |  |  |  |  |  |  |  |  |  |  |  |  |  |  |  |  |  |  |  |  |  |  |  |  |
|  |  |  |  |  |  |  |  |  |  |  |  |  |  |  |  |  |  |  |  |  |  |  |  |  |  |  |  |  |  |  |  |  |  |  |  |  |  |  |
|  |  |  |  |  |  |  |  |  |  |  |  |  |  |  |  |  |  |  |  |  |  |  |  |  |  |  |  |  |  |  |  |  |  |  |  |  |  |  |
|  |  |  |  |  |  |  |  |  |  |  |  |  |  |  |  |  |  |  |  |  |  |  |  |  |  |  |  |  |  |  |  |  |  |  |  |  |  |  |
| **References** |  |  |  |  |  |  |  |  |  |  |  |  |  |  |  |  |  |  |  |  |  |  |  |  |  |  |  |  |  |  |  |  |  |  |  |  |  |  |
|  |  |  |  |  |  |  |  |  |  |  |  |  |  |  |  |  |  |  |  |  |  |  |  |  |  |  |  |  |  |  |  |  |  |  |  |  |  |  |
| Deployed studies | | | | | | | | | | | | | | | | | | | | | | | | | | | | | | | | | | | | | | |
| [1] |  |  | X | X |  |  |  |  |  |  |  |  | X |  |  |  |  |  |  |  |  |  |  | X | X |  |  |  |  |  |  | X |  | X |  | X |  |  |
| [2] |  | X |  |  | X |  |  |  |  |  |  |  |  |  |  |  |  |  |  | X |  |  | X |  |  |  |  | X | X |  |  |  | X |  |  |  |  | X |
| [3] |  | X | X |  |  |  |  |  | X |  |  |  |  | X |  |  |  |  | X | X |  | X |  |  | X |  | X | X | X | X |  | X | ? | X | X |  |  | X |
| [4] |  | X | X |  |  |  |  |  | X |  | X |  | X |  |  |  |  |  |  | X |  | X |  |  | X |  | X | X | X | X | X | X | ? | X | X |  | X | X |
| [5] |  | X | X | X |  |  |  |  |  |  |  |  |  |  |  |  |  |  |  | X | X |  |  |  |  | X |  | X |  |  |  |  | X | X |  |  |  |  |
| [7] |  | X | X | X |  |  |  |  |  |  |  |  |  |  |  |  |  |  |  | X |  | X |  |  |  |  |  |  | X |  |  | X | X |  |  |  |  |  |
| [8] | X | X |  |  |  |  |  |  |  |  | X |  |  |  |  |  |  |  |  |  | X |  |  |  |  |  | X | X | X |  | X | X |  | X | X | X |  |  |
| [10] |  | X | X | X |  |  |  |  |  |  |  | X |  |  | X | X |  |  |  | X | X |  |  |  |  | X |  | X |  |  |  | X | X | X |  |  |  |  |
| [12] |  | X | X |  |  |  |  |  |  |  |  |  |  |  |  |  |  |  |  | X |  |  |  |  |  | X |  |  |  |  |  |  | ? | X |  |  |  | X |
| [14] |  | X | X | X |  |  |  |  |  |  | X | X |  |  | X | X |  | X |  | X |  |  |  |  |  | X |  |  |  | X |  |  | X | X |  |  |  |  |
| [17] |  | X | X | X | X |  |  |  |  |  | X | X |  | X | X | X | X | X |  | X | X |  |  | X | X |  |  | X |  | X |  | X |  | X |  |  |  |  |
| [20] |  | X | X | X |  |  |  |  |  |  | X |  | X |  |  |  |  |  |  |  | X |  |  |  |  |  |  | X |  |  |  |  | X |  |  |  |  |  |
| [22] |  | X | X | X |  |  |  |  |  |  | X |  | X |  |  | X |  | X |  |  | X |  |  |  |  |  |  | X | X |  |  | X | X |  |  |  |  |  |
| [26] | X | X |  |  |  |  |  |  |  |  | X |  |  |  |  |  |  |  |  |  | X |  |  |  |  |  | X | X |  |  |  |  |  | X | X | X |  |  |
| [28] |  | X | X | X |  |  |  |  |  |  |  |  |  | X | X | X |  | X |  | X | X |  |  |  |  | X | X |  |  | X |  | X | X | X | X | X |  |  |
| [29] |  | X | X | X |  |  |  |  |  |  |  |  |  | X | X | X |  | X |  | X | X |  |  |  |  | X | X |  |  | X |  | X | X | X | X | X |  |  |
| [30] |  | X | X | X |  |  |  |  |  |  |  |  |  | X | X | X |  | X |  | X | X |  |  |  |  | X | X |  |  | X |  | X | X | X | X | X |  |  |
| [31] |  | X | X | X |  |  |  |  |  |  | X | X |  |  | X | X |  | X |  | X |  |  |  |  |  | X |  |  |  |  |  |  | X | X |  | X |  |  |
| [33] |  | X | X | X |  |  |  |  |  |  |  |  |  |  |  |  |  |  |  |  |  |  | X |  | X |  |  | X | X |  |  |  |  | X |  |  |  |  |
| [34] |  | X | X | X |  |  |  |  |  |  | X |  |  |  |  |  |  |  |  |  | X |  |  |  |  |  | X | X | X |  |  |  |  |  | X |  |  |  |
| [35] |  | X | X | X |  |  |  |  |  |  | X |  |  |  |  |  |  |  |  |  | X |  |  |  |  | X | X | X | X |  |  |  |  | X | X |  |  | X |
| [38] |  | X | X | X |  |  |  |  |  |  | X |  |  |  |  | X |  |  |  |  | X |  |  |  |  | X | X | X | X |  |  |  |  | X | X |  |  | X |
| [40] | X | X |  |  |  |  |  |  |  |  |  |  |  |  |  |  |  |  |  | X |  |  |  |  |  | X |  |  |  |  |  |  |  | X |  |  |  |  |
| [41] |  | X |  |  |  |  |  |  |  | X | X | X | X |  |  | X |  |  |  |  | X |  |  |  |  |  | X |  |  |  |  | X | X |  | X |  |  |  |
| [43] |  |  | X |  |  |  |  |  |  |  |  |  |  |  |  |  |  |  |  | X |  |  |  | X | X |  |  |  |  |  |  |  |  | X |  |  |  |  |
| [44] | X | X |  |  |  |  |  |  |  |  |  |  |  |  |  |  |  |  |  |  | X |  |  |  |  |  | X |  |  |  |  | X |  |  | X |  |  |  |
| [46] |  | X | X |  | X |  |  |  |  |  |  |  | X |  |  | X |  |  |  |  | X |  |  |  | X | X | X | X | X |  |  |  |  | X |  | X |  |  |
| [48] |  | X | X | X |  |  |  |  |  |  | X |  |  |  |  |  |  |  |  |  |  | X |  |  |  | X | X | X | X |  |  |  |  | X | X |  |  | X |
| [49] |  | X | X | X | X |  |  |  |  |  | X | X | X |  | X | X | X |  |  | X |  |  |  | X | X |  |  | X |  |  |  | X |  | X |  |  |  |  |
| [51] |  | X | X |  |  |  |  |  |  |  |  |  |  |  |  |  |  |  |  | X |  |  |  |  |  | X |  |  |  |  |  |  |  | X |  |  |  |  |
| [52] |  | X | X | X |  |  |  |  |  |  |  |  |  |  |  |  |  |  |  |  |  |  |  |  | X |  |  | ? |  |  |  |  | ? | X |  | ? |  | X |
| [56] |  | X | X | X |  |  |  |  |  |  | X | X |  |  | X | X |  | X |  | X | X |  |  |  |  | X |  | X |  |  |  |  |  | X |  |  |  |  |
| [62] |  | X |  |  |  |  | X |  |  |  |  |  |  | X |  | X |  |  |  |  | X |  |  |  |  | X | X |  |  |  |  |  | X | X | X |  |  |  |
| [63] |  | X | X |  | X |  |  |  |  |  |  |  |  |  |  |  |  |  |  | X |  |  | X |  | X | X |  |  |  |  |  |  | X | X |  |  |  |  |
| [66] |  | X | X |  |  |  |  |  |  |  | X | X | X |  |  | X |  |  |  | X | X |  |  | X | X |  |  |  | X |  |  |  |  | X |  | X |  |  |
| [68] |  | X |  |  | X |  |  |  |  |  |  |  |  |  |  | X |  |  | X | Y |  | X |  |  |  | X | ? |  |  |  |  | X |  | X |  |  |  |  |
| [69] |  | X | X | X |  |  |  |  |  |  |  |  |  |  |  |  |  |  |  | X |  | X |  |  | X |  |  |  |  | X |  | X | ? | X |  |  |  | X |
| [72] |  | X |  |  |  | X |  |  |  | X | X |  | X |  |  |  |  | X |  | X |  |  |  |  |  | X |  |  |  |  |  |  |  | X |  |  |  |  |
| [75] | X | X |  |  |  |  |  |  |  |  |  |  |  |  |  |  |  |  |  |  | X |  |  |  |  |  | X |  |  |  |  | X |  |  | X |  |  | X |
| [79] |  | X | X | X | X |  |  |  |  |  |  |  |  |  | X | X |  |  |  | X |  |  |  |  |  | X |  |  |  |  |  |  |  | X |  |  |  |  |
| [82] |  |  | X |  |  |  |  |  |  |  |  |  |  | X | X |  |  | X |  | X |  |  |  |  |  | X |  |  | X |  |  |  | X | X |  | X |  |  |
| [83] |  | X | X | X |  |  |  |  |  |  |  |  | X |  |  |  |  |  |  | X |  |  |  | X | X | ? | X |  |  |  |  |  |  | X | ? | X |  | X |
| [85] | X | X |  |  |  |  |  |  |  |  |  |  | X | X |  |  |  |  |  |  |  |  | X |  |  |  | X | X | X |  |  |  |  | ? | ? | X |  | X |
| [86] |  |  | X |  |  |  |  |  |  |  |  |  |  |  |  |  |  |  |  | X |  |  |  | X | X |  |  |  |  |  |  | X |  | X |  | X |  |  |
| [88] |  | X | X | X |  |  |  |  |  |  |  |  |  |  |  | X | X |  |  |  | X |  |  |  |  | X |  | X |  |  |  | X | X | X |  |  |  |  |
| [89] |  | X |  |  |  |  |  |  |  | X | X |  |  |  |  | X | X |  |  |  |  |  |  |  |  | X |  |  |  |  |  |  |  | X |  |  |  |  |
| [90] |  | X | X |  |  |  |  |  |  |  |  |  |  |  |  |  |  |  |  | X |  |  |  |  |  | X |  |  |  | X |  | X |  | X |  |  |  | X |
| [93] |  | X |  |  | X |  |  |  |  |  |  |  |  | X |  | X |  |  |  | X |  |  |  | X | X | X |  |  |  |  |  |  |  | X |  |  |  |  |
| [95] |  | X | X | X |  |  |  |  |  |  |  |  |  |  |  |  |  |  |  |  |  | X |  |  |  | X |  | X |  |  |  | X |  | X |  |  |  |  |
| [97] |  | X | X | X |  |  |  |  |  |  |  |  |  |  |  |  |  |  |  |  |  | X |  |  |  | X |  | X |  |  |  | X |  | X |  |  |  |  |
| [98] | X | X |  |  |  |  |  |  |  |  | X |  |  | X |  | X |  | X |  | X | X |  |  |  |  | X | X |  | X |  |  | X |  | X | X | X |  |  |
| [99] | X | X |  |  |  |  |  |  |  |  | X |  |  | X |  | X |  | X |  |  | X |  |  |  |  | X | X |  | X |  |  | X |  | X | X | X |  |  |
| [101] |  | X | X |  |  |  |  |  |  |  |  |  | X |  |  |  |  |  |  | X |  |  |  |  | ? | X |  | X |  |  |  |  |  | X |  | ? |  | X |
| [102] |  | X |  |  |  |  |  | X |  |  |  |  |  |  |  | X |  | X |  |  |  |  |  |  |  | X |  |  |  |  |  |  |  | X |  |  |  |  |
| [103] | X | X |  |  |  |  |  |  |  |  |  |  |  |  |  |  |  | X |  |  |  |  | X | X | X |  | ? | X | X |  |  | X |  | X |  |  |  |  |
| [106] |  | X | X | X |  |  |  |  |  |  | X | X |  |  |  | X |  | X |  | X |  |  |  |  |  | X |  | X |  |  |  |  | X | X |  |  |  |  |
| [108] |  |  | X | X |  |  |  |  |  |  |  |  | X |  |  |  |  |  |  |  |  |  |  | X | X |  |  |  |  |  |  |  |  | X |  |  |  |  |
| [110] |  | X | X | X |  |  |  |  |  |  |  |  |  |  |  |  |  |  |  |  |  |  |  | X | X |  |  |  |  |  |  | X |  | X |  | ? |  |  |
| [111] |  | X |  |  |  |  |  |  |  | X | X |  | X |  |  |  |  |  |  |  | X |  |  |  |  |  | X |  | X |  |  |  |  |  | X |  |  |  |
| [112] |  | X | X | X |  |  |  |  |  |  | X |  |  |  |  |  |  |  |  |  |  | X |  |  | X | X | X | X | X |  |  |  |  | X | X | X |  |  |
| [115] | X | X |  | X |  |  |  |  |  |  | X |  |  |  |  |  |  |  |  |  |  |  |  |  | X |  |  |  |  |  |  |  | X | X |  | X |  |  |
| [120] | X | X |  |  |  |  |  |  |  |  |  |  |  |  |  |  |  |  |  |  |  |  |  |  |  | X |  |  |  |  |  |  |  | X |  |  |  |  |
| Protocol only | | | | | | | | | | | | | | | | | | | | | | | | | | | | | | | | | | | | | | |
| [6] |  | X | X | X |  |  |  |  |  |  |  |  |  |  |  |  |  |  |  | X | X |  | X |  |  | X |  | X | X |  |  |  | X | X |  |  |  |  |
| [15] |  | X | X | X | X |  |  |  |  |  | X |  | X |  | X | X |  |  |  | X |  |  |  | X | X |  |  | X |  |  |  | X |  | X |  |  |  |  |
| [45] |  | X | X |  | X |  |  |  |  |  | X |  |  |  |  |  |  |  |  | X |  |  |  |  | X | X |  | X | X |  |  |  | X | X |  | X |  |  |
| [50] |  | X | X | X | X |  |  |  |  |  | X |  |  |  | X | X | X |  |  | X |  |  |  | X | X |  |  | X |  |  |  | X |  | X |  |  |  |  |
| [54] |  | X | X | X |  |  |  |  |  |  | X | X | X |  |  | X | X |  |  | X |  |  |  | X | X |  |  | X |  | X |  | X |  | X |  |  |  |  |
| [61] |  | X | X | X |  |  |  |  |  |  |  |  |  |  |  | X | X |  |  | X |  | X |  | X | X |  |  | X | X |  |  | X |  | X |  |  |  |  |
| [64] | X | X |  |  |  |  |  |  |  |  |  |  |  |  |  |  |  |  | X |  | X |  |  |  | ? | ? | ? |  |  |  |  |  |  | ? | ? |  |  | X |
| [67] |  | X | X | X |  |  |  |  |  |  | X | X |  | X | X | X |  |  |  | X |  |  |  | X | X |  |  |  |  | X |  | X |  | X |  |  |  |  |
| [70] | X | X |  |  |  |  |  |  |  |  |  |  |  |  |  |  |  |  |  |  |  | X |  |  | ? | ? | ? | X | X |  |  |  |  | ? | ? |  |  | X |
| [74] |  | X | X | X |  |  |  |  |  |  | X | X |  |  |  | X |  |  |  | X | X |  |  |  |  | X |  | X |  | X |  | X | X | X |  |  |  |  |
| [76] |  | X | X | X |  |  |  |  |  |  | X |  |  |  |  |  |  |  |  |  | X |  |  |  |  | X | X | X | X |  |  |  |  | X | X |  |  |  |
| [77] | X | X |  |  |  |  |  |  |  |  | X |  |  |  |  | X |  | X |  |  | X |  |  | X | X |  |  | X |  |  |  | X |  | X |  | X |  |  |
| [84] |  | X |  |  |  |  |  | X |  |  |  |  |  | X |  |  |  |  |  |  |  | X |  |  |  | X | X | X | X |  |  | X |  | X | X |  |  |  |
| [94] |  | X |  |  |  |  |  | X |  |  | X |  |  |  |  |  |  | X | X |  | X |  |  |  |  | X |  | X |  |  |  |  | X | X |  |  |  |  |
| [105] | X | X |  | X |  |  |  |  |  |  |  |  |  |  |  |  |  |  |  | X |  |  | X | X | X |  | ? | X | X |  |  | X |  | X |  |  |  |  |
| [107] |  | X | X | X |  |  |  |  |  |  | X | X |  |  |  | X |  | X |  | X |  |  |  | X | X | X |  | X |  |  |  |  | X | X |  |  |  |  |
| [109] |  | X | X | X |  |  |  |  |  |  |  |  |  | X | X | X |  | X |  | X | X |  |  |  |  | X |  |  |  | X |  | X | X | X |  | X |  |  |
| [113] |  | X | X | X |  |  |  |  |  |  |  |  |  |  |  |  |  |  |  |  | X |  |  | X | X |  |  | X | X |  |  |  |  | X |  |  | X |  |
| [114] |  | X | X | X |  |  |  |  |  |  |  |  |  |  |  |  |  |  | X | X |  |  | X |  | X | X |  | X | X |  |  |  |  | X |  |  | X |  |
| [117] |  | X | X | X |  |  |  |  |  |  | X |  |  |  |  | X |  |  |  | X |  | X |  |  | X |  | X | X | X |  |  | X | X | X | X | X |  |  |
| [118] |  | X | X | X |  |  |  |  |  |  |  |  |  |  |  |  |  |  | X | X |  | X |  |  | ? | ? | X | X | X | X |  | X |  | ? | X |  |  | X |
| [119] |  | X | X | X |  |  |  |  |  |  |  |  |  |  |  |  |  |  |  | X |  |  | X |  | X |  |  | X | X |  |  |  |  | X |  |  | X |  |
